# Evidence of synergistic relationships between HIV and Human Papillomavirus (HPV): systematic reviews and meta‐analyses of longitudinal studies of HPV acquisition and clearance by HIV status, and of HIV acquisition by HPV status

**DOI:** 10.1002/jia2.25110

**Published:** 2018-06-05

**Authors:** Katharine J Looker, Minttu M Rönn, Patrick M Brock, Marc Brisson, Melanie Drolet, Philippe Mayaud, Marie‐Claude Boily

**Affiliations:** ^1^ Population Health Sciences Bristol Medical School University of Bristol Bristol UK; ^2^ Department of Infectious Disease Epidemiology Imperial College London London UK; ^3^ Department of Global Health and Population Harvard T.H Chan School of Public Health Boston USA; ^4^ Institute of Biodiversity, Animal Health and Comparative Medicine College of Medical, Veterinary and Life Sciences University of Glasgow Glasgow UK; ^5^ Centre de recherche du CHU de Québec‐Université Laval Axe santé des populations et pratiques optimales en santé Québec Canada; ^6^ Department of Clinical Research Faculty of Infectious and Tropical Diseases London School of Hygiene and Tropical Medicine London UK

**Keywords:** HPV, HIV, sexually transmitted infections, epidemiology, incidence, meta‐analysis, systematic review, humans

## Abstract

**Introduction:**

Observational studies suggest HIV and human papillomavirus (HPV) infections may have multiple interactions. We reviewed the strength of the evidence for the influence of HIV on HPV acquisition and clearance, and the influence of HPV on HIV acquisition.

**Methods:**

We performed meta‐analytic systematic reviews of longitudinal studies of HPV incidence and clearance rate by HIV status (review 1) and of HIV incidence by HPV status (review 2). We pooled relative risk (RR) estimates across studies using random‐effect models. *I* ^2^ statistics and subgroup analyses were used to quantify heterogeneity across estimates and explore the influence of participant and study characteristics including study quality. Publication bias was examined quantitatively with funnel plots and subgroup analysis, as well as qualitatively.

**Results and Discussion:**

In review 1, 37 publications (25 independent studies) were included in the meta‐analysis. HPV incidence (pooled RR = 1.55, 95% CI: 1.29 to 1.88; heterosexual males: pooled RR = 1.95, 95% CI: 1.62, 2.34; females: pooled RR = 1.63, 95% CI: 1.26 to 2.11; men who have sex with men: pooled RR = 1.36, 95% CI: 1.01 to 1.82) and high‐risk HPV incidence (pooled RR = 2.20, 95% CI: 1.90 to 2.54) was approximately doubled among people living with HIV (PLHIV) whereas HPV clearance rate (pooled RR = 0.53, 95% CI: 0.42 to 0.67) was approximately halved. In review 2, 14 publications (11 independent studies) were included in the meta‐analysis. HIV incidence was almost doubled (pooled RR = 1.91, 95% CI 1.38 to 2.65) in the presence of prevalent HPV infection. There was more evidence of publication bias in review 2, and somewhat greater risk of confounding in studies included in review 1. There was some evidence that adjustment for key confounders strengthened the associations for review 2. Misclassification bias by HIV/HPV exposure status could also have biased estimates toward the null.

**Conclusions:**

These results provide evidence for synergistic HIV and HPV interactions of clinical and public health relevance. HPV vaccination may directly benefit PLHIV, and help control both HPV and HIV at the population level in high prevalence settings. Our estimates of association are useful for mathematical modelling. Although observational studies can never perfectly control for residual confounding, the evidence presented here lends further support for the presence of biological interactions between HIV and HPV that have a strong plausibility.

## 1 Introduction

The majority of individuals who are sexually active will acquire human papillomavirus (HPV) infection at some point in their lives, but most will develop the necessary immune response and clear the infection 1, 2. However, in some individuals infection is not cleared, and can progress to dysplasia, which can eventually lead to carcinoma *in situ*, and invasive carcinoma. High‐risk HPV types 16 and 18 account for about 70% of all cervical cancers and precancerous lesions, and a substantial fraction of anal, vulvar, vaginal and penile cancers 1, 3. The global burden of HPV‐related disease is mainly concentrated in resource‐poor settings where 85% of the estimated 528,000 cervical cancer cases and 266,000 deaths occurred in 2012 1, 4. Sub‐Saharan Africa faces a dual disease burden as the region has some of the highest rates of cervical cancer incidence and deaths, 4 and it accounts for approximately 70% of people living with HIV (PLHIV) worldwide 5, 6. HIV is believed to exacerbate the burden of cervical cancer.

Current evidence suggests that HIV and HPV infections may interact in multiple ways 7. Both viruses infect anogenital sites and are influenced by similar risk factors such as number of sexual partners. However, there is also evidence for direct biological and immunological interactions. Invasive cervical carcinoma is an AIDS‐defining illness due to increased risk with immunosuppression 8, 9 (although immunosuppression is not a necessary condition for HPV infection to progress to cancer). Similarly, HPV acquisition risk, persistence of infection and disease progression to intraepithelial neoplasia (such as cervical intraepithelial neoplasia (CIN) and anal intraepithelial neoplasia (AIN)) and cancer may be increased among people with a range of immunodeficiencies, including as a consequence of HIV infection 2. HIV interventions such as medical male circumcision and anti‐retroviral therapy (ART) may directly and indirectly reduce the risk of HPV acquisition and/or disease progression 10, 11. Previous systematic reviews suggest that, similarly to other sexually transmitted infections (STIs), HPV may in turn directly increase HIV susceptibility, either by breaching the epithelial barrier, recruiting HIV target cells to the genital tract, or by generating a proinflammatory local immune milieu 12, 13. However, it is unclear whether increased HIV acquisition depends on HPV type, the number of HPV types present, and time since HPV infection, that is, whether HPV infection is incident, prevalent, or recently cleared, as a consequence of differences in the immune response in each instance.

Quantifying the magnitude of the biological interactions between HIV and HPV is important for many reasons. If HIV and HPV biologically interact together by increasing susceptibility to infection (for HIV and HPV), duration of infection (for HPV), and the severity and risk of developing disease (for HPV), it will facilitate HIV and HPV spread, and increase the burden of HPV infection and diseases among PLHIV. Presence of synergistic interactions would also mean that prevention efforts against one infection could provide indirect population‐level benefits for the other. For example, if HPV vaccination could prevent additional HIV infections and related deaths, this could be particularly relevant for high HIV prevalence countries in Sub‐Saharan Africa and strengthen the case for implementing HPV vaccination programmes in this region. Estimating the size of these interactions is important to parameterize mathematical models and understand how these biological interactions and the rollout of HIV interventions may influence future population‐level trends in HPV and HIV infections and related diseases, patterns of co‐morbidity, and HPV vaccination impact in high HIV prevalence settings.

Here, we present a systematic and meta‐analytic review of all the evidence for the association between HIV exposure and subsequent risk of HPV acquisition and rate of HPV clearance. For completeness, we also updated and re‐analysed two previous meta‐analyses for the association between HPV exposure and subsequent HIV acquisition, which had a latest search date of 31st January 2012 12, 13. In both of our reviews, detailed assessments of the influence of study and participant characteristics including study quality on pooled estimates of the association, and the risk of confounding and publication bias, are presented. These two reviews collectively comprehensively assess the evidence for synergistic interactions between the two infections.

## 2 Methods

The systematic reviews and meta‐analyses were undertaken in accordance with Meta‐analysis of Observational Studies in Epidemiology (MOOSE)^14^ and Preferred Reporting Items for Systematic Reviews and Meta‐Analyses (PRISMA)^15^ guidelines.

### 2.1 Search strategy

PubMed and Embase databases were searched (up to 6th January 2017) to identify longitudinal studies investigating the association between HIV status and subsequent HPV acquisition and clearance (review 1) as well as the effect of HPV status on subsequent HIV acquisition (review 2). We screened publication titles, abstracts and articles for longitudinal studies using a combination of keywords and MeSH terms relating to HPV or cervical/anal/genital neoplasia, cancer, abnormalities or lesions and HIV. Bibliographies of relevant articles were examined for additional references. Details of the search terms used are provided in Appendix S1.

### 2.2 Study selection and data extraction

Publications were examined for eligibility to include prospective cohort studies, randomized controlled trials, and case–control studies nested within a cohort or trial, where the time sequence between HIV and HPV infections was determined, that were published in the English language, and that measured active genital HPV infection by detection of the virus in either cervical, penile or anal swabs or cervico‐vaginal lavage fluid using a test based on HPV DNA identification. We excluded studies that only measured HPV antibodies, which do not distinguish between current and past infection. No further exclusions were made on the basis of study quality, which was instead assessed in subgroup analyses and qualitatively. The main associations of interest were the relative risk (RR) of acquiring a new HPV infection or clearing an existing HPV infection (outcomes) by HIV serostatus (exposure) (review 1), and the RR of acquiring HIV infection (outcome) by HPV status (either prevalent, recent/incident or recently cleared HPV infection) (exposure) (review 2). Given the large number of HPV types, we used the following standard HPV categories: (any) HPV, high‐risk HPV (HR‐HPV), low‐risk HPV (LR‐HPV) using the HPV groupings stated in the publications, and single HPV types included in the nonavalent vaccine (HPV‐6/11/16/18/31/33/45/52/58). Where available, estimates for the association between HIV and number of HPV types (dose–response for review 2), and estimates by CD4 cell count compared to HIV‐negative individuals (dose–response for review 1) were extracted. We did not include estimates which only compared HPV risk between PLHIV by CD4 levels, without using HIV negative as the comparator. Where possible, and if not otherwise reported, we derived estimates with HIV‐negative individuals as the comparison group.

Studies measured HPV incidence and clearance in different ways, which we defined as follows. For HPV incidence, two situations were possible. An individual's HPV status could change from (1) no HPV DNA present (but not necessarily naïve to past HPV infection, that is, individuals could be seropositive to a given type) to HPV DNA subsequently being present (defined as “first HPV”); or from (2) DNA of one or more HPV types present to DNA of a new and different HPV type subsequently being present (defined as “new HPV”). For clearance of HPV two situations were possible. An individual's HPV status could change from (1) DNA of one or more HPV types present to no HPV DNA subsequently being present (defined as “clearance of all HPV types”); or from (2) DNA of a specific HPV type present to no DNA of that HPV type subsequently being present (defined as “clearance of any HPV type”). In addition, HPV clearance could be defined in the study on the basis of one HPV negative test or two successive negative tests.

The association between HIV and HPV for grouped HPV types ([any]HPV, HR‐HPV or LR‐HPV) could be measured in the study using one of three different units of analysis for HPV: individual‐level, type‐level, or visit‐level. At the individual‐level, only one occurrence for each HPV event was counted per individual during the entire follow‐up period. This meant that, for incident HR‐HPV, for example, an individual could acquire both HPV‐16 and HPV‐18 but this would only count as one event. By definition, clearance of all HPV types could only be measured at the individual‐level. When measured at the type‐level, an individual could have multiple events occurring at different times, for example, acquire multiple HPV types. The studies would count them and the rates would then be averaged over the group of HPV types (HR‐HPV or LR‐HPV) acquired. Note these units are equivalent for the incidence (or alternatively clearance) of one HPV type as only one event is possible. At the visit‐level, which was less commonly used as the unit of analysis across studies, only one event could be counted per individual for each study interval, but events were then summed for all study intervals over the entire follow‐up period.

Eligible studies were examined (KJL) to extract crude RR and adjusted aRR estimates (by sex, where possible) and 95% confidence intervals (95% CI), as well as information on participant characteristics (e.g. geographical region, risk population) and study characteristics (e.g. type of RR, study year), including indicators of study quality such as frequency of visits/testing interval and key variables adjusted for, in a standardised form (comprehensive list in Table 1). A second reviewer checked the data extraction and calculations (MMR). In order of preference, for both reviews RR estimates based on hazard or incidence rate ratio (HRR), cumulative risk ratio (CRR), or odds ratio (OR) were extracted. If multiple estimates of the same association and using the same RR measure were reported by multiple publications reporting on the same study, the estimate corresponding to the largest sample size was extracted. If a publication (or multiple publications from the same study) reported multiple relevant associations (such as the association between HIV status and acquisition of first HPV infection and the association between HIV status and acquisition of new HPV infection), they were all extracted and tabulated although only one independent estimate was included in the pooled estimate of a specific association. Where crude estimates or their 95% CI were not reported, but sufficient information was provided, we calculated (in order of preference) the crude HRR or CRR and 95% CI (details in Appendix S1).

**Table 1:** Summary of characteristics of the 37 independent studies included

Characteristic		Number of independent studies (N = 37)	
		Review 1: Effect of HIV on HPV acquisition and clearance (N = 27 independent studies)	Review 2: Effect of prevalent or incident HPV or HPV clearance on HIV acquisition (N = 11 independent studies)
Sex	Males	7 17, 18, 36, 37, 50, 52, 53, 54, 70	4 12, 55, 56, 57, 64, 65
	Females	18 19, 21, 22, 23, 24, 25, 26, 27, 28, 29, 30, 31, 32, 33, 34, 35, 38, 39, 41, 42, 43, 44, 45, 46, 47, 49, 51, 67, 68	7 12, 43, 58, 59, 60, 61, 62, 63, 66, 69
	Both males and females	2 20, 40, 48	0
Region	North America	10 17, 18, 19, 20, 21, 22, 23, 24, 25, 26, 27, 28, 29, 30, 31, 32, 33, 34, 35, 47, 48, 49	1 55
	South America	3 36, 51, 68	0
	Europe	4 37, 38, 39, 70	0
	Sub‐Saharan Africa	9 40, 41, 42, 43, 44, 45, 46, 50, 54, 67	10 12, 43, 56, 57, 58, 59, 60, 61, 62, 63, 64, 65, 66, 69
	Asia and Pacific	1 52, 53	0
Risk population	General population (including couples studies)	7 40, 41, 43, 44, 50, 54, 67, 68	6 12, 43, 56, 57, 59, 60, 61, 64, 65, 66
	ANC/pregnant	3 42, 46, 51	0
	MSM	5 17, 18, 37, 52, 53, 70	1 55
	PWID	2 19, 24, 25	0
	Gynaecology clinic	1 38	0
	Other higher risk populations	9 20, 21, 22, 23, 26, 27, 28, 29, 30, 31, 32, 33, 34, 35, 36, 39, 45, 47, 48, 49	4 12, 58, 62, 63, 69
Study year (midpoint)	<1989‐1996	9 17, 18, 24, 25, 26, 27, 28, 29, 30, 31, 32, 33, 34, 35, 45, 46, 49	0
	1997‐2006	10 20, 21, 22, 23, 36, 37, 38, 39, 41, 42, 43, 44, 47, 48	10 12, 43, 55, 56, 57, 59, 60, 61, 62, 63, 64, 65, 66, 69
	≥2007	5 51, 52, 53, 67, 68, 70	1 58
	Not reported	3 19, 40, 50, 54	0
Study design	Individual‐based studies	26 17, 18, 19, 20, 21, 22, 23, 24, 25, 26, 27, 28, 29, 30, 31, 32, 33, 34, 35, 36, 37, 38, 39, 41, 42, 43, 44, 45, 46, 47, 48, 49, 50, 51, 52, 53, 54, 67, 68, 70	11 12, 43, 55, 56, 57, 58, 59, 60, 61, 62, 63, 64, 65, 66, 69
	Couple‐based studies	1 40	0
Study type	Longitudinal (cohort or trial)	27 17, 18, 19, 20, 21, 22, 23, 24, 25, 26, 27, 28, 29, 30, 31, 32, 33, 34, 35, 36, 37, 38, 39, 40, 41, 42, 43, 44, 45, 46, 47, 48, 49, 50, 51, 52, 53, 54, 67, 68, 70	8 12, 55, 56, 57, 58, 59, 60, 61, 62, 63, 64
	Case–control with a time element	0	3 12, 43, 65, 66, 69
Follow‐up duration	Range	6 to 53 months 17, 18, 19, 20, 21, 22, 23, 24, 25, 26, 27, 28, 29, 30, 31, 32, 33, 35, 36, 37, 38, 39, 40, 41, 42, 43, 44, 45, 46, 47, 48, 49, 50, 51, 52, 53, 54, 67, 68, 70	12 to 48 months 12, 43, 55, 56, 57, 58, 59, 60, 61, 62, 63, 64, 65, 66, 69
	Unspecified	1 34	0
Length of time between visits	≤6 months	17 18, 19, 21, 22, 23, 24, 25, 26, 27, 28, 29, 30, 31, 32, 33, 34, 35, 36, 37, 38, 40, 42, 43, 47, 49, 51, 67, 70	6 12, 43, 55, 58, 59, 62, 66, 69
	>6 months	3 20, 44, 46, 48	0
	Not regularly spaced	4 17, 45, 50, 52, 53, 54	4 12, 56, 57, 60, 61, 64, 65
	Not reported	3 39, 41, 68	1 63
HPV infection^a^	HPV	13 17, 18, 19, 20, 24, 25, 26, 27, 28, 29, 30, 38, 39, 40, 41, 42, 46, 48, 49, 68	9 12, 43, 56, 57, 58, 59, 60, 61, 62, 64, 65, 66, 69
	HR‐HPV	10 19, 20, 21, 22, 23, 31, 32, 33, 35, 40, 44, 47, 48, 50, 52, 53, 54, 70	9 12, 43, 56, 57, 58, 59, 60, 61, 63, 64, 65, 66, 69
	LR‐HPV	2 31, 32, 33, 40	8 12, 43, 56, 57, 58, 59, 63, 64, 65, 66, 69
	Single HPV vaccine types	8 21, 22, 23, 26, 27, 28, 29, 30, 31, 32, 33, 37, 44, 47, 49, 50, 52, 53, 54, 70	1 12, 59
	Number of HPV types	2 43, 50, 54	6 12, 43, 55, 56, 57, 63, 64, 65, 66
Definition of incident HPV infection (for grouped type)	First	13 17, 18, 19, 20, 21, 22, 23, 26, 27, 28, 29, 30, 38, 39, 40, 41, 46, 47, 48, 49, 68, 70	3 12, 60, 61, 65, 69
	New	10 17, 24, 25, 26, 27, 28, 29, 30, 31, 32, 33, 35, 40, 42, 44, 49, 52, 53, 54	1 12, 43, 66
HPV clearance^b^	HPV	15 17, 18, 20, 24, 25, 26, 27, 28, 29, 30, 34, 36, 38, 39, 40, 41, 42, 45, 46, 48, 49, 51	3 12, 43, 65, 66, 69
	HR‐HPV	10 21, 22, 23, 26, 27, 28, 29, 30, 31, 32, 33, 34, 35, 44, 45, 47, 49, 50, 52, 53, 54, 67, 70	2 12, 59, 60, 61
	LR‐HPV	3 31, 32, 33, 34, 45	1 59
	Single HPV vaccine types	8 21, 22, 23, 24, 25, 31, 32, 33, 44, 45, 47, 50, 52, 53, 54, 70	0
	Number of HPV types	0	1 65
Definition of HPV clearance (for grouped type)	All types	17 17, 18, 20, 21, 22, 23, 24, 25, 26, 27, 28, 29, 30, 34, 35, 36, 38, 39, 41, 45, 46, 47, 48, 49, 51, 52, 53, 67	2 12, 59, 60, 61
	Any	8 26, 27, 28, 29, 30, 31, 32, 33, 40, 42, 49, 50, 52, 53, 54, 67, 70	3 12, 43, 65, 66, 69
Unit of analysis	Individual‐level	25 17, 18, 19, 20, 21, 22, 23, 24, 25, 26, 27, 28, 29, 30, 31, 32, 33, 34, 35, 36, 37, 38, 39, 40, 41, 42, 43, 44, 45, 46, 47, 48, 49, 51, 52, 53, 67, 68	11 12, 43, 55, 56, 57, 58, 59, 60, 61, 62, 63, 64, 66, 69
	Type‐level	5 26, 27, 28, 29, 30, 40, 44, 49, 50, 54, 70	1 59
	Visit‐level	2 24, 25, 50, 54	1 65
Measure of association	HRR	15 17, 19, 20, 21, 22, 23, 24, 25, 26, 27, 28, 29, 30, 31, 32, 33, 35, 37, 40, 44, 45, 47, 48, 49, 50, 52, 53, 54, 70	8 12, 43, 55, 56, 57, 59, 60, 61, 62, 63, 64, 66
	CRR	16 17, 18, 21, 22, 23, 24, 25, 26, 27, 28, 29, 30, 34, 36, 38, 39, 41, 42, 46, 47, 49, 50, 51, 54, 67, 68	6 12, 43, 57, 58, 63, 64, 65, 66, 69
	OR	5 21, 22, 23, 24, 25, 26, 27, 28, 29, 30, 42, 43, 47, 49	3 12, 43, 65, 66, 69
	Crude	26 17, 18, 19, 20, 21, 22, 23, 24, 25, 26, 27, 28, 29, 30, 31, 32, 33, 34, 35, 36, 37, 38, 39, 40, 42, 43, 44, 45, 46, 47, 48, 49, 50, 51, 52, 53, 54, 67, 68, 70	11 12, 43, 55, 56, 57, 58, 59, 60, 61, 62, 63, 64, 65, 66, 69
	Adjusted	16 17, 19, 20, 21, 22, 23, 24, 25, 26, 27, 28, 29, 30, 40, 41, 42, 43, 44, 45, 47, 48, 49, 50, 51, 52, 53, 54, 70	10 12, 43, 55, 56, 57, 59, 60, 61, 62, 63, 64, 65, 66, 69
Stratified by CD4 level	Cutpoints varied across studies	8 17, 21, 22, 23, 26, 27, 28, 29, 30, 34, 35, 40, 47, 49, 70	Not applicable
Key variables adjusted for	HSV‐2	3 19, 20, 26, 27, 28, 29, 30, 48, 49	8 12, 43, 55, 56, 57, 59, 62, 64, 65, 66, 69
	No. of sexual partners	12 17, 19, 20, 24, 25, 26, 27, 28, 29, 30, 41, 43, 44, 45, 48, 49, 50, 52, 53, 54, 70	6 12, 43, 55, 56, 60, 61, 63, 65, 66
	Hormonal contraception	2 20, 45, 48	2 12, 43, 60, 61, 66
	Male circumcision	2 50, 54, 70	4 12, 56, 57, 59, 64, 65
	Condom use	5 17, 19, 20, 45, 48, 52, 53	8 12, 43, 55, 56, 57, 59, 60, 61, 63, 64, 65, 66

ANC, Antenatal clinic attendees; MSM, men who have sex with men; PWID, people who inject drugs; HRR, hazard rate ratio; CRR, cumulative risk ratio; OR, odds ratio.

a Defined for incident HPV as first (among those with no HPV DNA present at baseline but not necessarily naïve to past HPV infection) or new (among individuals who are already had HPV DNA of another type at baseline) HPV group type not present at baseline.

b Defined as clearance of all HPV types or clearance of any one type or an average of type‐specific clearance rates. The unit of analysis was entered as “individual‐level” for the association between HIV and acquisition/clearance for single HPV types as this is equivalent to “type‐level” being the unit of infection for single HPV types, unless “type‐level” was the unit of analysis for other estimates. Studies are counted more than once where they presented estimates for more than one association, unit of analysis or measure of association, or adjusted for more than one confounding factor. Measure of association refers to the measure entered in Tables S1 and S2. One study was counted in both reviews.

### 2.3 Meta‐analysis

For each association, forest plots and pooled estimates were used to summarize independent RR estimates across studies. Pooled natural log RR estimates and 95% CI of log transformed study RR estimates were derived using a random‐effects model based on the inverse‐variance method 16. For each association, one RR estimate per study for each sex was included according to the following algorithm: we preferably used estimates (1) based on the female sample, if female and male estimates were non‐independent (such as in couples studies); (2) based on new HPV over first HPV, and on clearance of all HPV types over clearance of any HPV type, if both were reported in a study; (3) using individuals as unit of analysis, if a study reported multiple estimates based on different unit of analysis; and (4) based on penile samples over anal samples, if both were reported in a study. The influence of (1), (2), (3) and (4) was explored in subgroup analyses. For review 2, we also derived additional pooled estimates with alternative comparison groups to HPV‐negative (e.g. prevalent HR‐HPV *vs*. HR‐HPV‐negative) to compensate for the few available estimates using HPV‐negative as the comparison. Estimates without 95% CI or which were numerically undefined (e.g. due to no unexposed cases) were excluded from the meta‐analysis. Statistical heterogeneity across study estimates was assessed with the *I* ^2^ statistic. Subgroup analyses were used to explore the influence of participant and study characteristics and quality for the associations with HPV and HR‐HPV. The meta‐analyses were done in Stata (version 14) and forest plots produced in R.

### 2.4 Study quality and publication bias

Information on study quality from the data extraction, such as definitions of HPV incidence and clearance and unit of analysis, was assessed quantitatively using subgroup analyses by relevant study characteristics. In particular, we assessed potential for confounding, for example, in adjusted estimates using subgroup analysis by whether or not a key confounder (HSV‐2, number of sexual partners, hormonal contraception, male circumcision, condom use) was adjusted for. We also compared pooled crude and adjusted estimates for the subset of studies which reported both types of estimates. Publication bias was assessed in three ways: first with funnel plots of crude study estimates as described in Appendix S1, and second with subgroup analysis comparing pooled crude RR from estimates directly reported in publications with estimates not reported but which were derived from available information. This assumes that in the presence of publication bias, the latter would be smaller than estimates directly reported in publications. Third, during the data extraction we identified situations where an association had been investigated, but where results were selectively reported, for example, according to statistical significance. This information was then evaluated qualitatively.

## 3 Results and discussion

### 3.1 Study selection

**Figure 1:**
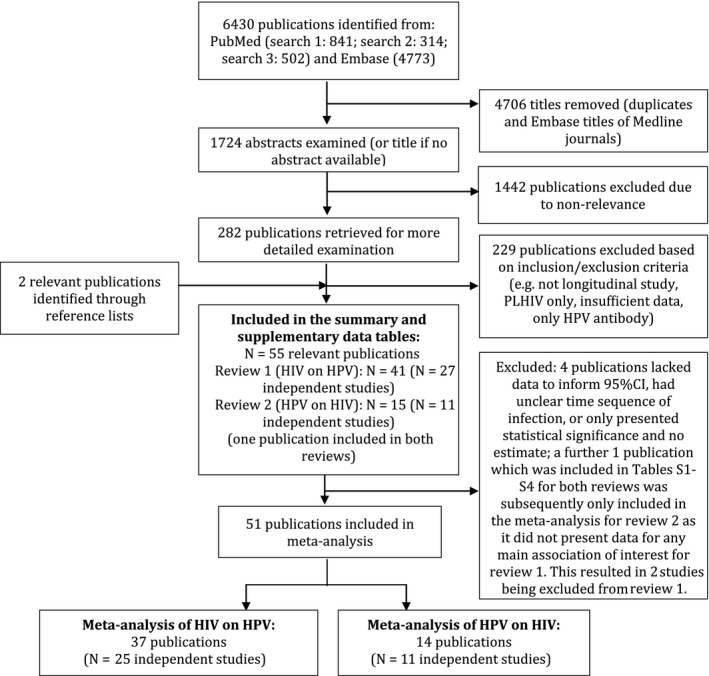
Results and selection of publications from the literature search.

Of the 6430 potential publications identified from the PubMed and Embase searches, 55^12^, ^17^, ^18^, ^19^, ^20^, ^21^, ^22^, ^23^, ^24^, ^25^, ^26^, ^27^, ^28^, ^29^, ^30^, ^31^, ^32^, ^33^, ^34^, ^35^, ^36^, ^37^, ^38^, ^39^, ^40^, ^41^, ^42^, ^43^, ^44^, ^45^, ^46^, ^47^, ^48^, ^49^, ^50^, ^51^, ^52^, ^53^, ^54^, ^55^, ^56^, ^57^, ^58^, ^59^, ^60^, ^61^, ^62^, ^63^, ^64^, ^65^, ^66^, ^67^, ^68^, ^69^, ^70^ met our inclusion criteria. Figure 1 summarizes publication selection, Table 1 summarizes the characteristics of the included studies, and Tables S1,S2,S3,S4 (Appendix S1) describe the extracted data and their quality. In review 1*,* of the effect of HIV status (exposure) on subsequent HPV acquisition and clearance (outcomes), we extracted data from a total of 41 publications reporting on 27 independent studies 17, 18, 19, 20, 21, 22, 23, 24, 25, 26, 27, 28, 29, 30, 31, 32, 33, 34, 35, 36, 37, 38, 39, 40, 41, 42, 43, 44, 45, 46, 47, 48, 49, 50, 51, 52, 53, 54. In review 2, of the effect of HPV status (exposure) on subsequent HIV acquisition (outcome), we extracted data from a total of 15 publications reporting results from 11 independent studies 12, 43, 55, 56, 57, 58, 59, 60, 61, 62, 63, 64, 65, 66. This included one published review that provided additional unpublished estimates from other studies 12. One publication 43 was included in both reviews. Four of the 55 publications included did not report RR and/or 95% CI estimates or sufficient data to derive them, or had imprecise information on the time sequence of infection, and could not be included in the meta‐analysis 22, 29, 37, 60. The publication 43 from which data were extracted for both reviews did not present any data for our main associations of interest for review 1 and was therefore not included in any meta‐analysis for review 1. This left 37 publications (25 independent studies) for review 1 and 14 publications (11 independent studies) for review 2 in the meta‐analyses. Two studies in review 2 were new additions to the previously published meta‐analyses 12, 13.

### 3.2 Study and participant characteristics

In review 1, 10 of the 27 studies were conducted in North America and 9 in Sub‐Saharan Africa (Table 1). Eighteen were conducted among females only, and 9 were carried out before 1997 and 5 after 2006. Ten studies were conducted among lower risk populations (participants who were recruited from the general population and antenatal clinic attendees), while the remaining 17 studies were among higher risk populations (e.g. studies which included men who have sex with men (MSM), people who inject drugs (PWID) and female sex workers (FSW)).

In review 1, studies reported on the association between HIV and incident HPV infection for infection with HPV (N = 13), HR‐HPV (N = 10), LR‐HPV (N = 2), single HPV nonavalent vaccine type (N = 8) and by number of HPV types acquired (N = 2). Studies reported RR estimates of HPV clearance by HIV status for HPV (N = 15), HR‐HPV (N = 10), LR‐HPV (N = 3) and single HPV nonavalent vaccine type (N = 8). Eight studies compared HPV incidence or HPV clearance outcomes between HIV‐negative and PLHIV stratified by CD4 level (dose–response), six of which reported results of a statistical test for trend by CD4 level (Table S1; see Appendix S1).

In review 2, 10 of the 11 studies were conducted in Sub‐Saharan Africa (one study was from North America), all after 1996 with 1 after 2006, and 7 among females (Table 1). Participants were recruited from general (N = 6) and higher risk populations (N = 4) and one from MSM. Studies reported RR estimates for the association between incident HIV and preceding exposure to prevalent HPV (N = 9), prevalent HR‐HPV (N = 9), prevalent LR‐HPV (N = 8), single HPV nonavalent vaccine type (N = 1), and by number of HPV types present (dose–response) (N = 6). Few studies reported RR estimates of incident HIV following HPV incidence for first HPV (N = 3) and for new HPV (N = 1), and following HPV clearance for HPV irrespective of type (N = 3), for HR‐HPV (N = 2), and by number of HPV types (dose–response) (N = 1).

### 3.3 Meta‐analysis results for review 1

#### 3.3.1 HPV acquisition by HIV status

**Figure 2:**
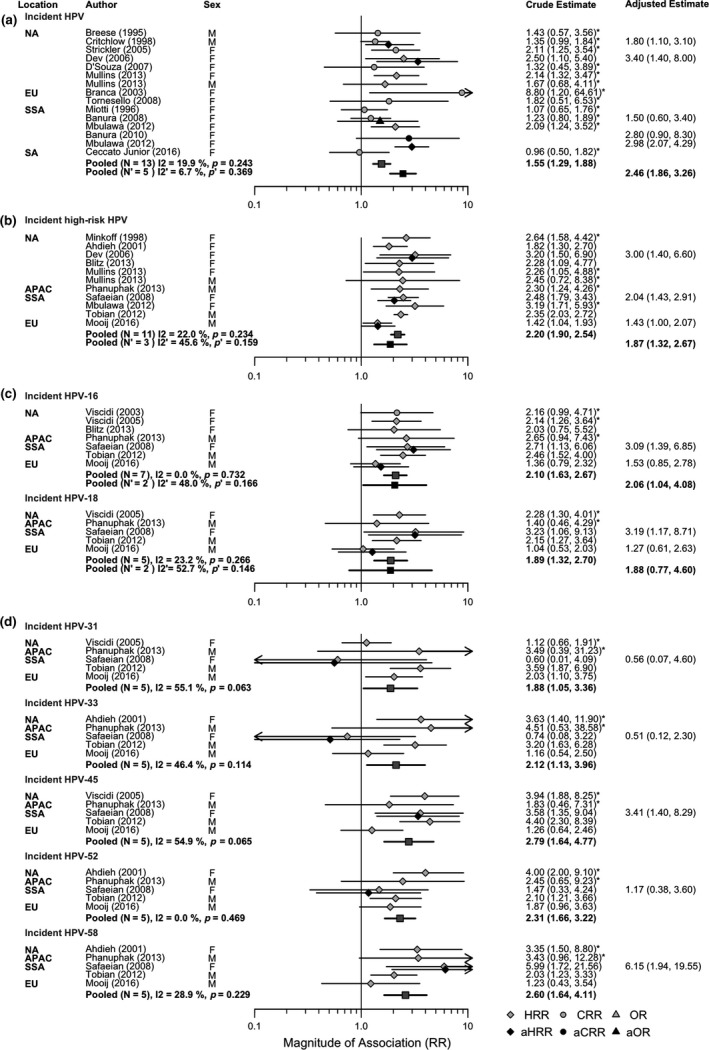
Forest plots for the crude and adjusted relative risk (RR) of: **(a)** incident HPV infection; **(b)** incident HR‐HPV infection; **(c)** incident HPV‐16/HPV‐18 infection; and **(d)** incident HPV‐31/HPV‐33/HPV‐45/HPV‐52/HPV‐58 infection, by HIV status. In this plot all HIV infection is prevalent, and the comparison group (unexposed group) is those HIV‐negative. An effect estimate greater than 1 indicates increased HPV incidence in those with HIV infection compared to HIV‐negative individuals. An asterisk next to the effect estimate indicates that this estimate was calculated using data presented in the publication. NA, North America; EU, Europe; SSA, Sub‐Saharan Africa; SA, South America; APAC, Asia and Pacific.

Both the pooled crude RR and adjusted aRR suggested a statistically significant increased risk of acquisition of HPV (pooled RR = 1.55, 95% CI: 1.29 to 1.88; pooled aRR = 2.46, 95% CI: 1.86 to 3.26), HR‐HPV (pooled RR = 2.20, 95% CI: 1.90 to 2.54; pooled aRR = 1.87, 95% CI: 1.32 to 2.67) and HPV‐16 (pooled RR 2.10, 95% CI: 1.63 to 2.67; pooled aRR = 2.06, 95% CI: 1.04 to 4.08), and slightly more equivocal increased risk of HPV‐18 (pooled RR = 1.89, 95% CI: 1.32 to 2.70; pooled aRR = 1.88, 95% CI: 0.77 to 4.60), in PLHIV compared to HIV‐negative individuals (Figure 2a,b,c). Incident HPV‐31, HPV‐33, HPV‐45, HPV‐52 and HPV‐58 (range of pooled RR: 1.88‐2.79) were also positively and statistically significantly associated with HIV status in crude pooled RR. The only adjusted estimates (N = 1) were statistically significant for HPV‐45 and HPV‐58 (Figure 2d). Incident LR‐HPV was positively and statistically significantly associated with HIV status in crude pooled RR (pooled RR = 2.62, 95% CI: 2.04 to 3.36, N = 2); no adjusted estimates were available (Figure S1a,b; see Appendix S1). Pooled adjusted aRR tended to have wider confidence intervals than RR because adjusted estimates were less common. Statistical heterogeneity across crude and adjusted study estimates for each HPV outcome varied from 0% to 55%.

In subgroup analyses by participant and study characteristics, the magnitude of the stratified pooled crude RR and adjusted aRR for incidence of HPV and HR‐HPV by HIV status were similar to unstratified pooled estimates (e.g. HIV on HPV incidence among heterosexual males: pooled RR = 1.95, 95% CI: 1.62, 2.34, N = 2; females: pooled RR = 1.63, 95% CI: 1.26 to 2.11, N = 10; MSM: pooled RR = 1.36, 95% CI: 1.01 to 1.82, N = 2), and remained statistically significant overall, with the exception of pooled RR for subgroups based on few study estimates, which were not always statistically significantly different from the null (Table 2a,b).

**Table 2:** Subgroup analyses of the association between HIV exposure status and subsequent HPV infection by participant and study characteristics (review 1). Pooled crude RR and adjusted aRR are presented for the following HPV outcomes: (a) incident HPV; (b) incident HR‐HPV; (c) clearance of HPV; (d) clearance of HR‐HPV. Estimates in bold are statistically significantly different to 1 (*p* < 0.05)

Study and participant characteristics	Crude pooled				Adjusted pooled			
	N	RR (95% CI)	I^2^ (%)	*p* value^f^ (X^2^)	N	aRR (95% CI)	I^2^ (%)	*p* value^f^ (X^2^)
**a. Incident HPV**								
Sex								
Males^c^	4	**1.42 (1.10, 1.82)**	0.0	0.959	2	**1.95 (1.51, 2.52)**	0.0	0.729
Females	10	**1.63 (1.26, 2.11)**	36.2	0.119	4	**2.77 (2.05, 3.75)**	0.0	0.515
Region								
North America	7	**1.67 (1.36, 2.05)**	0.0	0.563	2	**2.24 (1.24, 4.04)**	33.9	0.219
South America	1	0.96 (0.51, 1.82)	‐	‐	‐	‐	‐	‐
Europe	2	3.30 (0.74, 14.74)	41.2	0.192	‐	‐	‐	‐
Sub‐Saharan Africa	3	1.38 (0.94, 2.02)	46.4	0.155	3	**2.68 (1.92, 3.75)**	2.5	0.359
Economy^d^								
Low‐ and middle‐income economies	4	1.29 (0.93, 1.78)	36.2	0.195	3	**2.68 (1.92, 3.75)**	0.0	0.359
High‐income economies	9	**1.70 (1.39, 2.09)**	0.0	0.483	2	**2.24 (1.24, 4.04)**	33.9	0.219
Risk population^e^								
MSM only (any site)	2	**1.36 (1.01, 1.82)**	0.0	0.912	1	**1.80 (1.07, 3.02)**	‐	‐
MSM, penile samples alone	‐	**‐**	‐	‐	‐	**‐**	‐	‐
MSM, anal samples alone	1	1.35 (0.99, 1.84)	‐	‐	1	**1.80 (1.07, 3.02)**	‐	‐
Heterosexual men only	2	**1.95 (1.62, 2.34)**	0.0	0.734	1	**2.00 (1.49, 2.53)**	‐	‐
Higher risk populations	9	**1.70 (1.39, 2.09)**	0.0	0.483	2	**2.24 (1.24, 4.04)**	33.9	0.219
Lower risk populations	4	1.29 (0.93, 1.78)	36.2	0.195	3	**2.68 (1.92, 3.75)**	2.5	0.359
Study year (midpoint)								
<1997	5	**1.40 (1.12, 1.75)**	0.0	0.467	1	**1.80 (1.07, 3.02)**	‐	‐
1997 to 2006	6	**1.82 (1.29, 2.56)**	24.2	0.252	3	**2.37 (1.39, 4.06)**	0.0	0.404
≥2007	2	1.45 (0.68, 3.10)	‐	‐	1	**2.98 (2.07, 4.29)**	‐	‐
Incidence definition								
First incident HPV infection^a^	11	**1.56 (1.25, 1.94)**	20.1	0.252	3	**2.21 (1.46, 3.34)**	0.0	0.424
New incident HPV infection^b^	4	**1.80 (1.13, 2.87)**	85.4	<0.001	3	**2.49 (1.86, 3.34)**	15.9	0.304
Measure of association								
ALL	13	**1.55 (1.29, 1.88)**	19.9	0.243	5	**2.46 (1.86, 3.26)**	6.7	0.369
HRR	4	**2.11 (1.56, 2.86)**	0.0	0.932	3	**2.57 (1.79, 3.69)**	29.5	0.242
CRR	9	**1.36 (1.11, 1.66)**	6.6	0.380	1	2.80 (0.92, 8.50)	‐	‐
OR	‐	**‐**	‐	‐	1	1.50 (0.63, 3.57)	‐	‐
Unit of analysis								
Individual‐level	13	**1.55 (1.29, 1.88)**	19.9	0.243	4	**2.06 (1.42, 2.99)**	0.0	0.505
Type‐level	‐	‐	‐	‐	1	**2.98 (2.07, 4.29)**	‐	‐
Key variables adjusted for								
HSV‐2								
Yes	‐	‐	‐	‐	1	**3.40 (1.42, 8.13)**	‐	‐
No	‐	‐	‐	‐	4	**2.33 (1.67, 3.24)**	19.8	0.291
Number of sexual partners								
Yes	‐	‐	‐	‐	3	**2.21 (1.46, 3.34)**	0.0	0.424
No	‐	‐	‐	‐	2	**2.38 (1.26, 4.57)**	51.1	0.153
Hormonal contraception								
Yes	‐	‐	‐	‐	‐	‐	‐	‐
No	‐	‐	‐	‐	5	**2.46 (1.86, 3.26)**	6.7	0.369
Male circumcision								
Yes	‐	‐	‐	‐	‐	‐	‐	‐
No	‐	‐	‐	‐	5	**2.46 (1.86, 3.26)**	6.7	0.369
Condom use								
Yes	‐	‐	‐	‐	2	**2.24 (1.24, 4.04)**	33.9	0.219
No	‐	‐	‐	‐	3	**2.68 (1.92, 3.75)**	2.5	0.359
**Comparing subset with crude and adjusted**	4	**1.54 (1.16, 2.05)**	31.8	0.221	4	**2.38 (1.68, 3.38)**	29.2	0.237
**Risk of publication bias**								
Estimate reported in study	1	**2.50 (1.13, 5.54)**	‐	‐	5	**2.46 (1.86, 3.26)**	6.7	0.369
Estimate derived from study information	12	**1.51 (1.25, 1.83)**	18.2	0.321	‐	**‐**	‐	‐
**b. Incident HR‐HPV**								
Sex								
Males	4	**1.90 (1.35, 2.67)**	34.4	0.206	1	1.43 (0.99, 2.06)	‐	‐
Females	7	**2.37 (1.97, 2.87)**	0.0	0.718	2	**2.18 (1.58, 3.01)**	0.0	0.375
Region								
North America	6	**2.22 (1.74, 2.83)**	0.0	0.784	1	**3.00 (1.38, 6.51)**	‐	‐
Europe	1	**1.42 (1.04, 1.93)**	‐	‐	1	1.43 (0.99, 2.06)	‐	‐
Sub‐Saharan Africa	3	**2.40 (2.11, 2.74)**	0.0	0.630	1	**2.04 (1.43, 2.91)**	‐	‐
Asia and Pacific	1	**2.30 (1.24, 4.26)**	‐	‐	‐	‐	‐	‐
Economy^d^								
Low‐ and middle‐income economies	4	**2.40 (2.10, 2.73)**	0.0	0.815	1	**2.04 (1.43, 2.91)**	‐	‐
High‐income economies	7	**1.95 (1.55, 2.45)**	18.8	0.286	2	1.91 (0.94, 3.87)	‐	‐
Risk population^e^								
MSM only (any site)	2	**1.67 (1.07, 2.62)**	46.8	0.170	1	1.43 (0.99, 2.06)	‐	‐
MSM, penile samples alone	1	**1.42 (1.04, 1.93)**	‐	‐	1	1.43 (0.99, 2.06)	‐	‐
MSM, anal samples alone^g^	2	**1.82 (1.47, 2.25)**	0.0	0.424	1	**1.63 (1.29, 2.06)**	‐	‐
Heterosexual men only	2	**2.49 (1.54, 4.04)**	0.0	0.976	‐	‐	‐	‐
Higher risk populations	7	**1.82 (1.50, 2.21)**	0.3	0.421	2	1.91 (0.94, 3.87)	65.2	0.090
Lower risk populations	4	**2.42 (2.13, 2.75)**	0.0	0.791	1	**2.04 (1.43, 2.91)**	‐	‐
Study year (midpoint)								
<1997	2	**2.10 (1.47, 2.99)**	24.3	0.251	‐	‐	‐	‐
1997 to 2006	5	**2.50 (1.94, 3.23)**	0.0	0.970	2	**2.18 (1.58, 3.01)**	0.0	0.375
≥2007	4	**2.12 (1.53, 2.95)**	69.5	0.020	1	1.43 (0.99, 2.06)	‐	‐
Incidence definition								
First incident HPV infection^a^	6	**2.19 (1.55, 3.10)**	41.0	0.132	2	1.91 (0.94, 3.87)	65.2	0.090
New incident HPV infection^b^	5	**2.32 (2.05, 2.61)**	0.0	0.721	1	**2.48 (1.79, 3.43)**	‐	‐
Measure of association								
ALL	11	**2.20 (1.90, 2.54)**	22.0	0. 234	3	**1.87 (1.32, 2.67)**	45.6	0.159
HRR	11	**2.20 (1.90, 2.54)**	22.0	0. 234	3	**1.87 (1.32, 2.67)**	45.6	0.159
Unit of analysis								
Individual‐level	9	**2.37 (1.98, 2.83)**	0.0	0.883	2	**2.18 (1.58, 3.01)**	0.0	0.375
Type‐level	2	**1.86 (1.14, 3.05)**	88.0	0.004	1	1.43 (0.99, 2.06)	‐	‐
Key variables adjusted for								
HSV‐2								
Yes	‐	‐	‐	‐	1	**3.00 (1.38, 6.51)**	‐	‐
No	‐	‐	‐	‐	2	**1.71 (1.21, 2.43)**	46.7	0.171
Number of sexual partners								
Yes	‐	‐	‐	‐	3	**1.87 (1.32, 2.67)**	45.6	0.159
No	‐	‐	‐	‐	‐	‐	‐	‐
Hormonal contraception								
Yes	‐	‐	‐	‐	‐	‐	‐	‐
No	‐	‐	‐	‐	3	**1.87 (1.32, 2.67)**	45.6	0.159
Male circumcision								
Yes	‐	‐	‐	‐	1	1.43 (0.99, 2.06)	‐	‐
No	‐	‐	‐	‐	2	**2.18 (1.58, 3.01)**	0.0	0.375
Condom use								
Yes	‐	‐	‐	‐	1	**3.00 (1.38, 6.51)**	‐	‐
No	‐	‐	‐	‐	2	**1.71 (1.21, 2.43)**	46.7	0.171
**Comparing subset with crude and adjusted**	3	**2.10 (1.31, 3.37)**	74.2	0.021	3	**1.87 (1.32, 2.67)**	45.6	0.159
**Risk of publication bias**								
Estimate reported in study	6	**2.09 (1.69, 2.59)**	54.0	0.054	2	**2.18 (1.58, 3.01)**	0.0	0.375
Estimate derived from study information	5	**2.60 (1.93, 3.49)**	0.0	0.950	‐	**‐**	‐	‐
**c. Clearance of HPV**								
Sex								
Males^c^	3	**0.74 (0.62, 0.87)**	0.0	0.800	2	**0.54 (0.31, 0.95)**	85.1	0.010
Females	10	**0.52 (0.40, 0.68)**	80.2	<0.001	7	**0.52 (0.38, 0.71)**	67.7	0.005
Region								
North America	4	**0.58 (0.47, 0.72)**	28.4	0.242	3	**0.54 (0.35, 0.82)**	65.3	0.056
South America	2	0.89 (0.57, 1.41)	34.2	0.218	1	1.00 (0.59, 1.68)	‐	‐
Europe	2	**0.38 (0.21, 0.68)**	0.0	0.487	‐	‐	‐	‐
Sub‐Saharan Africa	4	**0.45 (0.29, 0.69)**	87.0	<0.001	4	**0.40 (0.32, 0.51)**	13.4	0.325
Economy^d^								
Low‐ and middle‐income economies	6	**0.54 (0.36, 0.80)**	87.0	<0.001	5	**0.48 (0.32, 0.71)**	70.2	0.009
High‐income economies	6	**0.55 (0.44, 0.69)**	27.6	0.228	3	**0.54 (0.35, 0.82)**	65.3	0.056
Risk population^e^								
MSM only (any site)	1	0.62 (0.29, 1.32)	‐	‐	1	**0.40 (0.28, 0.57)**	‐	‐
MSM, penile samples alone	‐	**‐**	‐	‐	‐	**‐**	‐	‐
MSM, anal samples alone	‐	**‐**	‐	‐	1	**0.40 (0.28, 0.57)**	‐	‐
Heterosexual men only	2	**0.74 (0.63, 0.88)**	0.0	0.631	1	**0.71 (0.55, 0.92)**	‐	‐
Higher risk populations	8	**0.48 (0.36, 0.64)**	69.0	0.002	4	**0.46 (0.31, 0.70)**	74.7	0.008
Lower risk populations	4	**0.62 (0.41, 0.94)**	83.9	<0.001	4	**0.55 (0.36, 0.84)**	61.3	0.051
Study year (midpoint)								
<1997	6	**0.46 (0.32, 0.65)**	78.7	<0.001	3	**0.37 (0.29, 0.48)**	1.7	0.362
1997 to 2006	4	**0.59 (0.40, 0.87)**	41.4	0.163	3	**0.57 (0.38, 0.86)**	38.3	0.198
≥2007	2	0.71 (0.35, 1.46)	91.5	0.001	2	0.66 (0.31, 1.41)	84.4	0.011
Clearance definition								
Loss of detection of all HPV types	10	**0.51 (0.38, 0.69)**	77.2	<0.001	6	**0.51 (0.34, 0.75)**	75.0	0.001
Loss of detection of any HPV type	2	**0.62 (0.41 0.94)**	82.1	0.018	2	**0.47 (0.36, 0.61)**	0.0	0.794
Test definition of clearance								
1 negative test	11	**0.58 (0.48, 0.72)**	61.9	0.003	6	**0.51 (0.38, 0.67)**	47.9	0.087
2 consecutive negative tests	1	**0.30 (0.22, 0.41)**	‐	‐	2	0.48 (0.21, 1.09)	90.6	0.001
Measure of association								
ALL	12	**0.53 (0.42, 0.67)**	75.9	<0.001	8	**0.50 (0.38, 0.66)**	65.5	0.005
HRR	2	**0.39 (0.24, 0.65)**	85.9	0.008	4	**0.45 (0.33, 0.63)**	74.4	0.008
CRR	10	**0.60 (0.48, 0.75)**	60.1	0.007	2	0.60 (0.19, 1.92)	75.7	0.042
OR	‐	**‐**	‐	‐	2	**0.51 (0.33, 0.79)**	0.0	0.898
Unit of analysis								
Individual‐level	10	**0.51 (0.38, 0.69)**	77.2	<0.001	6	**0.51 (0.34, 0.75)**	75.0	0.001
Type‐level	2	**0.62 (0.41 0.94)**	82.1	0.018	2	**0.47 (0.36, 0.61)**	0.0	0.794
Key variables adjusted for								
HSV‐2								
Yes	‐	‐	‐	‐	2	**0.68 (0.50, 0.92)**	0.0	0.437
No	‐	‐	‐	‐	6	**0.46 (0.34, 0.63)**	64.0	0.016
Number of sexual partners								
Yes	‐	‐	‐	‐	5	**0.45 (0.31, 0.65)**	68.1	0.014
No	‐	‐	‐	‐	3	**0.56 (0.37, 0.96)**	69.4	0.038
Hormonal contraception								
Yes	‐	‐	‐	‐	2	0.48 (0.21, 1.09)	90.6	0.001
No	‐	‐	‐	‐	6	**0.51 (0.38, 0.67)**	47.9	0.087
Male circumcision								
Yes	‐	‐	‐	‐	‐	‐	‐	‐
No	‐	‐	‐	‐	8	**0.50 (0.38, 0.66)**	65.5	0.005
Condom use								
Yes	‐	‐	‐	‐	3	**0.45 (0.28, 0.74)**	83.0	0.003
No	‐	‐	‐	‐	5	**0.55 (0.39, 0.77)**	48.4	0.101
** Comparing subset with crude and adjusted**	5	**0.57 (0.37, 0.89)**	88.5	<0.001	5	**0.51 (0.35, 0.73)**	68.6	0.012
**d. Clearance of HR‐HPV**								
Sex								
Males	3	0.65 (0.34, 1.25)	93.5	0.000	3	0.52 (0.19, 1.42)	93.2	<0.001
Females	7	**0.68 (0.61, 0.76)**	13.8	0.324	1	**0.71 (0.58, 0.91)**	‐	‐
Region								
North America	5	**0.65 (0.53, 0.79)**	39.8	0.156	‐	‐	‐	‐
Europe	1	**1.33 (1.02, 1.73)**	‐	‐	1	1.28 (0.96, 1.71)	‐	‐
Sub‐Saharan Africa	3	**0.68 (0.61, 0.76)**	0.0	0.876	2	**0.58 (0.37, 0.92)**	70.9	0.064
Asia and Pacific	1	**0.24 (0.12, 0.49)**	‐	‐	1	**0.22 (0.11, 0.46)**	‐	‐
Economy^d^								
Low‐ and middle‐income economies	4	**0.64 (0.51, 0.80)**	65.0	0.036	3	**0.44 (0.24, 0.81**)	81.8	0.004
High‐income economies	6	0.74 (0.54, 1.00)	82.1	<0.001	1	1.28 (0.96, 1.71)	‐	‐
Risk population^e^								
MSM only (any site)	2	0.58 (0.11, 3.12)	95.0	<0.001	2	0.55 (0.10, 3.08)	94.9	<0.001
MSM, penile samples alone	1	**1.33 (1.02, 1.73)**	‐	‐	1	1.28 (0.96, 1.71)	‐	‐
MSM, anal samples alone^g^	2	0.43 (0.16, 1. 15)	86.8	0.006	2	0.42 (0.13, 1.34)	89.4	0.002
Heterosexual men only	1	**0.67 (0.59, 0.76)**	‐	‐	1	0.44 (0.28, 0.69)	‐	‐
Higher risk populations	6	**0.63 (0.43, 0.91)**	86.7	<0.001	2	0.55 (0.10, 3.08)	94.9	<0.001
Lower risk populations	4	**0.69 (0.62, 0.76)**	0.0	0.844	2	**0.58 (0.37, 0.92)**	70.9	0.064
Study year (midpoint)								
<1997	3	**0.69 (0.59, 0.81)**	14.3	0.311	‐	**‐**	‐	‐
1997 to 2006	3	**0.59 (0.41, 0.85)**	47.2	0.151	1	**0.71 (0.58, 0.91)**	‐	‐
≥2007	4	0.69 (0.45, 1.06)	90.3	<0.001	3	0.52 (0.19, 1.42)	93.2	<0.001
Clearance definition								
Loss of detection of all HR‐HPV types	6	**0.60 (0.46, 0.79)**	64.9	0.014	1	**0.22 (0.11, 0.46)**	‐	‐
Loss of detection of any HR‐HPV type	4	0.78 (0.57, 1.05)	87.6	<0.001	3	0.75 (0.44, 1.30)	88.9	<0.001
Test definition of clearance								
1 negative test	8	**0.65 (0.56, 0.76)**	51.1	0.046	3	**0.44 (0.24, 0.81)**	81.8	0.004
2 consecutive negative tests	2	0.89 (0.41, 1.94)	94.8	<0.001	1	1.28 (0.96, 1.71)	‐	‐
Measure of association								
ALL	10	**0.69 (0.57, 0.83)**	77.0	<0.001	4	0.59 (0.33, 1.05)	90.0	<0.001
HRR	7	**0.65 (0.49, 0.86)**	84.6	<0.001	3	0.64 (0.32, 1.28)	91.3	<0.001
CRR	3	**0.73 (0.63, 0.84)**	0.0	0.985	1	**0.44 (0.28, 0.69)**	‐	‐
Unit of analysis								
Individual‐level	6	**0.60 (0.46, 0.79)**	64.9	0.014	2	**0.33 (0.17, 0.65)**	60.8	0.110
Type‐level	4	0.78 (0.57, 1.05)	87.6	<0.001	2	0.95 (0.53, 1.69)	89.3	0.002
Key variables adjusted for								
HSV‐2								
Yes	‐	‐	‐	‐	‐	‐	‐	‐
No	‐	‐	‐	‐	4	0.59 (0.33, 1.05)	90.0	<0.001
Number of sexual partners								
Yes	‐	‐	‐	‐	4	0.59 (0.33, 1.05)	90.0	<0.001
No	‐	‐	‐	‐	‐	‐	‐	‐
Hormonal contraception								
Yes	‐	‐	‐	‐	‐	‐	‐	‐
No	‐	‐	‐	‐	4	0.59 (0.33, 1.05)	90.0	<0.001
Male circumcision								
Yes	‐	‐	‐	‐	2	0.76 (0.27, 2.2)	93.6	<0.001
No	‐	‐	‐	‐	2	0.42 (0.12, 1.31)	88.9	0.003
Condom use								
Yes	‐	‐	‐	‐	1	**0.22 (0.11, 0.46)**	‐	‐
No	‐	‐	‐	‐	3	0.75 (0.44, 1.30)	88.9	<0.001
Comparing subset with crude and adjusted	3	0.66 (0.32, 1.34)	92.1	<0.001	3	0.64 (0.32, 1.28)	91.3	<0.001

a Defined as the subsequent detection of HPV DNA in those with no HPV DNA present at baseline but not necessarily naïve to past HPV infection.

b Defined as the subsequent detection of HPV DNA among individuals who already had HPV DNA of another type present at baseline.

c Male category includes an estimate from Mbulawa (2012), which cannot be included in the main analyses as the estimate comes from a couples study and male estimate is not independent from female estimate (included in the main analysis as per protocol).

d World Bank definition.

e MSM only: studies which only included men who have sex with men; Higher risk populations: studies which included female sex workers (FSWs), men who have sex with men (MSM), people who inject drugs (PWID) or STI clinic attendees, or studies consisting of participants reporting higher risk sex practices; Lower risk populations are participants from couples studies, antenatal care (ANC) clinics, or other general population samples.

f Relates to I^2^.

g Estimate from Mooij (2016) for anal sampling added in; estimate for penile sampling already included in main analysis as per protocol.

#### 3.3.2 HPV clearance by HIV status

**Figure 3:**
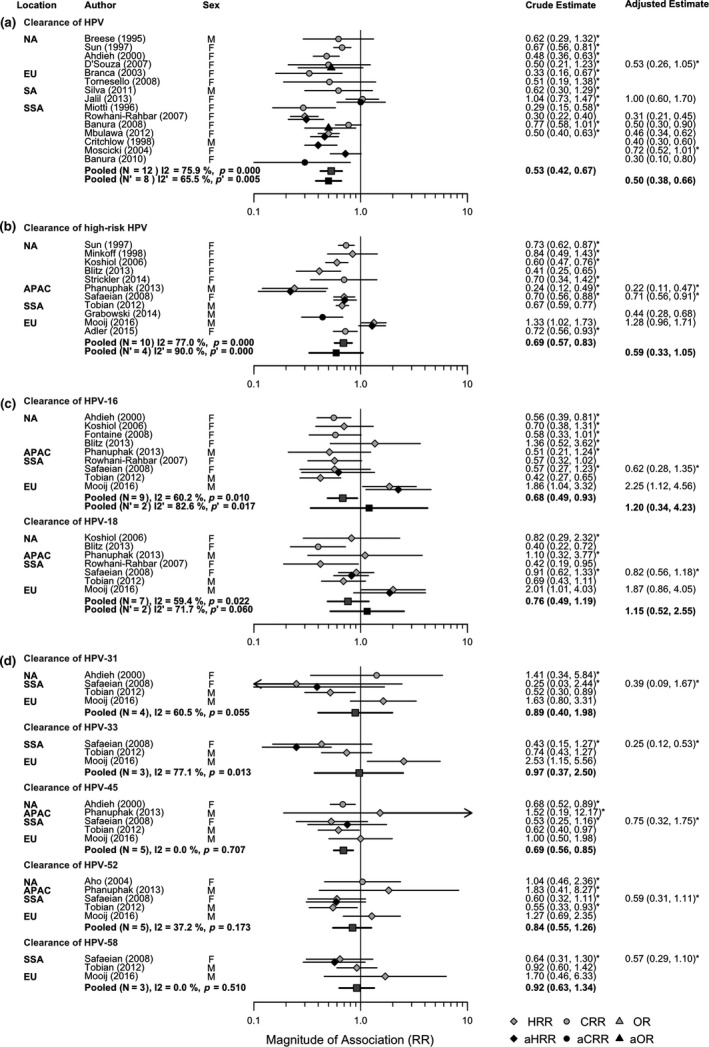
Forest plots for the crude and adjusted relative risk (RR) of: **(a)** clearance of HPV;** (b)** clearance of HR‐HPV;** (c)** clearance of HPV‐16/HPV‐18; and **(d)** clearance of HPV‐31/HPV‐33/HPV‐45/HPV‐52/HPV‐58, by HIV status. In this plot all HIV infection is prevalent, and the comparison group (unexposed group) is those HIV‐negative. An effect estimate <1 indicates decreased rate of HPV clearance in those with HIV infection compared to HIV‐negative individuals. An asterisk next to the effect estimate indicates that this estimate was calculated using data presented in the publication. NA, North America; EU, Europe; SA, South America; SSA, Sub‐Saharan Africa; APAC, Asia and Pacific.

Both pooled crude RR and adjusted aRR suggested that the clearance rate of HPV infection was approximately halved among PLHIV compared to HIV‐negative individuals (pooled RR = 0.53, 95% CI: 0.42 to 0.67; pooled aRR = 0.50, 95% CI: 0.38 to 0.66) and similarly for HR‐HPV (pooled RR = 0.69, 95% CI: 0.57 to 0.83; pooled aRR = 0.59, 95% CI: 0.33 to 1.05) (Figure 3a,b). Associations between HIV and clearance of single type HPV had wide confidence intervals, which were generally not statistically significantly different from the null value (Figure 3c,d). The association between HIV status and LR‐HPV was not statistically significant (pooled RR = 0.77, 95% CI: 0.53 to 1.11, N = 2; Figure S1c; see Appendix S1). Statistical heterogeneity across HPV outcomes ranged from 0% to 90%.

In subgroup analyses, stratified pooled crude RR and adjusted aRR for clearance of HPV and HR‐HPV by HIV status were similar in magnitude overall to unstratified pooled estimates, and remained statistically significant, with the exception of pooled RR for subgroups based on few estimates only, which were not all statistically significantly different from the null (Table 2c,d).

#### 3.3.3 Influence of CD4 cell count on HPV acquisition and clearance

**Figure 4:**
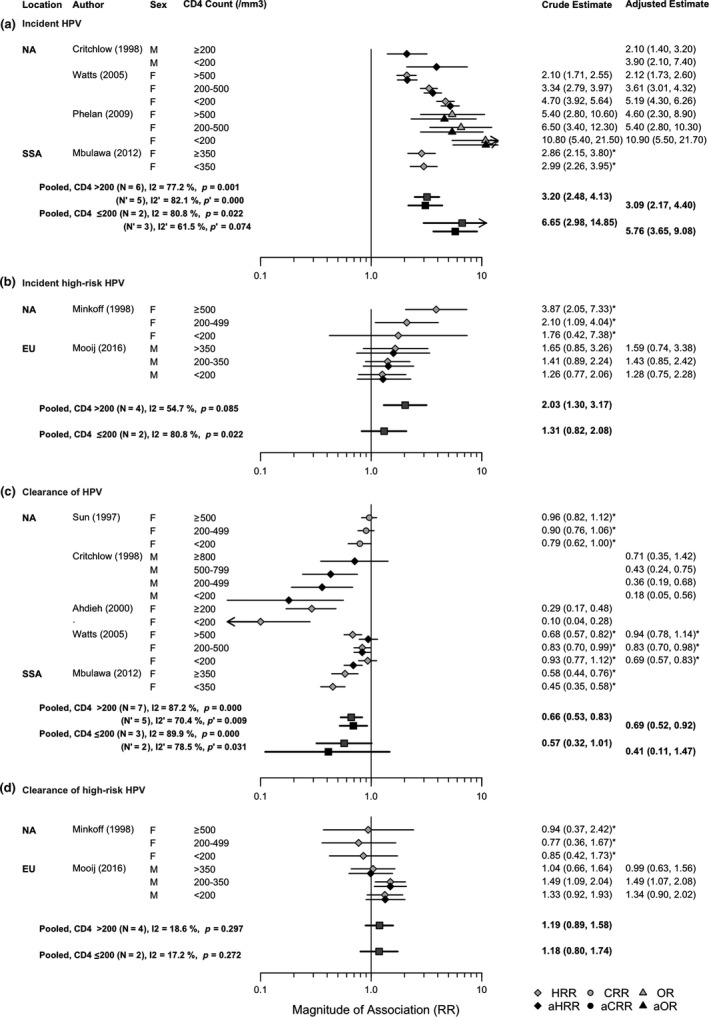
Forest plot for the crude and adjusted relative risk (RR) of HPV incidence and clearance among PLHIV by CD4 count level compared to HIV‐negative for: (a) incident HPV infection; (b) incident HR‐HPV infection; (c) clearance of HPV; (d) clearance of HR‐HPV. In this plot all HIV infection is prevalent, and the comparison group (unexposed group) is those HIV‐negative. An effect estimate greater than 1 (incident HPV) indicates increased HPV incidence in those with HIV infection compared to HIV‐negative individuals. An effect estimate less than 1 (HPV clearance) indicates decreased rate of HPV clearance in those with HIV infection compared to HIV‐negative individuals. An asterisk next to the effect estimate indicates that the estimate was derived from available information in the publication. For Watts (2005) the estimate refers to clearance of incident HPV types. NA, North America; EU, Europe; SSA, Sub‐Saharan Africa.

Figure 4 compares crude and adjusted study estimates of the association between HIV and subsequent HPV incidence and clearance for HPV and HR‐HPV, by CD4 count, compared to HIV‐negative individuals. No results were reported by CD4 count for LR‐HPV. The pooled crude RR for the association between HIV and incidence of HPV by CD4 count was higher for CD4 level ≤200 cells/μL (pooled RR = 6.65, 95% CI: 2.98 to 14.85; pooled aRR = 5.76, 95% CI: 3.65 to 9.08) than CD4 level >200 cells/μL (pooled RR = 3.20, 95% CI: 2.48 to 4.13; pooled aRR = 3.09, 95% CI: 2.17 to 4.40), but not statistically significantly so (95% CI overlapped). Two of the four available studies reported a test for trend and both showed a statistically significant increase in HIV risk with declining CD4 level (*p *= 0.03, *p* < 0.01; Table S1; see Appendix S1) 17, 24.

No other associations comparing CD4 level ≤200 cells/μL and CD4 level >200 cells/μL were found (Figure 4b,c,d). The two studies providing estimates by CD4 count for incident HR‐HPV by HIV status (Figure 4b) also reported a test for trend: one was not statistically significant 70, while the other one was statistically significant (*p* = 0.04) but suggested that incident HR‐HPV infection risk declined with decreasing CD4 count 35 (Table S1). Pooled estimates from the five studies that measured the effect of HIV on HPV clearance by CD4 count suggested that lower CD4 count reduced clearance of HPV, but non‐statistically significantly so (Figure 4c). Of the two studies reporting a test of trend one was statistically significant and the other was not (*p *= 0.2^17^ and *p* = 0.001^34^ (Table S1). Type‐specific estimates by CD4 count for HPV‐16/18/31/45 were reported by three studies and were not statistically significant (Figure S2a,b; see Appendix S1) 23, 25, 70.

### 3.4 Meta‐analysis results for review 2

#### 3.4.1 HIV acquisition by HPV status

**Figure 5:**
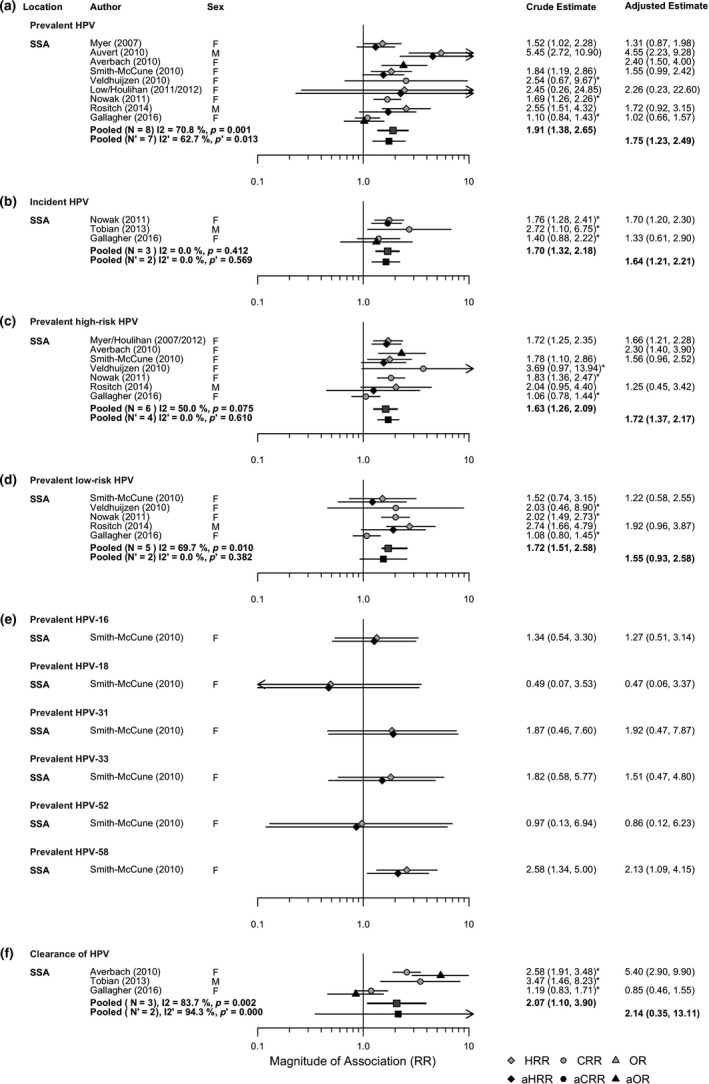
Forest plots of the crude and adjusted relative risk (RR) of HIV acquisition for: **(a)** prevalent HPV infection; **(b)** incident HPV infection; **(c)** prevalent HR‐HPV infection; **(d)** prevalent LR‐HPV infection; **(e)** HPV‐16/HPV‐18/HPV‐31/HPV‐33/HPV‐52/HPV‐58 infection; **(f)** clearance of HPV. In this plot HPV infection is the exposure and HIV acquisition is the outcome. An asterisk next to the effect estimate indicates that this estimate was calculated using data presented in the publication. SSA, Sub‐Saharan Africa.

Forest plots of pooled estimates for the association between HPV and subsequent HIV acquisition suggested an approximate doubling of HIV incidence among individuals infected with prevalent HPV (pooled RR = 1.91, 95% CI: 1.38 to 2.65; pooled aRR = 1.75, 95% CI: 1.23 to 2.49), prevalent HR‐HPV (pooled RR = 1.63, 95% CI: 1.26 to 2.09; pooled aRR = 1.72, 95% CI: 1.37 to 2.17), and prevalent LR‐HPV (pooled RR = 1.72, 95% CI: 1.51 to 2.58; pooled aRR = 1.55, 95% CI: 0.93 to 2.58) (Figure 5a,c,d). Although statistical heterogeneity across study estimates varied from 0% to 71%, study estimates were consistently above one. Only one study reported estimates for single prevalent HR‐HPV nonavalent vaccine types with wide confidence intervals, none of which was statistically significant apart from HPV‐58 (individual RR = 2.58, 95% CI: 1.34 to 5.00; individual aRR = 2.13, 95% CI: 1.09 to 4.15). The effect of incident HPV (pooled RR = 1.70, 95% CI: 1.32 to 2.18; pooled aRR = 1.64, 95% CI: 1.21 to 2.21) and clearance of HPV (pooled RR = 2.07, 95% CI: 1.10 to 3.90; pooled aRR = 2.14, 95% CI: 0.35 to 13.11) on subsequent HIV acquisition was similar to that for prevalent HPV (Figure 5b,f).

In subgroup analysis, the magnitude of the pooled crude RR and adjusted aRR were not greatly influenced by participant and study characteristics (Table S5; Appendix S1). Again, the only stratified pooled estimates that were not statistically significantly different than the null typically included only one or two studies.

The risk of HIV acquisition increased with the number of HPV types (aRR = 1.22 for increase in [any] HPV and aRR = 1.59 for HR‐HPV) (Table 3). Pooled estimates using slightly different comparison groups for HPV exposure status suggested similar magnitude of association to those using HPV‐negative as the comparison (Table 3).

**Table 3:** Further pooled estimates for dose–response and for additional exposures for the effect of HPV infection on HIV acquisition (review 2)

Exposure	Crude pooled			Adjusted pooled		
	RR (95% CI)	N	I^2^ (*p* value)	aRR (95% CI)	N	I^2^ (*p* value)
a. Dose–response						
1 prevalent HPV type (*vs*. HPV‐negative)	1.36 (0.77, 2.40)	3	55.2% (0.107)	1.60 (1.06, 2.42)	3	0.0% (0.715)
≥2 prevalent HPV types (*vs*. HPV‐negative)	2.30 (1.79, 2.95)	3	0.0% (0.497)	2.12 (1.21, 3.71)	2	11.3% (0.288)
Increase in HIV risk with no. of HPV types	1.30 (1.19, 1.42)	2	0.0% (0.331)	1.22 (1.07, 1.39)	2	0.0% (0.344)
Increase in HIV risk with no. of HR‐HPV types^b^		c		1.59 (1.22, 2.09)	2	0.0% (0.758)
b. Additional exposures						
Prevalent HR‐HPV (*vs*. HR‐HPV‐negative)^a^^,^ b	2.26 (1.39, 3.68)	5	68.4% (0.013)		c	
Prevalent LR‐HPV (*vs*. LR‐HPV‐negative)^a^	1.81 (1.44, 2.27)	4	0.0% (0.752)		c	
Prevalent HPV‐16 or HPV‐18 (*vs*. HPV‐16 and HPV‐18 negative)^a^	1.84 (1.25, 2.69)	2	0.0% (0.554)	1.20 (0.74, 1.94)	3	16.9% (0.300)
Prevalent HPV‐6, HPV‐11, HPV‐16 or HPV‐18 (*vs*. HPV‐6, HPV‐11, HPV‐16 and HPV‐18 negative)^a^	1.62 (1.11, 2.36)	2	0.0% (0.964)	1.34 (0.88, 2.04)	3	14.3% (0.311)
Prevalent HPV‐6, HPV‐11, HPV‐16, HPV‐18, HPV‐31, HPV‐33, HPV‐45, HPV‐52 or HPV‐58 (*vs*. HPV‐6, HPV‐11, HPV‐16, HPV‐18, HPV‐31, HPV‐33, HPV‐45, HPV‐52 and HPV‐58 negative)^a^		c		1.92 (1.06, 3.49)	2	52.4% (0.147)

a Other types are possible.

b Includes Auvert (2010) which had a high possibility of reverse causality.

c Indicates pooling not possible due to N < 2.

### 3.5 Study quality and publication bias

In review 1, 13 studies reported on the association between HIV and incident HPV infection for first HPV and 10 for new HPV (Table 1). Clearance was defined either as the loss of detection of any HPV type (N = 8) or of all HPV types (N = 17). Of the few studies that conducted adjusted analysis for key confounding variables, most adjusted for number of sexual partners (N = 12) and fewer studies adjusted for other key factors (HSV‐2: N = 3; hormonal contraception: N = 2; male circumcision: N = 2; condom use: N = 5). In review 2, a larger fraction of studies reported adjusted estimates (HSV‐2: N = 8; number of sexual partners: N = 6; hormonal contraception: N = 2; male circumcision N = 4; condom use: N = 8). In both reviews, most studies used individuals as the unit of analysis rather than using HPV type or visit, and in most studies the frequency of study visits was bi‐annually or more often over follow‐up period ranging between 6 and 53 months.

In our quantitative assessment of study quality using subgroup analyses, stratified pooled estimates (adjusted or unadjusted) by definitions of HPV incidence and clearance and unit of analysis were similar in magnitude to unstratified pooled estimates, and the associations generally remained statistically significant overall (Table 2 and Table S5; Appendix S1). There was some evidence in review 2 that adjustment for key confounders strengthened the associations (Table S5; Appendix S1). We did not find any particular trend when comparing crude and adjusted estimates for the subset of studies which reported both. Qualitatively, in both reviews we found evidence that exposed and unexposed groups were often different at baseline with respect to sexual risk factors or else this information was not reported, while for review 1, PLHIV had higher baseline HPV prevalence than HIV‐negative individuals (Tables 3 and S4; Appendix S1).

In review 1, there was little evidence of publication bias. In funnel plots (Figures S3,S4,S5,S6; see Appendix S1) most estimates fell within the expected 95% CI area. In subgroup analysis, pooled crude RR derived from directly reported study estimates were somewhat higher than those derived from available data for HPV, but not statistically significantly so (pooled RR = 2.50, 95% CI: 1.13 to 5.54 *vs*. 1.51, 95% CI: 1.25 to 1.83), and not for HR‐HPV (pooled RR = 2.09, 95% CI: 1.69 to 2.59 *vs*. 2.60, 95% CI: 1.93 to 3.49) (Table 2a,b). Our qualitative assessment found some evidence of selective reporting of estimates (Tables S1,S3; see Appendix S1), but this was not observed consistently for any specific association. There was some evidence of publication bias in review 2. Although crude estimates generally fell within the expected 95% CI bounds of the funnel plots, they tended to be asymmetrically distributed towards more significant values (demonstrated by Egger's regression line) (Figures S7,S8; see Appendix S1). In subgroup analysis, pooled crude RR for HIV incidence following exposure to prevalent HPV based on estimates directly reported in the study were higher than those derived from available data (pooled RR: 2.34, 95% CI: 1.51 to 3.62 *vs*. 1.42, 95% CI: 0.96 to 2.11), but not statistically significantly so (95% CI overlapped) (Table S5a; Appendix S1). The same was true for HR‐HPV (pooled RR: 1.77, 95% CI: 1.38 to 2.27 *vs*. 1.57, 95% CI: 0.93 to 2.65) (Table S5c; Appendix S1). Our qualitative assessment found evidence of selective reporting of estimates for LR‐HPV, dose–response results and non‐significant results (Tables S2,S4; see Appendix S1).

### 3.6 Summary of results

Our results indicate that the risk of subsequent HPV acquisition is approximately doubled in the presence of HIV infection, while the rate of HPV clearance is approximately halved. We updated previous meta‐analyses of the effect of HPV on subsequent HIV infection adding two new studies to earlier published reviews 12, 13. Consistent with previous pooled estimates, we observed a nearly doubling of HIV acquisition in those individuals with HPV infection 12, 13. In both of our reviews the magnitude of the associations was similar for LR‐HPV, HR‐HPV and timing of HPV infection (incident, prevalent or cleared infection), and was not influenced by any particular participant or study characteristics, including study quality indicators. There were some indications from the few studies available that HPV acquisition and persistence increased as CD4 level declined (review 1). There was also some evidence of a dose–response between number of HPV types and HIV acquisition risk (review 2), which may reflect increased biological HIV susceptibility with increasing number of HPV infections, though could also be a marker of shared risk behaviour 71 or some immunological susceptibility.

### 3.7 Strengths and limitations

To the best of our knowledge, this is the first meta‐analytic review investigating all the evidence for the association between HIV and subsequent HPV infection (review 1), not just in women 72 (published after our review was carried out). Our review adds further studies to those found in earlier reviews of HIV‐HPV infection interactions 12, 13, 72. Our review strengthens the evidence that HIV increases disease burden among PLHIV not only by accelerating HPV disease progression, but also by increasing HPV acquisition risk and persistence 72, 73, 74. Our two reviews collectively assessed the strength of the evidence for synergistic interactions between HIV and HPV infections. We explored in detail the influence of participant and study characteristics including study quality on pooled estimates, and explored possible publication and measurement biases. Results were consistent in subgroup analyses, by exposure and outcome definitions, between crude and adjusted estimates, and across alternative comparison groups.

As with other systematic and meta‐analytic reviews of longitudinal studies of STI and HIV interactions, there are some limitations to our reviews that may affect our results in either direction 12, 13, 72, 75. HPV and HIV are both STIs associated with similar sexual risk factors, which may lead to overestimation of the magnitude of STI and HIV associations. In our review, study‐level adjustment for key confounders, such as number of sexual partners, condom use and HSV‐2 was more frequent for studies included in review 2 than review 1. Recent modelling analyses of HPV and HIV, and of HSV‐2 and HIV, have suggested that observed associations could be explained by confounding by sexual risk factors, in the absence of biological interaction 76, 77. However, these effects are likely to be greater for cross‐sectional studies whereas our review was based on longitudinal studies 78, 79. Furthermore, we found some evidence in review 2 that adjustment for key confounding variables including sexual behaviour actually strengthened the associations. That said, even with adjusted estimates the presence of residual confounding cannot totally be excluded, especially by partner and/or partnership characteristics (influencing the likelihood of exposure to HPV and HIV) or previous HPV infections (which may protect or predispose to HPV infection) 71.

Conversely, mathematical modelling to assess potential biases in STI and HIV interaction estimates for different study designs has suggested that whilst statistical adjustment can help reduce overestimation due to confounding, the magnitude of association could also be underestimated in the presence of misclassification of exposure 78, 79. Although we only included longitudinal studies, which established the sequence of events for HPV and HIV infections, there is still some potential for misclassification bias of both HIV and HPV exposure status. This is more likely to be an issue for HPV as the exposure (review 2), since HPV infection is a common infection with a finite duration (in contrast to HIV). However, most studies in review 2 assessed HPV status both at baseline and during follow‐up. Furthermore, over half of the studies in both reviews carried out STI and HIV testing at least every 6 months. In any case, misclassification bias is generally expected to bias RR estimates toward the null value, underestimating the magnitude of association, which would not invalidate our conclusions. Indeed, an early modelling study also showed that, even in the presence of biological interactions, estimates of the role of STI on HIV acquisition can also be substantially under‐estimated, mainly due to imprecise measurement of STI exposure resulting from less frequent STI testing (especially >6 months) and longer window periods of HIV testing (especially >6 weeks) 79 (Guibord P, MSc Thesis).

Some studies reported multiple estimates of association for different HPV exposures (review 2) and outcomes (review 1) (i.e. HR‐HPV, LR‐HPV, [any] HPV), which also provide a form of validation of consistency of results within a study, but may however increase the likelihood of finding spurious associations. We also differentiated between “first HPV”, defined as the acquisition of HPV DNA in those individuals without any HPV‐DNA present at baseline but who may have been exposed to HPV in the past (i.e. not naïve), and “new HPV”, defined as the acquisition of DNA of a new and different HPV type in those individuals who already had HPV DNA present at baseline. We also differentiated between clearance of “all” and “any” HPV. Definition of a clearance event itself (i.e. on the basis of only one, or successive negative tests) also varied between studies. However, we did not find evidence that these differences influenced our results.

The studies in review 2 were concentrated in Sub‐Saharan Africa, which may limit the generalizability of our results to other settings. There were fewer study estimates for single HPV vaccine types and LR‐HPV associations (both reviews), HPV incidence and clearance by CD4 count level (review 1) and HIV acquisition by incidence and clearance of HPV (review 2). This could partly be due to publication bias, of which we found greater evidence for review 2 than review 1. This was observed in funnel plots, in subgroup analysis comparing reported estimates that were slightly higher that estimates that we calculated ourselves, and in our qualitative assessment of selective reporting of statistically significant associations. Nevertheless, we were able to successfully derive several new RR study estimates from available data (where the estimates themselves were not reported), which improved the precision of crude pooled estimates and reduced publication biases. It also meant that we were not able to derive adjusted estimates controlling for potential confounders, and as a proportion of studies fewer adjusted estimates were available for review 1 than 2. Heterogeneity (based on the *I* ^2^ statistic), was particularly high across estimates of HIV on HPV clearance, but results from subgroup analyses showed that pooled effect sizes remained similar across the subgroups examined.

In our review we have comprehensively summarized all the available observational evidence on HIV and HPV interactions. Our review was designed to minimize and assess the possibility of confounding and bias affecting our findings. We restricted our analysis to longitudinal studies of the associations, and carried out extensive sensitivity analyses, comparing adjusted and unadjusted estimates in multiple ways, extracting information on the comparability of exposed and unexposed groups, and exploring the influence of possible selective reporting of non‐significant results. The likelihood of confounding explaining the associations in not the only consideration when assessing the plausibility for causation. For example, our results meet many of the 9 Bradford Hill criteria for causality 80, which strengthens the case for the existence of biological interactions between HIV and HPV. The *strength* of the associations was *consistent* across HPV types and study and population characteristics. Longitudinal studies maximize the likelihood of the exposure preceding the outcome, that is, minimize the risk of reverse causation (*timing*). We found some evidence of a *dose–response* (HPV acquisition by CD4 cell count compared to HIV‐negative individuals, and effect of number of HPV types on HIV acquisition). Additional evidence of a biological dose–response also comes from studies comparing HPV acquisition and clearance by CD4 levels among PLHIV only, which was not the purpose of our study 72, 81, 82. The associations found are also *biologically plausible* 83. HIV may increase susceptibility to HPV infection and persistence of HPV infection among PLHIV due to immunodeficiency, the inflammatory response to HIV infection, immune dysregulation at the site of HIV infection, and/or the effects of HIV on HPV transcription and translation 73, 84, 85. A recent meta‐analysis suggested that women living with HIV (WLHIV) on ART had lower HR‐HPV prevalence than those not on ART after adjusting for CD4 cell count and ART duration, suggesting that ART may repair some of the damages induced by HIV immunodeficiency 11. HPV may in turn directly facilitate HIV acquisition by increasing or altering the density of HIV target cells (such as T lymphocytes and Langerhans cells) and weakening the physical epithelial barrier to HIV 86. Whilst we found that the magnitude of the association was similar by timing of HPV infection (incident, prevalent or cleared infection), differing types and density of immunological cells in the genital area could in theory translate into varying risk of HIV acquisition over the course of HPV infection and warrants further investigation. A recent systematic review and meta‐analysis of 57 studies found higher risk of HIV acquisition in those with incident HSV‐2 infection (compared to those without HSV‐2 infection) than was found for prevalent HSV‐2 infection 75. The interactions between HIV and HPV could be *analogous* to the interactions between HIV and HSV‐2 in terms of some similarity of biological mechanisms. In future, better evidence of these interactions could be obtained from discordant couple studies and/or studies with more frequent STI and HIV testing, using the best HIV tests with the shortest window period, and by adequately measuring potential sexual risk factors among participants and their partners 71.

## 4 Conclusions

Our study provides an evidence base for multiple biological interactions between HIV and HPV. These interactions have a number of important clinical, epidemiological and public health implications. In particular, the excess burden of HPV in PLHIV has implications for the clinical management of PLHIV requiring more frequent screening, follow‐up and management of precancerous lesions due to HPV. HPV vaccination, which has been proven to be safe and immunogenic among PLHIV, may confer particular benefit to this group 87, and help to control HPV infections and related cancer more efficiently at population‐level 7. Recent modelling studies suggest that as PLHIV are disproportionately infected with HPV they are more likely to transmit it making them an important group for focused HPV prevention 88. Increasing HPV vaccination coverage in low‐ and middle‐income countries, in particular among PLHIV, is an aim of the Pink Ribbon Red Ribbon (PRRR) initiative, launched by the George W. Bush Institute, the United States Government through the U.S. President's Emergency Plan for AIDS Relief (PEPFAR), and the Joint United Nations Programme on HIV/AIDS (UNAIDS) 89, 90, 91.

In addition, interventions such as HPV vaccination could in theory have additional indirect benefits on HIV/AIDS, even if the relative risk of HIV acquisition due to HPV is modest. Given the burden of HPV and HIV and abundance of co‐infections, HPV vaccination could prevent a non‐negligible number of AIDS deaths particularly in Sub‐Saharan Africa. If reducing HPV provides benefits for HIV, this would optimize HPV programmes, particularly in the context of combination prevention, providing additional incentives for programme implementation. Our comprehensive review can help inform decisions on HPV vaccination in women and men, including MSM. Mathematical modelling studies are needed to understand the potential impact of these multiple interactions on HIV and HPV trends and in the context of intervention rollout for high HIV prevalence settings.

## Competing interests

MB reports an unrestricted grant from Merck in the past 3 years in relation to herpes zoster (completed). MD declares a role as a consultant to GlaxoSmithKline (GSK) in the past 3 years in relation to herpes zoster vaccine. PM has received funding from GlaxoSmithKline (GSK) and in kind donation from QIAGEN for research projects on HPV vaccines or HPV testing. KJL, MMR, PMB and MCB declare no conflicts of interest.

## Authors’ contributions

MCB designed and supervised the study and provided technical guidance. The literature search was done by KJL. KJL and MMR extracted the data and performed the analysis. KJL produced the first draft of the manuscript, which was subsequently revised and edited by MCB and MMR. All authors contributed to the interpretation of results, contributed technical expertise, commented on the drafts, and approved the final version.

## Supporting information


**Appendix S1.** Additional supporting information.
**Figure S1.** Forest plots of the crude relative risk (RR) by HIV status of: (S1a) incident LR‐HPV infection; (S1b) incident HPV‐6/HPV‐11 infection; (S1c) clearance of LR‐HPV.
**Figure S2.** Forest plots of the crude and adjusted relative risk (RR) by HIV status of: (S2a) incident HPV‐16/HPV‐18 infection by CD4 cell count; (S2b) clearance of HPV‐16/HPV‐18/HPV‐31/HPV‐45 by CD4 cell count.
**Figure S3.** Funnel plot of the crude estimates for HIV on incident HPV.
**Figure S4.** Funnel plot of the crude estimates for HIV on incident HR‐HPV.
**Figure S5.** Funnel plot of the crude estimates for HIV on clearance of HPV.
**Figure S6.** Funnel plot of the crude estimates for HIV on clearance of HR‐HPV.
**Figure S7.** Funnel plot of the crude estimates for prevalent HPV on HIV.
**Figure S8.** Funnel plot of the crude estimates for prevalent HR‐HPV on HIV.
**Table S1.** Summary of the 41 publications identified reporting on the effect of HIV infection on HPV acquisition and clearance (review 1).
**Table S2.** Summary of the 15 publications identified reporting on the effect of HPV infection or clearance on HIV acquisition (review 2).
**Table S3.** Additional study characteristics used to assess quality of studies from publications included in the review of longitudinal studies of the effect of HIV on HPV acquisition and clearance (review 1).
**Table S4.** Additional study characteristics used to assess quality of studies from publications included in the review of longitudinal studies of the effect of HPV infection and clearance on HIV (review 2).
**Table S5.** Subgroup analyses of the association between prior exposure to HPV and subsequent HIV infection by participant and study characteristics (review 2).
