# Supplementary material for: Evidence of synergistic relationships between HIV and Human Papillomavirus (HPV): systematic reviews and meta‐analyses of longitudinal studies of HPV acquisition and clearance by HIV status, and of HIV acquisition by HPV status
Source: J Int AIDS Soc. 2018 Jun 5;21(6):e25110. doi: 10.1002/jia2.25110 (PMC5989783; doi:10.1002/jia2.25110)
Supplement: Supplementary file 1 — Appendix S1. Additional supporting information. Figure S1. Forest plots of the crude relative risk (RR) by HIV status of: (S1a) incident LR‐HPV infection; (S1b) incident HPV‐6/HPV‐11 infection; (S1c) clearance of LR‐HPV. Figure S2. Forest plots of the crude and adjusted relative risk (RR) by HIV status of: (S2a) incident HPV‐16/HPV‐18 infection by CD4 cell count; (S2b) clearance of HPV‐16/HPV‐18/HPV‐31/HPV‐45 by CD4 cell count. Figure S3. Funnel plot of the crude estimates for HIV on incident HPV. Figure S4. Funnel plot of the crude estimates for HIV on incident HR‐HPV. Figure S5. Funnel plot of the crude estimates for HIV on clearance of HPV. Figure S6. Funnel plot of the crude estimates for HIV on clearance of HR‐HPV. Figure S7. Funnel plot of the crude estimates for prevalent HPV on HIV. Figure S8. Funnel plot of the crude estimates for prevalent HR‐HPV on HIV. Table S1. Summary of the 41 publications identified reporting on the effect of HIV infection on HPV acquisition and clearance (review 1). Table S2. Summary of the 15 publications identified reporting on the effect of HPV infection or clearance on HIV acquisition (review 2). Table S3. Additional study characteristics used to assess quality of studies from publications included in the review of longitudinal studies of the effect of HIV on HPV acquisition and clearance (review 1). Table S4. Additional study characteristics used to assess quality of studies from publications included in the review of longitudinal studies of the effect of HPV infection and clearance on HIV (review 2). Table S5. Subgroup analyses of the association between prior exposure to HPV and subsequent HIV infection by participant and study characteristics (review 2). [file JIA2-21-e25110-s001.pdf]

Evidence of synergistic relationships between HIV and Human Papillomavirus (HPV): systematic reviews and meta-analyses of longitudinal studies of HPV acquisition and clearance by HIV status, and of HIV acquisition by HPV status

Katharine J Looker, Minttu M Rönn, Patrick Brock, Marc Brisson, Melanie Drolet, Philippe Mayaud, Marie-Claude Boily

FURTHER DETAILS ON THE METHODS

A: Literature search terms

**PubMed database**
**Search 1:** (hpv[Title] OR human papillomavirus[Title] OR human papilloma virus[Title]) AND (hiv[Title] OR human immunodeficiency virus[Title])
**Search 2:** (genit*[Title] OR anal[Title] OR anus[Title] OR cervix[Title] OR cervical[Title]) AND (neoplas*[Title] OR lesion*[Title]) OR sil[Title] OR cancer*[Title] OR carcinoma*[Title] OR dysplas*[Title] OR disease*[Title] OR squamous[Title] OR abnormalit*[Title]) AND (hiv[Title] OR human immunodeficiency virus[Title]) AND (incidence[MeSH Terms] OR disease progression[MeSH Terms] OR longitudinal study[MeSH Terms] OR time factor[MeSH Terms] OR prospective study[MeSH Terms] OR cohort study[MeSH Terms]) OR survival analysis[MeSH Terms] OR follow-up study[MeSH Terms])
**Search 3:** (papillomaviridae[MeSH Terms] OR papillomavirus infections[MeSH Terms]) AND (human immunodeficiency virus[MeSH Terms] OR hiv infection[MeSH Terms]) AND (incidence[MeSH Terms] OR disease progression[MeSH Terms] OR longitudinal study[MeSH Terms] OR time factor[MeSH Terms] OR prospective study[MeSH Terms] OR cohort study[MeSH Terms]) OR survival analysis[MeSH Terms] OR follow-up study[MeSH Terms])
**Embase database**
**Search:** (hiv or human immunodeficiency virus).af. AND (hpv or human papillomavirus or human papilloma virus).af. (af=any field)

We excluded unpublished studies and conference abstracts, and did not attempt to contact study authors.

B. Derivation of crude estimates and 95%CIs using available data

Where crude estimates (or their 95%CIs) were not reported but sufficient information was provided, we calculated (in order of preference) the crude HRR (incidence rate ratio, IRR) or CRR and 95%CIs, using the formulae:

$\text{logIRR} = \ln((d_1/T_1)/(d_0/T_0))$

$\text{selogIRR} = \text{sqrt}(1/d_1 + 1/d_0)$

$\text{logCRR} = \ln((d_1/N_1)/(d_0/N_0))$

selogCRR = sqrt $(1/d_1 + 1/d_0 - 1/N_1 - 1/N_1)$

where d_0 are d_1 are the number of unexposed and exposed cases, respectively, N_0 and N_1 are the number in the unexposed and exposed population, respectively, and T_0 and T_1 are the total person-years at risk for the unexposed and exposed population, respectively.

The log (ln) estimate and the standard error of the log estimate were used for pooling. The standard error of the log estimate was calculated from data for numbers of individuals where possible. Where the standard error of the log estimate could not be calculated from data, and for crude hazard ratios and adjusted estimates, the standard error of the log estimate was calculated using the formula (ln UCB-ln LCB)/(2*1.96) where UCB and LCB are the upper and lower confidence bounds, respectively.

C. Funnel plots to assess publication bias within pooled estimates

The funnel plots were constructed using the metafunnel command in Stata(1, 2). The plot contrasts the log of the effect size (RR) and its standard error (SE). The center line is the fixed-effects summary estimate with pseudo 95% confidence intervals (summary effect $\pm$ 1.96 * SE using the SE from the studies). This gives the estimated area where 95% of studies are expected to fall in the absence of (statistical) heterogeneity. Also plotted is Egger's test of asymmetry, which draws a linear regression line through the estimates aiding in the assessment of the results.

FURTHER RESULTS

D. Additional forest plots and meta-analyses

Figure S1. Forest plots of the crude relative risk (RR) by HIV status of: S1a) incident LR-HPV infection; S1b) incident HPV-6/HPV-11 infection; S1c) clearance of LR-HPV

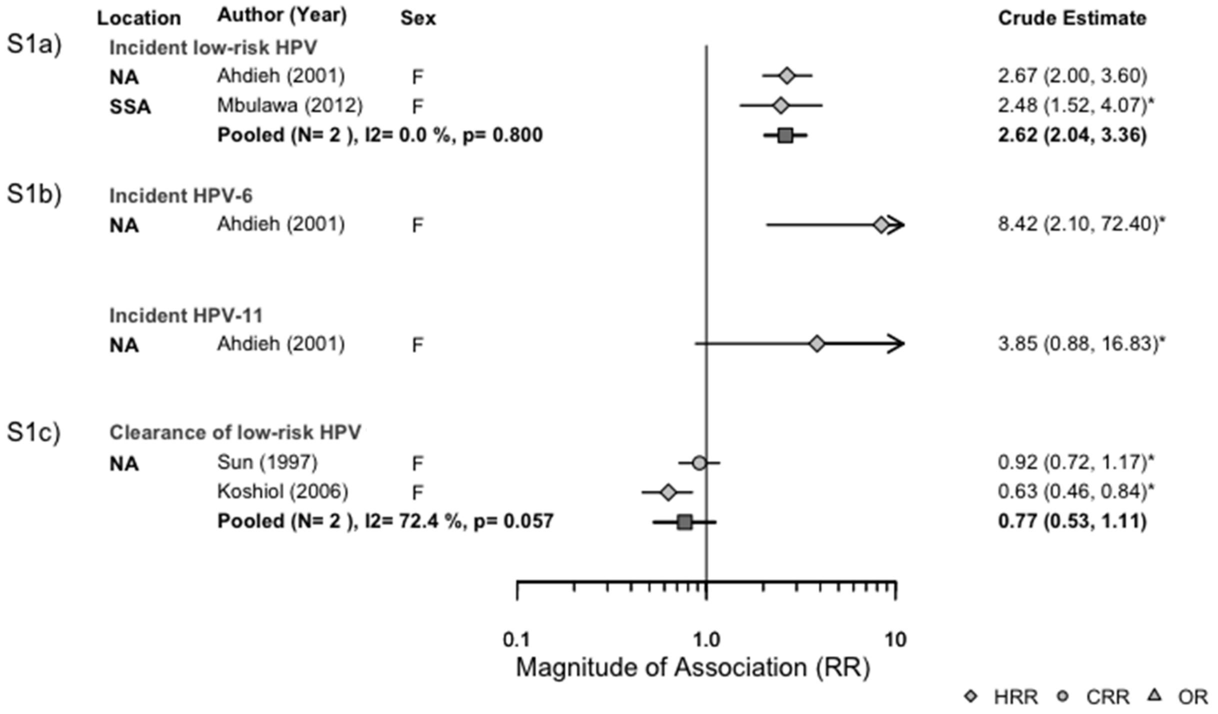

Footnote: In this plot all HIV infection is prevalent, and the comparison group (unexposed group) is those HIV-negative. An effect estimate greater than 1 (incident HPV) indicates increased HPV incidence in those with HIV infection compared to HIV-negative individuals. An effect estimate less than 1 (HPV clearance) indicates decreased rate of HPV clearance in those with HIV infection compared to HIV-negative individuals. An asterisk next to the effect estimate indicates that this estimate was calculated using data presented in the publication. NA – North America; SSA – Sub-Saharan Africa.

Figure S2. Forest plots of the crude and adjusted relative risk (RR) by HIV status of: S2a) incident HPV-16/HPV-18 infection by CD4 cell count; S2b) clearance of HPV-16/HPV-18/HPV-31/HPV-45 by CD4 cell count

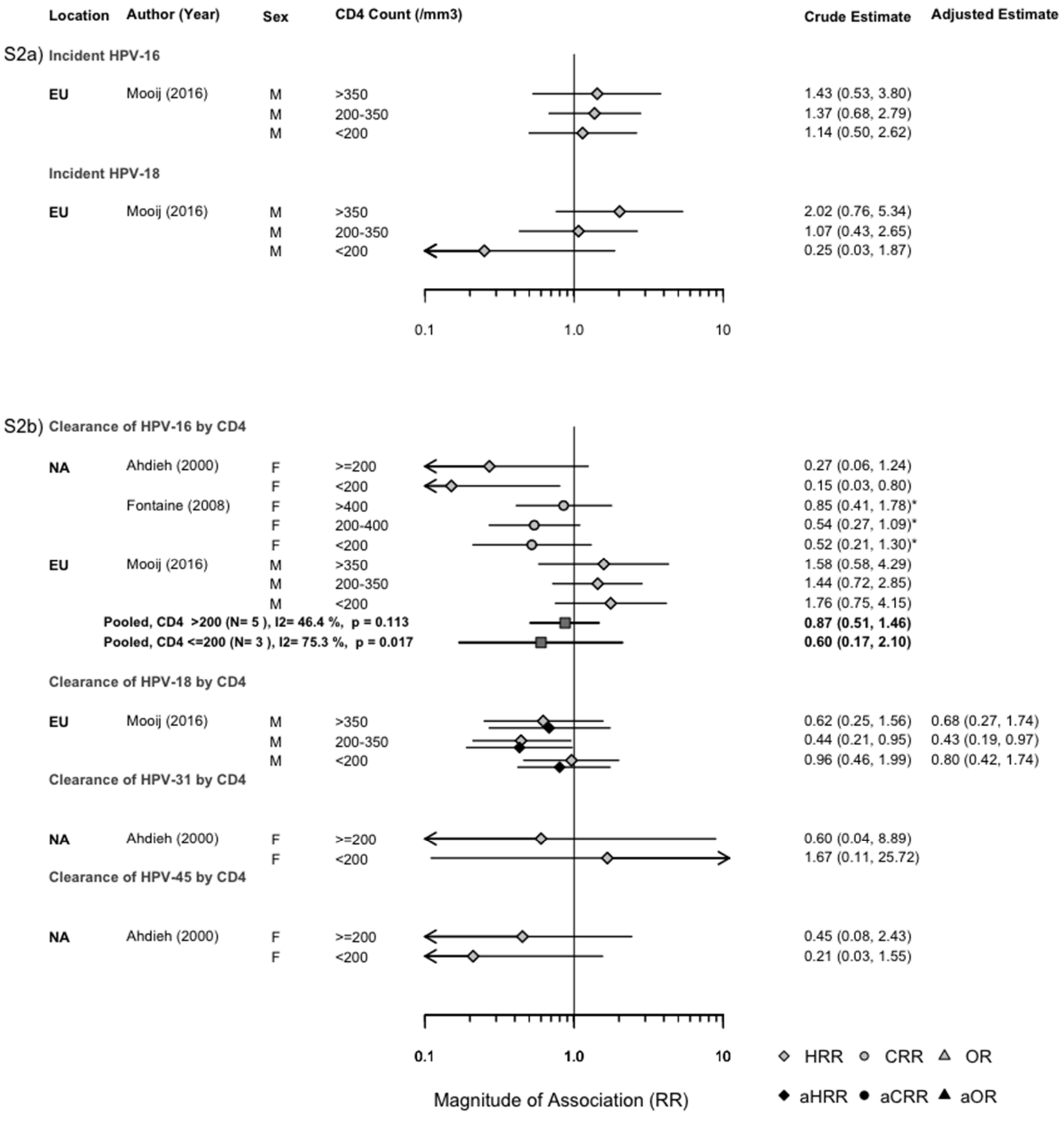

Footnote: In this plot all HIV infection is prevalent, and the comparison group (unexposed group) is those HIV-negative. An effect estimate greater than 1 (incident HPV) indicates increased HPV incidence in those with HIV infection compared to HIV-negative individuals. An effect estimate less than 1 (HPV clearance) indicates decreased rate of HPV clearance in those with HIV infection compared to HIV-negative individuals. An asterisk next to the effect estimate indicates that this estimate was calculated using data presented in the publication. NA – North America; EU- Europe.

E. Funnel plots to assess publication bias within crude estimates

Review 1: HPV acquisition and clearance by HIV status

Figure S3. Funnel plot of the crude estimates for incident HPV

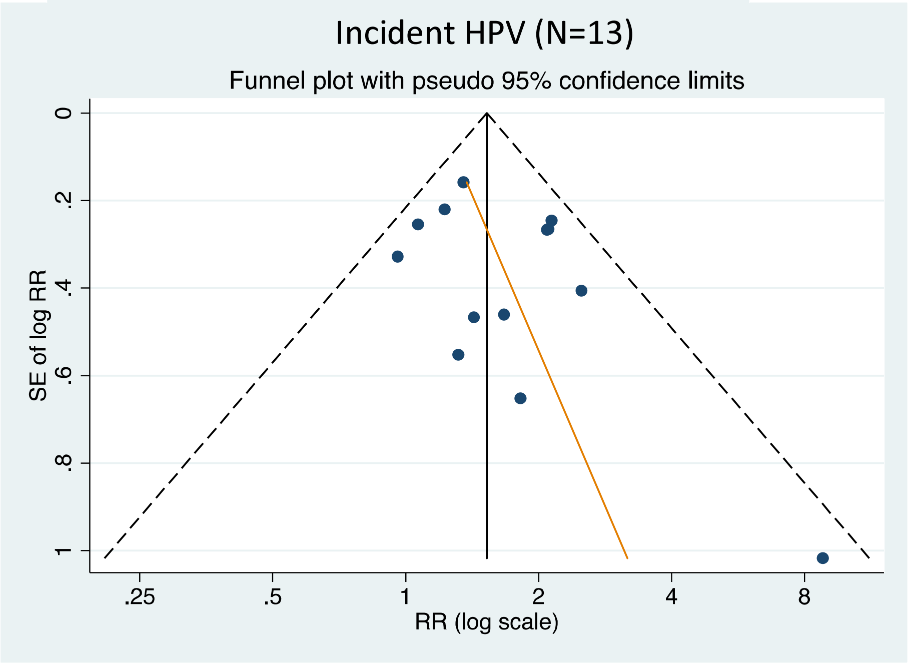

Note: two estimates are overlaying each other.

Figure S4. Funnel plot of the crude estimates for incident HR-HPV

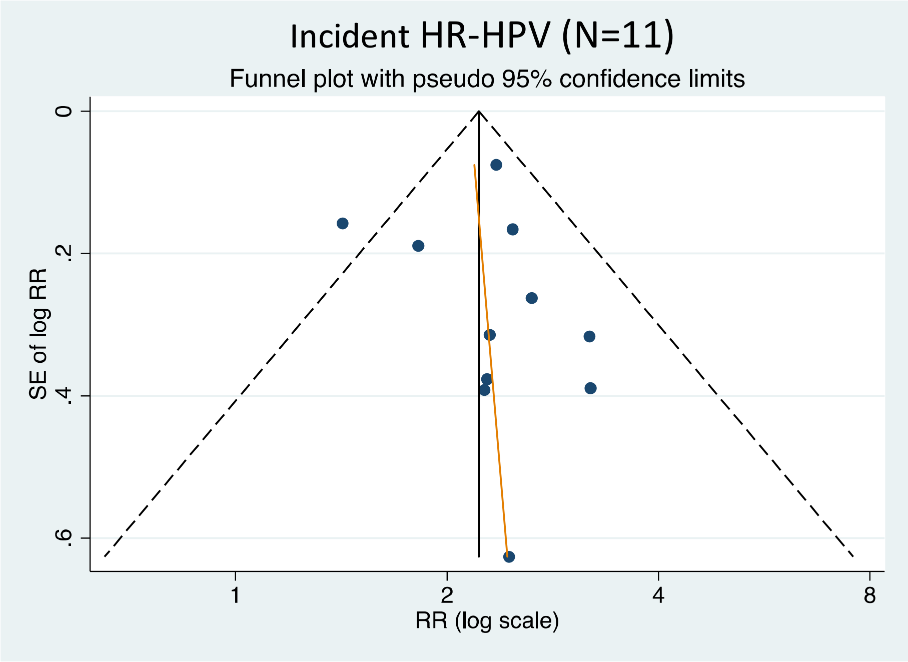

Figure S5. Funnel plot of the crude estimates for clearance of HPV

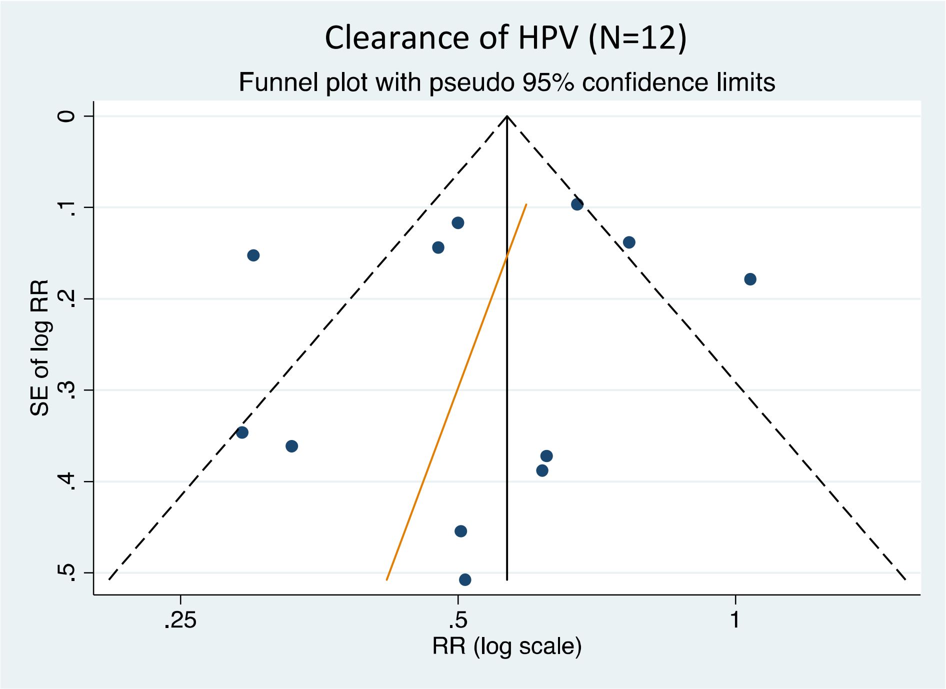

Figure S6. Funnel plot of the crude estimates for clearance of HR-HPV

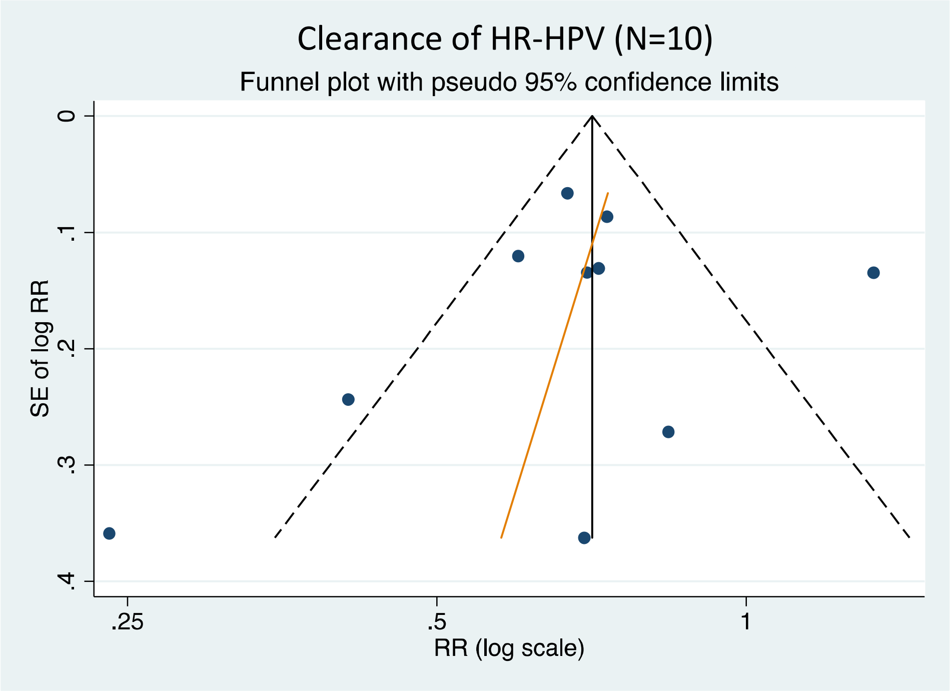

Review 2: HIV acquisition by HPV status

Figure S7. Funnel plot of the crude estimates for prevalent HPV

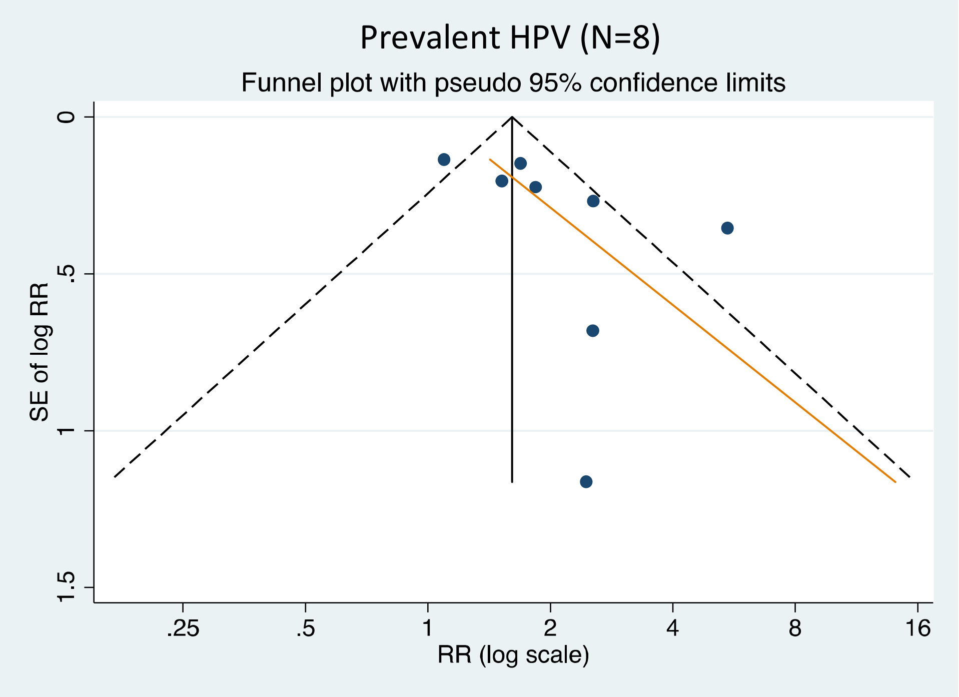

Figure S8. Funnel plot of the crude estimates for prevalent HR-HPV

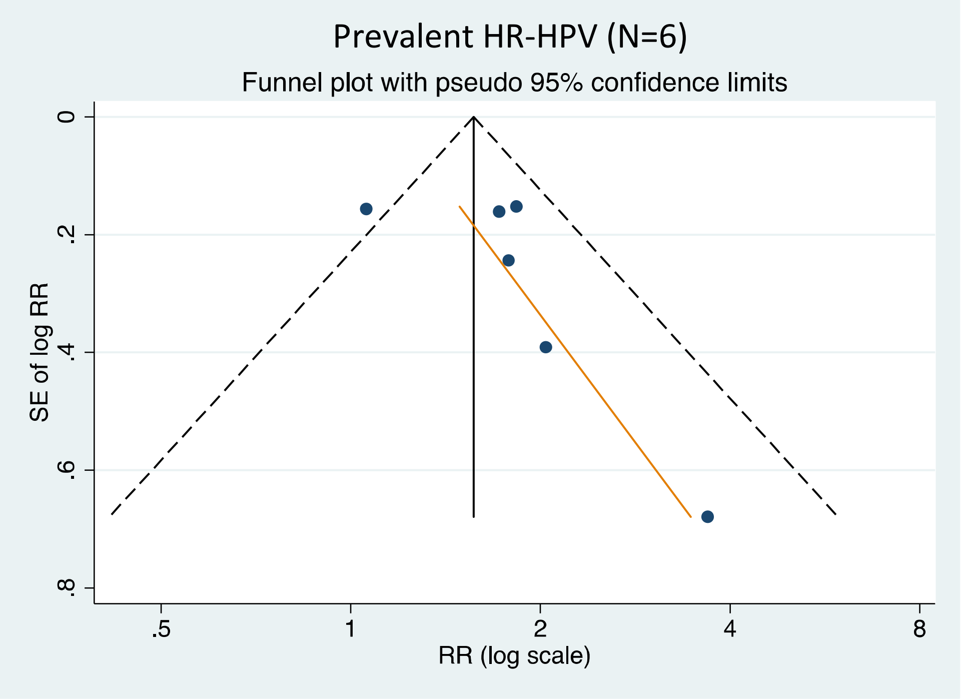

F. Supplementary tables

Table S1. Summary of the 41 publications identified reporting on the effect of HIV infection on HPV acquisition and clearance (review 1)

Source	Age (years)	Study period	Sample size[1]	Mean follow-up	Testing frequency	Outcome (vs Comparison group)	Study estimate (95%CI)				Fig.
							Crude		Adjusted		
**NORTH AMERICA**											
**USA - Reaching for Excellence in Adolescent Care & Health Project (REACH) project: Prospective biomedical, behavioral, & psychosocial cohort of at-risk HIV positive adolescents in multiple clinical sites in 13 cities**											
**Mullins (2013)(3)**	Mean = 17	1996-2000	261	22.4 mnths	HIV: B; Anal & genital HPV: B & 12 mnthly	First incident HPV (vs Remaining HPV-negative)	1.67 (0.68,4.11)[2]	HRR M	--		2a,NA
							2.14 (1.32,3.47)[2]	HRR F	--		2a,NA
						First incident HR-HPV (vs Remaining HR-HPV-negative)	2.45 (0.72,8.38)[2]	HRR M	--		2b,NA
							2.26 (1.05,4.88)[2]	HRR F	--		2b,NA
**Moscicki (2004)(4)**	Mean = 17	1996-2000	222	U	HIV: B; CD4: 3 mnthly; Genital HPV: B & 6 mnthly	Clearance of all HPV types (vs Persistent detection of HPV type)<br>(i.e. clearance = 2 consecutive negative tests in those positive at B)	--		0.72 (0.52,1.01)[2]	HRR F	NA,3a
**USA - Seattle AIDS Prevention Project: Prospective study of HIV & anal HPV infection among MSM in Seattle, Washington**											
**Critchlow (1998)(5)**	Mean = 34-36	1989-1997	505	29 mnths	HIV: B; CD4: B; Anal HPV: B & each visit (at least 2)	First incident HPV (vs Remaining HPV-negative)	1.35 (0.99,1.84)[2,5]	CRR M	1.8 (1.1,3.1)	HRR[17]M	2a,2a
						By CD4 level(cells/µL):	NS & NR	--	NS & NR	--	--
						New incident HPV (vs No new incident HPV)	1.35(0.99,1.84)[2,5]	CRR M	2.3 (1.5,3.4)	HRR[17]M	--
						By CD4 level (cells/µL):					
						<200	--		3.9 (2.1,7.4)[6]	HRR[17]M	NA,4a
						≥200	--		2.1 (1.4,3.2)[6]	HRR[17]M	NA,4a
						Test for trend comparing <200 & ≥ 200: p=0.03					
						Clearance of all HPV types (vs Persistent detection of HPV type)	--		0.4 (0.30,0.60)	HRR[17]M	NA,3a
						By CD4 level (cells/µL):					
						<200	--		0.18 (0.05,0.56)[7]	HRR[17]M	NA,4c
						200-499	--		0.36 (0.19,0.68)[7]	HRR[17]M	NA,4c
						500-799	--		0.43 (0.24,0.75)[7]	HRR[17]M	NA,4c
						≥800	--		0.71 (0.35,1.42)[7]	HRR[17]M	NA,4c
						(i.e. clearance = 1 negative HPV test during FUP among those positive at B)					
						Test for trend comparing <200, 200-499, 500-799 & ≥800: p=0.2					
**USA - US multicentre MSM cohort on HIV: Prospective cohort among MSM/bisexual part of a multicentre evaluation of HIV natural history**											
**Breese (1995)(6)**	Mean = 39-41	1989-1990	166	6 mnths	HIV: B only; Penile & anal HPV: B & 6 mnthly	First incident HPV (vs Remaining HPV-negative)	1.43 (0.57,3.56)[2]	CRR M	--		2a,NA
						Clearance of all HPV types (vs Persistent detection of HPV type)<br>(i.e. clearance = positive HPV test at 1st visit & negative test at 2nd visit vs positive at both)	0.62 (0.29,1.32)[2]	CRR M	--		3a,NA
**USA - HIV Epidemiology Research on Outcomes (HERO) study: Prospective cohort of former & current drug-using women with HIV or at HIV risk in the Bronx, New York**											
**Dev (2006)(7)**	Mean = 40	U	178	33.4 mnths	HIV: B; Genital HPV: B & 6 mnthly	First incident HPV (vs Remaining HPV-negative)	2.5 (1.1,5.4)	HRR F	3.4 (1.4,8.0)	HRR	2a,2b
						First incident HR-HPV (vs Remaining HR-HPV-negative)	3.2 (1.5,6.9)	HRR F	3.0 (1.4,6.6)	HRR	2b,2b

**Canada - Canadian Women's HIV Study (CWHS): Multicentre prospective cohort of HIV positive or at risk women in 28 institutions across Canada**											
**Blitz (2013)(8)**	Mean = 31	1993-2002	1073	24/14 mnths (5-51)	HIV, CD4 & genital HPV: B & 6 mnthly	First incident HR-HPV (vs Remaining HR-HPV-negative)	2.28 (1.09,4.77)	HRR F	--		2b,NA
						Incident HPV-16 (vs Remaining HPV-16 negative)	2.03 (0.75,5.52)	HRR F	--		2c,NA
						Incident HPV-18 (vs Remaining HPV-18 negative)	No unexposed cases[2]	--	--		--
						First incident HR-HPV excluding HPV-16/18 (vs Remaining negative for those types)	1.56 (0.73,3.32)	HRR F	--		--
						Clearance of all HR-HPV types (vs Persistent detection of HR-HPV type)	0.41 (0.25,0.65)	HRR F	--		3b,NA
						Clearance of HPV-16 (vs Persistent detection of HPV-16)	1.36 (0.52,3.62)	HRR F	--		3c,NA
						Clearance of HPV-18 (vs Persistent detection of HPV-18)	0.40 (0.22,0.72)[2]	CRR F	--		3c,NA
						Clearance of all HR-HPV excluding HPV-16/18 (vs Persistent detection of HR-HPV excluding HPV-16/18)	0.32 (0.19,0.55)	HRR F	--		--
						(i.e. clearance = negative test for relevant HPV types following a positive test at B)					
**Aho (2004)(9)**	Mean = 32	1993-2000	70	23.5 mnths	HIV, CD4 & genital HPV: B & 6 mnthly	Clearance of HPV-52 (vs Persistent detection of HPV-52)	1.04 (0.46,2.36)[2]	CRR F	--		3d,NA
						Clearance of HPV-52 excluding those HPV-52 positive at B (vs Persistent detection of HPV-52)	1.67 (0.25,11.04)[2]	CRR F	--		--
						By CD4 level (cells/µL):					
						<200	1.08 (0.12,9.89)[2,10]	CRR F	0.64 (0.03,11.11)[2]	OR F	--
						≥200	1.81 (0.27,12.20)[2,10]	CRR F	1.45 (0.10,20.00)[2]	OR F	--
						(i.e. clearance = 1 positive HPV-52 test followed by 1 negative test within 9 mnths; persistence = positive for HPV-52 for at least 9 mnths)					
**Gagnon (2004)(10)**	Mean = 31-32	1993-2000	62	26-36 mnths	HIV, CD4 & genital HPV: B & 6 mnthly	Clearance of HPV-33 (vs Persistent detection of HPV-33)	NS & NR	--	--		--
						By CD4 level (cells/µL)	NS & NR	--	--		--
						Clearance of HPV-35 (vs Persistent detection of HPV-33)	NS & NR	--	--		--
						By CD4 level (cells/µL)	NS & NR	--	--		--
						(i.e. persistence = 3 consecutive genital specimens containing the same variant for ≥ 12 mnths					
**Fontaine (2008)(11)**	Mean = 29-31	1993-2002	67	18-27 mnths	HIV, CD4 & genital HPV: B & 6 mnthly	Clearance of HPV-16 (vs Persistent detection of HPV-16)	0.58 (0.33,1.01)[2]	CRR F	--		3c,NA
						By CD4 level (cells/µL):					
						<200	0.52 (0.21,1.30)[2,8]	CRR F	--		S2b,NA
						200-400	0.54 (0.27,1.09)[2,8]	CRR F	--		S2b,NA
						>400	0.85 (0.41,1.78)[2,8]	CRR F	--		S2b,NA
						(i.e. clearance = infections for < 12 mnths; persistence = 3 consecutive genital specimens HPV-16 positive for ≥12 mnths)					
						Test for trend; comparison not clear: p=0.06					
**USA – The AIDS Link to Intravenous Experiences (ALIVE) study on HIV natural history: Prospective cohort gynecological substudy among high risk female injecting drug users in Baltimore, Maryland**											
**Phelan (2009)(12)**	Mean = 37	1992-1997	219	2-10 visits	HIV, CD4 & genital HPV: B & 6 mnthly	New incident HPV (vs No new incident HPV) (unit=visit)	--		--		--
						By CD4 level (cells/µL):					
						>500	5.4 (2.8,10.6)[9]	OR F	4.6 (2.3,8.9) SS	OR F	4a,4a
						200-500	6.5 (3.4,12.3)[9]	OR F	5.4 (2.8,10.3) SS	OR F	4a,4a
						<200	10.8 (5.4,21.5)[9]	OR F	10.9 (5.5,21.7) SS	OR F	4a,4a
						Test for trend comparing >500, 200-500 & <200: p<0.01					

**Ahdieh (2000)(13)**	Median = 36	1992-1998	219	U	HIV, CD4 & genital HPV: B & 6 mnthly	Clearance of all HPV types (vs Persistent detection of HPV type)	0.48 (0.36,0.63)[2]	CRR F	--		3a,NA
						By CD4 level(cells/μL):					
						≥200	0.29 (0.17,0.48)[10]	HRR F	--		4c,NA
						<200	0.10 (0.04,0.28)[10]	HRR F	--		4c,NA
						Clearance of HPV-16 (vs Persistent detection of HPV-16)	0.56 (0.39,0.81)[2]	CRR F	--		3c,NA
						By CD4 level(cells/μL):					
						≥200	0.27 (0.06,1.24)[10]	HRR F	--		S2b,NA
						<200	0.15 (0.03,0.80)[10]	HRR F	--		S2b,NA
						Clearance of HPV-18 (vs Persistent detection of HPV-18)[3]	No unexp cases	--	--		--
						By CD4 level(cells/μL):					
						≥200	No unexp cases	--	--		--
						<200	No unexp cases	--	--		--
						Clearance of HPV-31 (vs Persistent detection of HPV-31)	1.41 (0.34,5.84)[2]	CRR F	--		3d,NA
						By CD4 level(cells/μL):					
						≥200	0.60 (0.04,8.89)[10]	HRR F	--		S2b,NA
						<200	1.67 (0.11,25.72)[10]	HRR F	--		S2b,NA
						Clearance of HPV-45 (vs Persistent detection of HPV-45)	0.68 (0.52,0.89)[2]	CRR F	--		3d,NA
						By CD4 level(cells/μL):					
						≥200	0.45 (0.08,2.43)[10]	HRR F	--		S2b,NA
						<200	0.21 (0.03,1.55)[10]	HRR F	--		S2b,NA
						(i.e. clearance = time from 1st positive HPV test to the 1st subsequent negative HPV test)					
**USA - The multisite Women's Interagency HIV Study (WIHS): Prospective cohort of HIV positive & high risk women in the Bronx/ Manhattan, Brooklyn, Chicago, Los Angeles, San Francisco & Washington DC**											
**Strickler (2005)(14)**	Mean >13	1994-U	2362	Median of 6 FUP visits	HIV, CD4 & genital HPV: B & 6 mnthly	New incident HPV (vs No new incident HPV) (unit=individual)	2.11 (1.25,3.54)[2]	CRR F	--		2a,NA
**Watts (2005)(15)**	Mean >13	1994-U	2068	U	HIV, CD4 & genital HPV: B & 6 mnthly	New incident HPV (vs No new incident HPV) (unit=type)					
						By CD4 level (cells/μL):					
						<200	4.70 (3.92,5.64) SS	HRR[17]F	5.19 (4.30,6.26) SS	HRR F	4a,4a
						200-500	3.34 (2.79,3.97) SS	HRR[17]F	3.61 (3.01,4.32) SS	HRR F	4a,4a
						>500	2.10 (1.71,2.55) SS	HRR[17]F	2.12 (1.73,2.60) SS	HRR F	4a,4a
						Clearance of any incident HPV type (vs Persistent detection of HPV type) (unit=type)					
						By CD4 level(cells/μL):					
						<200	0.93 (0.77,1.12)[2,10]	HRR[17]F	0.69 (0.57,0.83)[2] SS	HRR F	4c,4c
						200-500	0.83 (0.70,0.99)[2,10]	HRR[17]F	0.83 (0.70,0.98)[2] SS	HRR F	4c,4c
						>500	0.68 (0.57,0.82)[2,10]	HRR[17]F	0.94 (0.78,1.14)[2] SS	HRR F	4c,4c
						(i.e. clearance = time from incident infection to 1st negative HPV test for that type)					
**Viscidi (2003)(16)**	>13	1994-U	2559	U	HIV, CD4 & genital HPV: B & 6 mnthly	Incident HPV-16[11] (vs No new incident HPV)	2.16 (0.99, 4.71)[2]	CRR F	--		2c,NA
**Silverberg (2002)(17)**	>13	1994-U	2798	U	HIV & CD4 & genital HPV: B & 6 mnthly	Incident HPV-6 or HPV-11 (vs No incident HPV-6/HPV-11) (unit=type)	3.1[3]	HRR F	--		--

**D'Souza (2007)(18)**	>13	2004-2005	2798	6 mnths	HIV & CD4 & genital HPV: B & 1 FUP visit at 6 mnths	First incident HPV (vs Remaining HPV-negative)	1.32 (0.45,3.89)[2]	CRR F	--	--	2a,NA
						Clearance of all HPV types (vs Persistent detection of HPV type) (i.e. persistence = type-specific infection still detected at the same anatomic site at the follow-up visit)	0.50 (0.21,1.23)[2]	CRR F	0.53 (0.26,1.05)[2]	OR	3a,3a
**Strickler (2014)(19)**	Mean = 37/33	2004-2005	181	≥2 yrs	HIV & genital HPV: B & 6 mnthly for ≥2 yrs; HPV: B & 6 mnthly for ≥2 yrs	Clearance of all HR-HPV types (vs Persistent detection of HR-HPV type) (i.e. persistence = women testing positive for ≥ 1 HPV type (incident or prevalent) at B who continued to test positive for at least one of these types for 4 semi-annual visits; clearance = positive for ≥1 HPV type at B but negative to all types at the next 6 mnth visit)	0.70 (0.34,1.42)[2]	CRR F	--		3b,NA
**USA - The HIV Epidemiology Research (HER) Study: Prospective cohort study of HIV positive & negative high risk women in Baltimore, Bronx, Providence & Detroit**											
**Ahdieh (2001)(20)**	Mean = 35/32	1993-U	1310	2.5-2.9 yrs	HIV, CD4: B; Genital HPV: B & 6 mnthly	New incident HR-HPV (vs No new incident HR-HPV)	1.82 (1.3,2.7)	HRR F	--		2b,NA
						New incident IR-HPV (vs No new incident IR-HPV)	2.69 (1.99,3.63)[2,4]	HRR F	--		--
						New incident LR-HPV (vs No new incident LR-HPV)	2.67 (2.00,3.60)	HRR F	--		S1a,NA
						Incident HPV-6 (vs Remaining HPV-6 negative)	8.42 (2.1,72.4)[12]	HRR F	--		S1b,NA
						Incident HPV-11 (vs Remaining HPV-11 negative)	3.85 (0.88,16.83)[2,4]	HRR F	--		S1b,NA
						Incident HPV-33 (vs Remaining HPV-33 negative)	3.63 (1.4,11.9)[12]	HRR F	--		2d,NA
						Incident HPV-52 (vs Remaining HPV-52 negative)	4.00 (2.0,9.1)[12]	HRR F	--		2d,NA
						Incident HPV-58 (vs Remaining HPV-58 negative)	3.35 (1.5,8.8)[12]	HRR F	--		2d,NA
**Koshiol (2006)(21)**	Mean = 35	1993-U	801	4.4 yrs	HIV, CD4: B; Genital HPV: B & 6 mnthly	Clearance of any incident or prevalent HR-HPV type (vs Persistent detection of HR-HPV type) (unit=type)	0.60 (0.47,0.76)[2]	HRR F	--		3b,NA
						Clearance of any LR-HPV type (vs Persistent detection of LR-HPV type) (unit=type)	0.63 (0.46,0.84)[2]	HRR F	--		S1c,NA
						Clearance of HPV-16 (vs Persistent detection of HPV-16)	0.70 (0.38,1.31)[2]	HRR F	--		3c,NA
						Clearance of HPV-18 (vs Persistent detection of HPV-18)	0.82 (0.29,2.32)[2]	HRR F	--		3c,NA
						Clearance of any incident HR-HPV type (vs Persistent detection of HR-HPV type) (unit=type)	0.85 (0.64,1.13)[2]	HRR F	--		--
						Clearance of any incident LR-HPV type (vs Persistent detection of LR-HPV type) (unit=type)	0.85 (0.60,1.20)[2]	HRR F	--		--
						Clearance of incident HPV-16 (vs Persistent detection of HPV-16)	0.86 (0.41,1.83)[2]	HRR F	--		--
						Clearance of incident HPV-18 (vs Persistent detection of HPV-18) (i.e. time from 1st positive HPV test at B or during FUP to 1st of 2 consecutive negative tests for same HPV type)	1.19 (0.40,3.54)[2]	HRR F	--		--
**Viscidi (2005)(22)**	U	1993-U	1242	2.5-2.9 yrs (median 10 visits)	HIV, CD4: B; Genital HPV: B & 6 mnthly	Incident HPV-16 (vs Remaining HPV-16 negative)	2.14 (1.26,3.64)[2]	HRR F	--		2c,NA
						Incident HPV-18 (vs Remaining HPV-18 negative)	2.28 (1.30,4.01)[2]	HRR F	--		2c,NA
						Incident HPV-31 (vs Remaining HPV-31 negative)	1.12 (0.66,1.91)[2]	HRR F	--		2d,NA
						Incident HPV-45 (vs Remaining HPV-45 negative)	3.94 (1.88,8.25)[2]	HRR F	--		2d,NA
**USA - New York Cervical Disease Study (NY-CD): Prospective sub cohort of HIV positive & high risk women without SIL part of the NY-CDS in New York, New York**											

**Sun (1997)(23)**	Mean = 35	1991-1993	181	U	HIV, CD4: B; Genital HPV: B & 6 mnthly	Clearance of all HPV types (vs Persistent detection of HPV type)	0.67 (0.56,0.81)[2]	CRR F	--		3a,NA
						By CD4 level (cells/µL):					
						<200	0.79 (0.62,1.00)[2,18]	CRR F	--		4c,NA
						200-499	0.90 (0.76,1.06)[2,18]	CRR F	--		4c,NA
						≥500	0.96 (0.82,1.12)[2,18]	CRR F	--		4c,NA
						Test for trend comparing <500 & ≥500: p=0.001					
						Clearance of all HR-HPV types (vs Persistent detection of HR-HPV type)	0.73 (0.62,0.87)[2]	CRR F	--		3b,NA
						Clearance of all IR-HPV types (vs Persistent detection of IR-HPV type)	0.70 (0.51,0.96)[2]	CRR F	--		--
						Clearance of all LR-HPV types (vs Persistent detection of LR-HPV type)	0.92 (0.72,1.17)[2]	CRR F	--		S1c,NA
						Clearance of all HPV-16/18/33/35/58[13] (vs Persistent detection of any of these types)	0.78 (0.63,0.98)[2]	CRR F	--		--
						Clearance of both HPV-18/45[13] (vs Persistent detection of HPV-18 or HPV-45)	0.62 (0.49,0.77)[2]	CRR F	--		--
						(i.e. persistence = detection of the same HPV types at ≥2 examinations during a period of 3-12 mnths among those in whom HPV was previously detected)					
**USA - The WIHS: Women in a prospective follow-up study prior to WIHS in Brooklyn, New York**											
**Minkoff (1998)(24)**	U	1990-1994	412	10.4/13.5 mnths	HIV, CD4 & genital HPV: B & 6 mnthly	New incident HR-HPV (vs No new incident HR-HPV)	2.64 (1.58, 4.42)[2]	HRR F	--		2b,NA
						By CD4 level(cells/µL):					
						<200	1.76 (0.42,7.38)[2,19]	HRR F	--		4b,NA
						200-499	2.10 (1.09,4.04)[2,19]	HRR F	--		4b,NA
						≥500	3.87 (2.05,7.33)[2,19]	HRR F	--		4b,NA
						Test for trend comparing a 10% change in CD4 level: p=0.04					
						Clearance of all HR-HPV types (vs Persistent detection of HR-HPV type)	0.84 (0.49, 1.43)[2]	HRR F	--		3b,NA
						By CD4 level(cells/µL):					
						<200	0.85 (0.42,1.73)[2,10]	HRR F	--		4d,NA
						200-499	0.77 (0.36,1.67)[2,10]	HRR F	--		4d,NA
						≥500	0.94 (0.37,2.42)[2,10]	HRR F	--		4d,NA
						(i.e. clearance = HR-HPV present at a prior visit not found at a subsequent visit)					
**LATIN AMERICA & THE CARIBBEAN**											
**Brazil - Sao Paulo cohort of men: Prospective follow-up study of male STI clinic attendees in Sao Paulo**											
**Silva (2011)(25)**	Mean = 43/30	2004-2006	102	180 days	HIV: B; Penile HPV: B, after 30, 60, 180 days	Clearance of all HPV types (vs Persistent detection of HPV type)<br>(i.e. persistence = detection of any HPV in ≥2 consecutive specimens during FUP; clearance = failure to detect HPV following a positive test in a previous visit)	0.62 (0.30, 1.29)[2]	CRR M	--		3a,NA
**Brazil - Ribeirao cohort of pregnant women: Small prospective follow-up study of pregnant women in Ribeirao Preto**											
**Jalil (2013)(26)**	Mean = 25	2007-2010	151	9.6/7.8 mnths	HIV: B; Genital HPV: B, 2nd half of pregnancy, after delivery	Clearance of all HPV types (vs Persistent detection of HPV type)<br>(i.e. persistence = ≥2 consecutive positive HPV tests between the 2nd & 3rd visit if positive at B)	1.04 (0.73, 1.47)[2]	CRR F	1.00 (0.60, 1.70)	CRR	3a,3a
**Brazil - Women in a cohort in Belo Horizonte**											

**Ceccato Junior (2016)(27)**	U	2006-2011	163	≥1 yr	HIV: B; Genital HPV: B & FUP	First incident HPV (vs Remaining HPV-negative)	0.96 (0.50, 1.82)[2]	CRR	--		2a,NA
**WESTERN EUROPE**											
**The Netherlands - MSM in a cohort in Amsterdam**											
**Mooij (2016)(28)**	Median = 38-46	2010-2011	705	25 mnths (5 visits)	HIV & anal & penile HPV: B & 6 mnthly	First incident anal HR-HPV (vs Remaining anal HR-HPV-negative) (unit=type)	1.76 (1.40, 2.20)	HRR	1.63 (1.29, 2.06)	HRR	--
						First incident penile HR-HPV (vs Remaining penile HR-HPV-negative) (unit=type)	1.42 (1.04, 1.93)	HRR	1.43 (1.00, 2.07)	HRR	2b,2b
						Incident anal HPV-16 (vs Remaining anal HPV-16-negative)	1.95 (1.18, 3.21)	HRR	1.87 (1.08, 3.23)	HRR	--
						Incident penile HPV-16 (vs Remaining penile HPV-16-negative)	1.36 (0.79, 2.32)	HRR	1.53 (0.85, 2.78)	HRR	2c,2c
						Incident anal HPV-18 (vs Remaining anal HPV-18-negative)	1.52 (0.86, 2.68)	HRR	1.90 (1.01, 3.58)	HRR	--
						Incident penile HPV-18 (vs Remaining penile HPV-18-negative)	1.04 (0.53, 2.03)	HRR	1.27 (0.61, 2.63)	HRR	2c,2c
						Incident anal HPV-31 (vs Remaining anal HPV-31-negative)	2.35 (1.49, 3.70)	HRR	NR		--
						Incident penile HPV-31 (vs Remaining penile HPV-31-negative)	2.03 (1.10, 3.75)	HRR	NR		2d,NA
						Incident anal HPV-33 (vs Remaining anal HPV-33-negative)	1.36 (0.73, 2.52)	HRR	NR		--
						Incident penile HPV-33 (vs Remaining penile HPV-33-negative)	1.16 (0.54, 2.50)	HRR	NR		2d,NA
						Incident anal HPV-45 (vs Remaining anal HPV-45-negative)	1.44 (0.85, 2.42)	HRR	NR		--
						Incident penile HPV-45 (vs Remaining penile HPV-45-negative)	1.26 (0.64, 2.46)	HRR	NR		2d,NA
						Incident anal HPV-52 (vs Remaining anal HPV-52-negative)	1.67 (1.06, 2.62)	HRR	NR		--
						Incident penile HPV-52 (vs Remaining penile HPV-52-negative)	1.87 (0.96, 3.63)	HRR	NR		2d,NA
						Incident anal HPV-58 (vs Remaining anal HPV-58-negative)	2.41 (1.43, 4.05)	HRR	NR		--
						Incident penile HPV-58 (vs Remaining penile HPV-58-negative)	1.23 (0.43, 3.54)	HRR	NR		2d,NA
						Clearance of any anal HR-HPV type (vs Persistent detection of anal HR-HPV type) (unit=type)	0.67 (0.55, 0.82)	HRR	0.72 (0.58, 0.89)	HRR	--
						Clearance of any penile HR-HPV type (vs Persistent detection of penile HR-HPV type) (unit=type)	1.33 (1.02, 1.73)	HRR	1.28 (0.96, 1.71)	HRR	3b,3b
						Clearance of anal HPV-16 (vs Persistent detection of anal HPV-16)	0.93 (0.60, 1.44)	HRR	0.94 (0.56, 1.59)	HRR	--
						Clearance of penile HPV-16 (vs Persistent detection of penile HPV-16)	1.86 (1.04, 3.32)	HRR	2.25 (1.12, 4.56)	HRR	3c,3c
						Clearance of anal HPV-18 (vs Persistent detection of anal HPV-18)	0.60 (0.35, 1.02)	HRR	0.63 (0.36, 1.09)	HRR	--
						Clearance of penile HPV-18 (vs Persistent detection of penile HPV-18)	2.01 (1.01, 4.03)	HRR	1.87 (0.86, 4.05)	HRR	3c,3c
						Clearance of anal HPV-31 (vs Persistent detection of anal HPV-31)	0.47 (0.31, 0.71)	HRR	NR		--
						Clearance of penile HPV-31 (vs Persistent detection of penile HPV-31)	1.63 (0.80, 3.31)	HRR	NR		3d,NA
						Clearance of anal HPV-33 (vs Persistent detection of anal HPV-33)	0.66 (0.38, 1.13)	HRR	NR		--
						Clearance of penile HPV-33 (vs Persistent detection of penile HPV-33)	2.53 (1.15, 5.56)	HRR	NR		3d,NA
						Clearance of anal HPV-45 (vs Persistent detection of anal HPV-45)	0.58 (0.36, 0.95)	HRR	NR		--
						Clearance of penile HPV-45 (vs Persistent detection of penile HPV-45)	1.00 (0.50, 1.98)	HRR	NR		3d,NA
						Clearance of anal HPV-52 (vs Persistent detection of anal HPV-52)	0.62 (0.41, 0.94)	HRR	NR		--
						Clearance of penile HPV-52 (vs Persistent detection of penile HPV-52)	1.27 (0.69, 2.35)	HRR	NR		3d,NA
						Clearance of anal HPV-58 (vs Persistent detection of anal HPV-58)	1.05 (0.51, 2.17)	HRR	NR		--
						Clearance of penile HPV-58 (vs Persistent detection of penile HPV-58)	1.70 (0.46, 6.33)	HRR	NR		3d,NA

						First incident anal HR-HPV (vs Remaining anal HR-HPV-negative) (unit=type)					
						By CD4 level(cells/µL):					
						<200	2.34 (1.55, 3.53)[21]	HRR	1.79 (1.20, 2.68)[21]	HRR	--
						200-350	1.76 (1.33, 2.34)[21]	HRR	1.64 (1.26, 2.15)[21]	HRR	--
						>350	1.36 (0.95, 1.95)[21]	HRR	1.43 (1.00, 2.06)[21]	HRR	--
						First incident penile HR-HPV (vs Remaining penile HR-HPV-negative) (unit=type)					
						By CD4 level(cells/µL):					
						<200	1.65 (0.85, 3.26)[21]	HRR	1.59 (0.74, 3.38)[21]	HRR	4b,4b
						200-350	1.41 (0.89, 2.24)[21]	HRR	1.43 (0.85, 2.42)[21]	HRR	4b,4b
						>350	1.26 (0.77, 2.06)[21]	HRR	1.28 (0.75, 2.28)[21]	HRR	4b,4b
						Incident anal HPV-16 (vs Remaining anal HPV-16-negative)					
						By CD4 level(cells/µL):					
						<200	1.58 (0.62, 4.05)[21]	HRR	1.32 (0.54, 3.43)[21]	HRR	--
						200-350	2.27 (1.21, 4.26)[21]	HRR	2.13 (1.10, 4.12)[21]	HRR	--
						>350	1.82 (0.87, 3.79)[21]	HRR	2.03 (0.90, 4.57)[21]	HRR	--
						Incident penile HPV-16 (vs Remaining penile HPV-16-negative)					
						By CD4 level(cells/µL):					
						<200	1.43 (0.53, 3.80)[21]	HRR	Too few outcomes		S2a,NA
						200-350	1.37 (0.68, 2.79)[21]	HRR	Too few outcomes		S2a,NA
						>350	1.14 (0.50, 2.62)[21]	HRR	Too few outcomes		S2a,NA
						Incident anal HPV-18 (vs Remaining anal HPV-18-negative)					
						By CD4 level(cells/µL):					
						<200	1.52 (0.55, 4.23)[21]	HRR	1.70 (0.61, 4.77)[21]	HRR	--
						200-350	1.36 (0.62, 3.00)[21]	HRR	1.68 (0.74, 3.80)[21]	HRR	--
						>350	1.76 (0.80, 3.87)[21]	HRR	2.53 (1.04, 6.16)[21]	HRR	--
						Incident penile HPV-18 (vs Remaining penile HPV-18-negative)					
						By CD4 level(cells/µL):					
						<200	2.02 (0.76, 5.34)[21]	HRR	Too few outcomes		S2a,NA
						200-350	1.07 (0.43, 2.65)[21]	HRR	Too few outcomes		S2a,NA
						>350	0.25 (0.03, 1.87)[21]	HRR	Too few outcomes		S2a,NA
						Clearance of any anal HR-HPV type (vs Persistent detection of anal HR-HPV type) (unit=type)					
						By CD4 level(cells/µL):					
						<200	0.69 (0.46, 1.05)[21]	HRR	0.76 (0.49, 1.17)[21]	HRR	--
						200-350	0.66 (0.51, 0.84)[21]	HRR	0.70 (0.53, 0.92)[21]	HRR	--
						>350	0.69 (0.50, 0.95)[21]	HRR	0.72 (0.51, 1.01)[21]	HRR	--
						Clearance of any penile HR-HPV type (vs Persistent detection of penile HR-HPV type) (unit=type)					
						By CD4 level(cells/µL):					
						<200	1.04 (0.66, 1.64)[21]	HRR	0.99 (0.63, 1.56)[21]	HRR	4d,4d
						200-350	1.49 (1.09, 2.04)[21]	HRR	1.49 (1.07, 2.08)[21]	HRR	4d,4d
						>350	1.33 (0.92, 1.93)[21]	HRR	1.34 (0.90, 2.02)[21]	HRR	4d,4d
						Clearance of anal HPV-16 (vs Persistent detection of anal HPV-16)					
						By CD4 level(cells/µL):					
						<200	0.98 (0.44, 2.18)[21]	HRR	0.93 (0.39, 2.21)[21]	HRR	--
						200-350	0.91 (0.53, 1.57)[21]	HRR	0.98 (0.53, 1.83)[21]	HRR	--
						>350	0.98 (0.49, 1.96)[21]	HRR	1.02 (0.48, 2.21)[21]	HRR	--

						Clearance of penile HPV-16 (vs Persistent detection of penile HPV-16)					
						By CD4 level(cells/µL):					
						<200	1.58 (0.58, 4.29)[21]	HRR	Too few outcomes		S2b,NA
						200-350	1.44 (0.72, 2.85)[21]	HRR	Too few outcomes		S2b,NA
						>350	1.76 (0.75, 4.15)[21]	HRR	Too few outcomes		S2b,NA
						Clearance of anal HPV-18 (vs Persistent detection of anal HPV-18)					
						By CD4 level(cells/µL):					
						<200	0.62 (0.25, 1.56)[21]	HRR	0.68 (0.27, 1.74)[21]	HRR	S2b,NA
						200-350	0.44 (0.21, 0.95)[21]	HRR	0.43 (0.19, 0.97)[21]	HRR	S2b,NA
						>350	0.96 (0.46, 1.99)[21]	HRR	0.80 (0.42, 1.74)[21]	HRR	S2b,NA
						Clearance of penile HPV-18 (vs Persistent detection of penile HPV-18)					
						By CD4 level(cells/µL):					
						<200	Zero value cell		Zero value cell		--
						200-350	Zero value cell		Zero value cell		--
						>350	Zero value cell		Zero value cell		--
						(i.e. clearance = 1 positive HPV test followed by 2 consecutive negative tests)					
**The Netherlands - The Rotterdam MSM cohort: Subsample of a prospective cohort of MSM in Rotterdam**											
**van der Snoek (2005)(29)**	Median = 42.5	2000-2003	213	21 mnths (visits 3-6)[14]	HIV: 3rd visit?; Perianal HPV: at 3rd & 6th visits occurring every 6 mnths	Incident HPV-6 (vs Remaining HIV-6 negative)	1.6[2,3]	HRR M	--		--
						Incident HPV-11 (vs Remaining HIV-11 negative)	0.0[2,3]	HRR M	--		--
						Incident HPV-16 (vs Remaining HIV-16 negative)	5.2[2,3]	HRR M	--		--
						Incident HPV-18 (vs Remaining HIV-18 negative)	3.8[2,3]	HRR M	--		--
						Incident HPV-31 (vs Remaining HIV-31 negative)	9.4[2,3]	HRR M	--		--
						Incident HPV-33 (vs Remaining HIV-33 negative)	0.0[2,3]	HRR M	--		--
						Incident HPV-52 (vs Remaining HIV-52 negative)	7.4[2,3]	HRR M	--		--
						Clearance of HPV-6 (vs Persistent detection of HPV-6)	NR	--	--		--
						Clearance of HPV-11 (vs Persistent detection of HPV-11)	NR	--	--		--
						Clearance of HPV-16 (vs Persistent detection of HPV-16)	NR	--	--		--
						Clearance of HPV-18 (vs Persistent detection of HPV-18)	NR	--	--		--
						Clearance of HPV-31 (vs Persistent detection of HPV-31)	NR/SS	--	--		--
						Clearance of HPV-33 (vs Persistent detection of HPV-33)	NR	--	--		--
						Clearance of HPV-52 (vs Persistent detection of HPV-52)	NR	--	--		--
						(i.e. persistence = type detected at both visits)					
**Italy - Naples cohort of gynaecological attendees: Subsample of a prospective cohort of HIV+ & HIV- women attending gynaecological clinic in Naples**											
**Tornesello (2008)(30)**	Mean = 32	2001-2004	227	36 mnths	HIV: B; Cervical HPV: 6 mnthly (≥3 visits)	First incident HPV (vs Remaining HPV-negative)	1.82 (0.51,6.53)[2]	CRR F	--		2a,NA
						Clearance of all HPV types (vs Persistent detection of HPV type)	0.51 (0.19,1.38)[2]	CRR F	--		3a,NA
						(i.e. clearance = having ≥1 negative HPV test among those HPV-positive at B)					
**Italy - The DIANAIDS project: Subsample of a multi-centre prospective study of women from exposed to the same risk factors (higher risk)**											
**Branca (2003)(31)**	Mean = 33	1997-1999	120	14.1 mnths	HIV & cervical HPV: B & the FUP visits	First incident HPV (vs Remaining HPV-negative)	8.80 (1.20,64.61)[2]	CRR F	--		2a,NA
						Clearance of all HPV types (vs Persistent detection of HPV type)	0.33 (0.16,0.67)[2]	CRR F	--		3a,NA
						(i.e. clearance = positive test at B followed by subsequent negative HPV test)					
**SUB-SAHARAN AFRICA**											
**South Africa - SA heterosexual couple HPV study: Prospective cohort of men & women in heterosexual seroconcordant or serodiscordant partnerships by HIV status, Cape Town**											

**Mbulaw**	Mean:	U	972	≤24	HIV,	New incident HPV (vs No new incident HPV) (unit=type)	1.96 (1.63,2.37)[2]	HRR M	2.00 (1.49,2.69)	HRR M	--
**a**	men = 38			mnths	CD4: B;		2.93 (2.29,3.74)[2]	HRR F	2.98 (2.07,4.29)	HRR F	--
**(2012)(3**	&				Cervical	By CD4 level(cells/μL):					
**2)**	women =				& penile	≥350	2.15 (1.71,2.70)[2,10]	HRR M	--		--
	35				HPV: 6		2.86 (2.15,3.80)[2,10]	HRR F	--		4a,NA
					mnthly	<350	1.78 (1.38,2.30)[2,10]	HRR M	--		--
					(≤4		2.99 (2.26,3.95)[2,10]	HRR F	--		4a,NA
					visits)	First incident HPV (vs Remaining HPV-negative) (unit=individual)	1.55 (0.87,2.78)[2]	HRR M	--		--
							2.09 (1.24,3.52)[2]	HRR F	--		2a,NA
						First incident HR-HPV (vs Remaining HR-HPV-negative)	2.50 (1.48,4.22)[2]	HRR M	--		--
						(unit=individual)	3.19 (1.71,5.93)[2]	HRR F	--		2b,NA
						First incident LR-HPV (vs Remaining LR-HPV-negative) (unit=individual)	1.52 (0.87,2.66)[2]	HRR M	--		--
							2.48 (1.52,4.07)[2]	HRR F	--		S1a,NA
						Clearance of any HPV type (vs Persistent detection of HPV type)	0.75 (0.63,0.90)[2]	HRR M	0.71 (0.55,0.93)	HRR M	--
						(unit=type)	0.50 (0.40,0.63)[2]	HRR F	0.46 (0.34,0.62)	HRR F	3a,3a
						By CD4 level(cells/μL):	0.76 (0.60,0.97)[2,10]	HRR M	--		--
						≥350	0.58 (0.44,0.76)[2,10]	HRR F	--		4c,NA
							0.75 (0.60,0.92)[2,10]	HRR M	--		--
						<350	0.45 (0.35,0.58)[2,10]	HRR F	--		4c,NA
						(i.e. clearance = 1st negative test for type present in individual at B)					
**Uganda - Rakai male circumcision trials: Prospective studies of men enrolled in part of a trial of circumcision in Rakai Districts**											
**Tobian**	Range =	U	999	14	HIV &	New incident HR-HPV (vs No new incident HR-HPV) (unit=visit)	2.02 (1.67,2.44)[2]	HRR M	--		--
**(2012)(3**	15-49			mnths	penile	New incident HR-HPV (vs No new incident HR-HPV) (unit=type)	2.35 (2.03,2.72)	HRR M	--		2b,NA
**3)**					HPV: B &	Incident HPV-16 (vs Remaining HIV-16 negative)	2.46 (1.52,4.00)	HRR M	--		2c,NA
					at 6, 12	Incident HPV-18 (vs Remaining HIV-18 negative)	2.15 (1.27,3.64)	HRR M	--		2c,NA
					& 24	Incident HPV-31 (vs Remaining HIV-31 negative)	3.59 (1.87,6.90)	HRR M	--		2d,NA
					mnths	Incident HPV-33 (vs Remaining HIV-33 negative)	3.20 (1.63,6.28)	HRR M	--		2d,NA
						Incident HPV-45 (vs Remaining HIV-45 negative)	4.40 (2.30,8.39)	HRR M	--		2d,NA
						Incident HPV-52 (vs Remaining HIV-52 negative)	2.10 (1.21,3.66)	HRR M	--		2d,NA
						Incident HPV-58 (vs Remaining HIV-58 negative)	2.03 (1.23,3.33)	HRR M	--		2d,NA
						1 new incident HR-HPV (vs No new incident HR-HPV) (unit=visit)	1.99 (1.70,2.32)[2]	CRR M	--		--
						≥2 new incident HR-HPV (vs No new incident HR-HPV) (unit=visit)	2.97 (2.15,4.10)	HRR M	--		--
						Clearance of any HR-HPV type (Persistent detection of HR-HPV type) (unit=type)	0.67 (0.59,0.77)	HRR M	--		3b,NA
						Clearance of HPV-16 (Persistent detection of HPV-16)	0.42 (0.27,0.65)	HRR M	--		3c,NA
						Clearance of HPV-18 (Persistent detection of HPV-18)	0.69 (0.43,1.11)	HRR M	--		3c,NA
						Clearance of HPV-31 (Persistent detection of HPV-31)	0.52 (0.30,0.89)	HRR M	--		3d,NA
						Clearance of HPV-33 (Persistent detection of HPV-33)	0.74 (0.43,1.27)	HRR M	--		3d,NA
						Clearance of HPV-45 (Persistent detection of HPV-45)	0.62 (0.40,0.97)	HRR M	--		3d,NA
						Clearance of HPV-52 (Persistent detection of HPV-52)	0.55 (0.33,0.93)[2,4]	HRR M	--		3d,NA
						Clearance of HPV-58 (Persistent detection of HPV-58)	0.92 (0.60,1.42)	HRR M	--		3d,NA
						(i.e. clearance = 1st negative result for a type in individual previously positive for that type)					
**Grabow**	Range =	U	959	1.2 yrs	HIV &	Clearance of any HR-HPV type (type) (Persistent detection of HR-HPV	--		0.44 (0.28, 0.68)[2]	CRR M	NA,3b
**ski**	15-49				penile	type)					
**(2014)(3**					HPV: B &	(i.e. clearance = 1st negative test for a type in someone previously					
**4)**					at 6, 12	testing positive for that type)					

					& 24 mnths					
**South Africa - Women in a cohort in Cape Town**										
**Adler (2015)(35)**	Mean = 19	2012-2014	43	12 mnths	HIV: B; Genital HPV: B & 6 mnthly	Clearance of all HR-HPV types (vs Persistent detection of any HR-HPV type)	0.72 (0.56, 0.93)[2]	CRR	--		3b,NA
						Clearance of HPV-16 or HPV-18 (vs Persistent detection of HPV-16 & HPV-18)	0.63 (0.37, 1.07)[2]	CRR	--		
						Clearance of any non-vaccine HR-HPV type (vs Persistent detection of all non-vaccine HR-HPV types)<br>(i.e. persistence = HPV-positive in any sequential pair of specimens, or any pair of specimens collected 12 mnths apart[22]	0.76 (0.56, 1.01)[2]	CRR	--		
**Uganda - The Kampala teenager women study: Prospective cohort of young women attending health clinic in Kampala**										
**Banura (2010)(36)**	Mean = 20	2002-2006	334	18.5 mnths	HIV: B; Cervical HPV: B, ≥1 visit ≥6 mnthly	First incident HPV (vs Remaining HPV-negative)	--		2.8 (0.9, 8.3)	CRR F	NA,2a
						Clearance of all HPV types (vs Persistent detection of HPV type)<br>(i.e. clearance = all HPV types at B subsequently cleared)	--		0.3 (0.1, 0.8)	CRR F	NA,3a
**Uganda - The Kampala teenager women study: Prospective cohort of young pregnant women attending antenatal clinics in Kampala**										
**Banura (2008)(37)**	<25	2004	334	During pregnancy	HIV: B; Cervical HPV: B & each visit (3rd trimester, &/or after delivery)	New incident HPV (vs No new incident HPV)	1.23 (0.80,1.89)[2]	CRR F	1.5 (0.6, 3.4)	OR F	2a,2a
						Clearance of any HPV type (vs Persistent detection of HPV type) (unit=type)<br>(i.e. clearance = negative test at the final visit of a specific HPV type present at B)	0.77 (0.58,1.01)[2]	CRR F	0.5 (0.3, 0.9)	OR F	3a,3a
**Zimbabwe - The multisite (Uganda, Zimbabwe, Thailand) Hormonal contraception (HC) cohort study (MHCC): Subsample of prospective cohort of general population female seeking reproductive & general health care clinic**										
**Nowak (2011)(38)**	Mean = 25	1999-2004	454	15-24 mnths	HIV: B; Cervical HPV: B, 3 mnthly	1 new incident HR-HPV type (vs No new incident HR-HPV)[16]	2.8 (1.4, 5.5)	OR F	2.5 (1.2,5.1)	OR F	--
						≥2 new incident HR-HPV types (vs No new incident HR-HPV)[16]	4.6 (2.3, 9.3)	OR F	4.5 (2.2,9.3)	OR F	--
**Uganda - The ongoing Rakai Community Cohort Study (RCCS): Subsample of women from the RCCS prospective cohort study**										
**Safaeian (2008)(39)**	Mean = 28	1998-U	1055	~3 yrs	HIV: B; Vaginal HPV: B, each visit (median 3)	New incident HR-HPV (individual) (vs No new incident HR-HPV)	2.48 (1.79,3.43)	HRR F	2.04 (1.43,2.91)	HRR F	2b,2b
						Incident HPV-16 (vs Remaining HPV-16 negative)	2.71 (1.13,6.06)[15]	HRR F	3.09 (1.39,6.85)	HRR F	2c,2c
						Incident HPV-18 (vs Remaining HPV-18 negative)	3.23 (1.06,9.13)[15]	HRR F	3.19 (1.17,8.71)	HRR F	2c,2c
						Incident HPV-31 (vs Remaining HPV-31 negative)	0.60 (0.01,4.09)[15]	HRR F	0.56 (0.07,4.60)	HRR F	2d,2d
						Incident HPV-33 (vs Remaining HPV-33 negative)	0.74 (0.08,3.22)[15]	HRR F	0.51 (0.12,2.30)	HRR F	2d,2d
						Incident HPV-45 (vs Remaining HPV-45 negative)	3.58 (1.35,9.04)[15]	HRR F	3.41 (1.40,8.29)	HRR F	2d,2d
						Incident HPV-52 (vs Remaining HPV-52 negative)	1.47 (0.33,4.24)[15]	HRR F	1.17 (0.38,3.60)	HRR F	2d,2d
						Incident HPV-58 (vs Remaining HPV-58 negative)	5.99 (1.72,21.56)[15]	HRR F	6.15 (1.94,19.55)	HRR F	2d,2d
						Clearance of any HR-HPV type (vs Persistent detection of all HR-HPV types)[3] (unit=individual)	0.70 (0.56,0.87)	HRR F	0.75 (0.59,0.96)	HRR F	--
						Clearance of any HR-HPV type (vs Persistent detection of HR-HPV type) (unit=type)	0.70 (0.56,0.88)[2,15]	HRR F	0.71 (0.56,0.91)[2]	HRR F	3b,3b
						Clearance of HPV-16 (vs Persistent detection of HPV-16)	0.57 (0.27,1.23)[2,15]	HRR F	0.62 (0.28,1.35)[2]	HRR F	3c,3c
						Clearance of HPV-18 (vs Persistent detection of HPV-18)	0.91 (0.62,1.33)[2,15]	HRR F	0.82 (0.56,1.18)[2]	HRR F	3c,3c

						Clearance of HPV-31 (vs Persistent detection of HPV-31)<br>Clearance of HPV-33 (vs Persistent detection of HPV-33)<br>Clearance of HPV-45 (vs Persistent detection of HPV-45)<br>Clearance of HPV-52 (vs Persistent detection of HPV-52)<br>Clearance of HPV-58 (vs Persistent detection of HPV-58)<br>(i.e. clearance = 1st negative result for a type in individuals previously testing positive for that type)	0.25 (0.03,2.44)[2,15]<br>0.43 (0.15,1.27)[2,15]<br>0.53 (0.25,1.16)[2,15]<br>0.60 (0.32,1.11)[2,15]<br>0.64 (0.31,1.30)[2,15]	HRR F<br>HRR F<br>HRR F<br>HRR F<br>HRR F	0.39 (0.09,1.67)[2]<br>0.25 (0.12,0.53)[2]<br>0.75 (0.32,1.75)[2]<br>0.59 (0.31,1.11)[2]<br>0.57 (0.29,1.10)[2]	HRR F<br>HRR F<br>HRR F<br>HRR F<br>HRR F	3d,3d<br>3d,3d<br>3d,3d<br>3d,3d<br>3d,3d
**Senegal - Senegalese cohort of women: Prospective cohort of female infectious disease clinic attendees & FSWs attending STI clinics in Dakar & M'Bour**											
**Rowhani-Rahbar (2007)(40)**	Mean = 31	1994-1998	614	2.1 yrs	HIV: B; CD4 & cervical HPV: B, each visit (≥2, mean 5)	Clearance of all HPV types (vs Persistent detection of HPV type)<br>Clearance of all HPV types (women with HR-HPV) (vs Persistent detection of HPV type)<br>Clearance of all LR-HPV/untyped HPV (vs Persistent detection of LR-HPV type/untyped HPV)<br>Clearance of HPV-16 (vs Persistent detection of HPV-16)<br>Clearance of HPV-18 (vs Persistent detection of HPV-18)<br>(i.e. clearance = 2 consecutive HPV-negative tests during follow-up in those positive at B)	0.30 (0.22,0.40)<br>--<br>--<br>0.57 (0.32,1.02)<br>0.42 (0.19,0.95)	HRR F<br><br><br>HRR F<br>HRR F	0.31 (0.21,0.45)<br>0.23 (0.13,0.41)<br>0.37 (0.23,0.61)<br>NR<br>NR	HRR F<br>HRR F<br>HRR F<br>--<br>--	3b, 3b<br>--<br>--<br>3c,NA<br>3c,NA
**Malawi - Urban cohort study in Malawi: Prospective cohort of pregnant urban women attending antenatal clinics**											
**Miotti (1996)(41)**	U	1991-1992	124	342 mnths	HIV: B; Genital HPV: B, 12 mnths	First incident HPV (vs Remaining HPV-negative)<br>Clearance of all HPV types (vs Persistent detection of HPV type)<br>(i.e. clearance = no subsequent HPV DNA detection in those positive at B; persistence = HPV DNA detected at the follow-up visit in those positive at B)	1.07 (0.65 1.76)[2]<br>0.29 (0.15,0.58)[2]	CRR F<br>CRR F	--<br>--		2a,NA<br>3a,NA
**EAST ASIA & PACIFIC**											
**Thailand - Bangkok prospective monitoring studies (BPMS): Prospective study of MSM who visited Men's Health Clinic at the Thai Red Cross AIDS Research Centre in Bangkok**											

**Phanuphak, Phanuphak (2013a, 2013)(42, 43)**	Mean = 28	2009-2012	246	12 mnths	HIV: B; Anal HPV: B, annually & bi-annually (1st 120 men only)	New incident HR-HPV (vs No new incident HR-HPV) (unit=individual)	2.30 (1.24,4.26)[2,4]	HRR M	NR	--	2b,NA
						Incident HPV-16 (vs Remaining HPV-16 negative)	2.65 (0.94,7.43)[2,4]	HRR M	NR	--	2c,NA
						Incident HPV-18 (vs Remaining HPV-18 negative)	1.40 (0.46,4.29)[2,4]	HRR M	NR	--	2c,NA
						Incident HPV-31 (vs Remaining HPV-31 negative)	3.49 (0.39,31.23)[2,4]	HRR M	NR	--	2d,NA
						Incident HPV-33 (vs Remaining HPV-33 negative)	4.51 (0.53,38.58)[2,4]	HRR M	NR	--	2d,NA
						Incident HPV-45 (vs Remaining HPV-45 negative)	1.83 (0.46,7.31)[2,4]	HRR M	NR	--	2d,NA
						Incident HPV-52 (vs Remaining HPV-52 negative)	2.45 (0.65,9.23)[2,4]	HRR M	NR	--	2d,NA
						Incident HPV-58 (vs Remaining HPV-58 negative)	3.43 (0.96,12.28)[2,4]	HRR M	NR	--	2d,NA
						Incident HPV-6 (vs Remaining HPV-6 negative)	4.64[3]	HRR M	NR	--	--
						Incident HPV-11 (vs Remaining HPV-11 negative)	NR/NS	--	NR	--	--
						Clearance of any HR-HPV type (vs Persistent detection of all HR-HPV types) (unit=individual)	0.75 (0.37,1.54)[2,4]	HRR M	NR	--	--
						Clearance of all HR-HPV types (vs Persistent detection of HR-HPV type) (unit=type)	0.24 (0.12,0.49)[2,4]	HRR M	0.22 (0.11, 0.47)[2]	HRR M	3b,3b
						Clearance of HPV-16 (vs Persistent detection of HPV-16)	0.51 (0.21,1.24)[2,4]	HRR M	NR	--	3c,NA
						Clearance of HPV-18 (vs Persistent detection of HPV-18)	1.10 (0.32,3.77)[2,4]	HRR M	NR	--	3c,NA
						Clearance of HPV-31 (vs Persistent detection of HPV-31)	No unexp individ's	--	NR	--	--
						Clearance of HPV-33 (vs Persistent detection of HPV-33)	No unexp individ's	--	NR	--	--
						Clearance of HPV-45 (vs Persistent detection of HPV-45)	1.52 (0.19,12.17)[2,4]	HRR M	NR	--	3d,NA
						Clearance of HPV-52 (vs Persistent detection of HPV-52)	1.83 (0.41,8.27)[2,4]	HRR M	NR	--	3d,NA
						Clearance of HPV-58 (vs Persistent detection of HPV-58)	No unexp cases[2,4]	--	NR	--	--
						Clearance of HPV-6 (vs Persistent detection of HPV-6)	NR	--	NR	--	--
						Clearance of HPV-11 (vs Persistent detection of HPV-11) (i.e. persistence = having the same specific HPV type at 2 consecutive visits)	NR	--	NR	--	--

Key: Studies are grouped by geographical region. For each region, studies carried out among both females and males are listed first, followed by those studies among males only, and then those studies among females only. Studies are then listed in order of study year, with the most recent listed first. Unit of infection is individuals, measured over the entire study period, unless otherwise specified as follows (unit=individual may still be specified for clarity where results presented for more than one unit of infection): visit – measurement done for individuals treating each interval between study visits separately; type – incidence/clearance of individual HPV types tracked. Individual as the unit of infection is equivalent to type as the unit of infection for incidence/clearance of individual HPV types. More than one mean/median age or follow-up duration is given where this was only presented for study subgroups. HR-HPV – high-risk HPV; LR-HPV – low-risk HPV; IR-HPV – intermediate-risk HPV; CI – confidence interval; HRR – hazard rate ratio; CRR – cumulative risk ratio; OR – odds ratio; U – unknown; NR – estimate not reported; NS – association reported as being not (statistically) significant; SS – association reported as being statistically significant; MSM – men who have sex with men; FSWs – female sex workers; FUP – follow-up; B – baseline. [1]Largest sample size of listed associations given: where this could not be calculated precisely because of partially overlapping subgroups or because these data were not available then the best estimate for the sample size was used; [2]Estimate and/or 95%CIs calculated from data given in the publication; [3]95%CIs not presented or able to be calculated; [4]Apparent error in published data, data corrected; [5]These two estimates are in fact different to 3 decimal places; [6]p=0.03; [7]p=0.2; [8]p=0.06; [9]p<0.01; [10]No reported test for trend; [11]Not clear if the totals include those HPV-16 positive at baseline; [12]Calculation of upper 95%CI from SE in meta-analysis gives results that differ to results presented in publication; [13]Not all types necessarily present at baseline; [14]Four HIV seroconversions but seroconversions seem to have occurred before visit 3 with no other seroconversions between visits 3 and 6; [15]Calculation of 95%CIs from SE in meta-analysis gives results that differ more than might be expected to results presented in publication; [16]Results at visit t+2 extracted (6 months after index visit); [17]Reported as odds ratios, but seem to be hazard ratios; [18]p=0.001; [19]p=0.04; [20]Female and male estimates correlated as partnership study; [21]Test for trend not statistically significant; [22]Results at 6 months also presented although some clearance numbers seem to be higher at 6 months than at 12 months.

Table S2. Summary of the 15 publications identified reporting on the effect of HPV infection or clearance on HIV acquisition (review 2)

Source	Age (years)	Study period	Sample size[12]	Mean follow-up	Testing frequency	Exposure (vs Comparison Group)	Outcome	Study estimate (95%CI)				Fig.
								Crude		Adjusted		
**NORTH AMERICA**												
**USA - MSM in a trial EXPLORE of a behavioural intervention in Boston, Denver, New York & San Francisco**												
**Chin-Hong (2009)(44)**	Range = 16-≥36	2001-2004?	1409	Range = 12-48 mnths	HPV: B & 6 mnthly; HIV B & 6 mnthly	1 prevalent HPV type (vs HPV-negative)<br>≥2 prevalent HPV types (vs HPV-negative)	Incident HIV	2.8 (1.04,7.4)<br>3.6 (1.5,8.4)	HRR M<br>HRR M	2.0 (0.61,6.5)<br>3.5 (1.2,10.6)	HRR M<br>HRR M	--<br>--
**SUB-SAHARAN AFRICA**												
**South Africa - Mostly heterosexual men in a trial ANRS-1265 of circumcision against HIV in Orange Farm township**												
**Auvert (2010)(45)**	Range = 18-24	2004-2006?	1683	21 mnths	HPV: at 21 mnth FUP visit; HIV: at 3, 12 & 21 mnths – high possibility of reverse causality	Prevalent HPV (vs HPV-negative)<br>Prevalent HR-HPV (vs HR-HPV-negative)<br>Prevalent LR-HPV (vs LR-HPV-negative)<br>Increase in HIV risk with number of HR-HPV types	Incident HIV	5.45 (2.72,10.9)<br>5.41 (2.72,10.78)<br>--<br>--	HRR M<br>HRR M	4.55 (2.23,9.28)<br>3.76 (1.83,7.73)<br>1.13 (0.40,3.16)[8]<br>1.55 (1.12,2.14)	HRR M<br>HRR M<br>HRR M<br>HRR M	5a,5a<br>--<br>--<br>--
**Kenya - Mostly heterosexual men in a trial of circumcision against HIV in Kisumu**												
**Smith (2010)(46)**	Mean = 20 (range 17-28)	2002-2006	2168	Range = 24-42 mnths	HPV: B; HIV: at 1, 3, 6, 12, 18, 24 mnths (unclear after 24 mnths)(47)	Prevalent HPV (glans only) (vs HPV-negative)[2]<br>Prevalent HR-HPV (vs HR-HPV-negative)<br>Prevalent LR-HPV (vs LR-HPV-negative)<br>1 prevalent HPV type (vs HPV-negative)<br>≥2 prevalent HPV types (vs HPV-negative)<br>≥2 prevalent HPV types (vs ≤1 prevalent HPV types)<br>Prevalent HPV-16 or HPV-18 (vs HPV-negative)<br>Prevalent HPV without HPV-16 or HPV-18 (vs HPV-negative)<br>Prevalent HPV-6, HPV-11, HPV-16 or HPV-18 (vs HPV-negative)<br>Prevalent HPV without HPV-6, HPV-11, HPV-16 or HPV-18 (vs HPV-negative)	Incident HIV	1.93 (1.17,3.19)[1]<br>1.41 (0.86,2.30)[1]<br>1.42 (0.78,2.59)[1]<br>1.42 (0.74,2.74)[1]<br>1.94 (1.12,3.37)[1]<br>1.72 (1.05,2.82)[1]<br>2.49 (1.31,4.70)[1]<br>1.46 (0.83,2.54)[1]<br>1.98 (1.05,3.70)[1]<br>1.59 (0.90,2.79)[1]	CRR M<br>CRR M<br>CRR M<br>CRR M<br>CRR M<br>CRR M<br>CRR M<br>CRR M<br>CRR M<br>CRR M	1.8 (1.1,2.9)<br>--<br>--<br>1.3 (0.7,2.6)<br>1.8 (1.0,3.1)<br>--<br>2.4 (1.2,4.6)<br>1.3 (0.8,2.4)<br>1.9 (1.0,3.7)<br>1.5 (0.8,2.6)	HRR M<br><br><br>HRR M<br>HRR M<br><br>HRR M<br>HRR M<br>HRR M<br>HRR M	--<br>--<br>--<br>--<br>--<br>--<br>--<br>--<br>--<br>--
**Smith/Houlihan (2010/2012)(46, 48)**	Mean = 20 (range 17-28)	2002-2006	2168	Range = 24-42 mnths	HPV: B; HIV: at 1, 3, 6, 12, 18, 24 mnths (unclear after 24 mnths(47)	Prevalent HPV-16 or HPV-18 (vs HPV-16 & HPV-18 negative)<br>Prevalent HPV-6, HPV-11, HPV-16 or HPV-18 (vs HPV-6, HPV-11, HPV-16 & HPV-18 negative)<br>Prevalent HPV-6, HPV-11, HPV-16, HPV-18, HPV-31, HPV-33, HPV-45, HPV-52 or HPV-58 (vs HPV-6, HPV-11, HPV-16, HPV-18, HPV-31, HPV-33, HPV-45, HPV-52 & HPV-58 negative)	Incident HIV	2.08 (1.18,3.67)[1]<br>1.60 (0.92,2.79)[1]<br>--	CRR M<br>CRR M	1.8 (0.9,3.4)[6]<br>1.3 (0.7,2.5)[6]<br>1.4 (0.8,2.7)[6]	HRR M<br>HRR M<br>HRR M	--<br>--<br>--
**Rositch (2014)(49)**	Mean = 20 (range 19-22)	2002-2007	2519	24-30 mnths	HPV & HIV: B & 6 mnthly	Prevalent HPV (vs HPV-negative)<br>Prevalent HR-HPV (vs HPV-negative)<br>Prevalent LR-HPV (vs HPV-negative)<br>1-2 prevalent HPV type (vs HPV-negative)<br>≥3 prevalent HPV types (vs HPV-negative)<br>Increase in HIV risk with number of HPV types	Incident HIV	2.55 (1.51,4.32)<br>2.04 (0.95,4.40)<br>2.74 (1.66,4.79)<br>1.70 (0.90,3.23)<br>4.92 (2.55,9.47)<br>1.33 (1.22,1.48)	HRR M<br>HRR M<br>HRR M<br>HRR M<br>HRR M<br>HRR M	1.72 (0.92,3.15)<br>1.25 (0.45,3.42)<br>1.92 (0.96,3.87)<br>1.41 (0.64,3.13)<br>3.45 (1.49,7.96)<br>1.27 (1.09,1.48)	HRR M<br>HRR M<br>HRR M<br>HRR M<br>HRR M<br>HRR M	5a,5a<br>5c,5c<br>5d,5d<br>--<br>--<br>--
**Uganda - Nested case-control in a circumcision trial in men in Rakai District**												

**Tobian (2013)(50)**	Range = 15-49	2004?-2006	831	24 mnths	HIV & HPV: at B, 6, 12 & 24 mnths	First incident HPV (vs Remaining HPV-negative) (unit=visit)	Incident HIV	2.72 (1.10,6.75)[1]	CRR M	--		5b,NA
						First incident HR-HPV (vs Remaining HPV-negative) (unit=visit)		2.60 (0.96,7.07)[1]	CRR M	1.49 (0.44,5.01)	OR M	--
						First incident LR-HPV (vs Remaining HPV-negative) (unit=visit)		3.04 (1.19,7.75)[1]	CRR M	2.21 (0.61,7.97)	OR M	--
						1 first incident HPV type (vs Remaining HPV-negative) (unit=visit)		1.17 (0.36,3.80)[1]	CRR M	1.27 (0.35,4.60)	OR M	--
						2 first incident HPV types (vs Remaining HPV-negative) (unit=visit)		3.84 (1.20,12.30)[1]	CRR M	2.90 (0.74,11.40)	OR M	--
						≥2 first incident HPV types (vs Remaining HPV-negative) (unit=visit)		5.17 (2.02,13.23)[1]	CRR M	--		--
						≥3 first incident HPV types (vs Remaining HPV-negative) (unit=visit)		6.39 (2.33,17.51)[1]	CRR M	2.71 (0.59,12.35)	OR M	--
						Clearance of any HPV type (vs HPV-negative) (unit=visit)		3.47 (1.46,8.23)[1]	CRR M	--		5f,NA
						Clearance of 1 HPV type (vs HPV-negative) (unit=visit)		1.71 (0.60,4.87)[1]	CRR M	--		--
						Clearance of 2 HPV types (vs HPV-negative) (unit=visit)		5.06 (1.91,13.43)[1]	CRR M	--		--
						Clearance of ≥3 HPV types (vs HPV-negative) (unit=visit)		5.76 (2.21,15.06)[1]	CRR M	--		--
						Persistent detection of all HPV types (vs HPV-negative) (unit=visit) (i.e. clearance = positive test followed by subsequent negative test		2.13 (0.54,8.36)[1]	CRR M	--		--
**Rwanda - Mainly FSWs in a prospective cohort in Kigali**												
**Veldhuijzen (2010)(51)**	Mean = 25	2006-2009	324	16.6 mnths	HPV: at 6 mnths (= B) & at yr 2 visit (data not used); HIV: at each visit (4 visits in yr 1 & 1 visit in yr 2)	Prevalent HPV (vs HPV-negative)	Incident HIV	2.54 (0.67,9.67)[1]	CRR F	--		5a,5a
						Prevalent HR-HPV (vs HPV-negative)		3.69 (0.97,13.94)[1]	CRR F	--		5c,5c
						Prevalent LR-HPV (vs HPV-negative)		2.03 (0.46,8.90)[1]	CRR F	--		5d,5d
						Prevalent HR-HPV (vs HR-HPV-negative)		4.70 (1.24,17.82)[1]	CRR F	--		--
						Prevalent LR-HPV (vs LR-HPV-negative)		1.28 (0.37,4.45)[1]	CRR F	--		--
**Tanzania & Uganda - Nested case-control in five cohort studies of women working in bars & recreational facilities**												
**Gallagher (2016)(52)**	Mean = 25	2002-2011	625	12-30 mnths	HIV & HPV: B & every 3-6 mnths	Prevalent HPV (vs HPV-negative)	Incident HIV	1.10 (0.84, 1.43)[1]	CRR	1.02 (0.66, 1.57)	OR	5a,5a
						Prevalent HPV-6, HPV-11, HPV-16, HPV-18, HPV-31, HPV-33, HPV-45, HPV-52 or HPV-58 (vs HPV-negative)		1.10 (0.78, 1.53)[1]	CRR	0.95 (0.56, 1.61)	OR	--
						Other prevalent HPV (vs HPV-negative)		1.10 (0.80, 1.50)[1]	CRR	1.08 (0.66, 1.78)	OR	--
						Prevalent HR-HPV only (vs HPV-negative)		1.13 (0.78, 1.64)[1]	CRR	1.15 (0.63, 2.08)	OR	--
						Prevalent HR-HPV (vs HPV-negative)		1.06 (0.78, 1.44)[1]	CRR	--		5c,NA
						Prevalent LR-HPV only (vs HPV-negative)		1.16 (0.82, 1.64)[1]	CRR	1.17 (0.66, 2.08)	OR	--
						Prevalent LR-HPV (vs HPV-negative)		1.08 (0.80, 1.45)[1]	CRR	--		5d,NA
						Prevalent HR-HPV & LR-HPV (vs HPV-negative)		0.98 (0.66, 1.47)[1]	CRR	0.78 (0.43, 1.44)	OR	--
						Clearance of any HPV type (vs HPV-negative)		1.19 (0.83, 1.71)[1]	CRR	0.85 (0.46, 1.55)	OR	5f,5f
						Persistent detection of all HPV types (vs HPV-negative)		0.97 (0.53, 1.80)[1]	CRR	0.93 (0.37, 2.29)	OR	--
						First incident HPV (vs Remaining HPV-negative) (i.e. clearance = positive test followed by subsequent negative test; persistence = detectable infection with same type for ≥ 6 mnths & no evidence of clearance of other types)		1.40 (0.88, 2.22)[1]	CRR	1.33 (0.61, 2.90)	OR	5b,5b
**Zimbabwe - Women in a trial MIRA of diaphragm & lubricant gel which showed no effect in Harare**												

**Smith-McCune (2010)(53)**	Mean = 27 (range 18-49)	2004-2006	2040	21 mnths (range 12-24)	HPV: 3 mnthly; HIV: 3 mnthly	Prevalent HPV (vs HPV-negative)	Incident HIV	1.84 (1.19,2.86)	HRR F	1.55 (0.99,2.42)	HRR F	5a,5a
						Prevalent HR-HPV (vs HPV-negative)		1.78 (1.10,2.86)	HRR F	1.56 (0.96,2.52)	HRR F	5c,5c
						Prevalent LR-HPV (vs HPV-negative)		1.52 (0.74,3.15)	HRR F	1.22 (0.58,2.55)	HRR F	5d,5d
						1 prevalent HPV type (vs HPV-negative)		NS & NR	--	NR	--	--
						≥2 prevalent HPV types (vs HPV-negative)		NS & NR	--	NR	--	--
						Prevalent HPV-16 (vs HPV-negative)		1.34 (0.54,3.30)	HRR F	1.27 (0.51,3.14)	HRR F	5e,5e
						Prevalent HPV-18 (vs HPV-negative)		0.49 (0.068[3],3.53)	HRR F	0.47 (0.06,3.37)	HRR F	5e,5e
						Prevalent HPV-31 (vs HPV-negative)		1.87 (0.46,7.60)	HRR F	1.92 (0.47,7.87)	HRR F	5e,5e
						Prevalent HPV-33 (vs HPV-negative)		1.82 (0.58,5.77)	HRR F	1.51 (0.47,4.80)	HRR F	5e,5e
						Prevalent HPV-52 (vs HPV-negative)		0.97 (0.13,6.94)	HRR F	0.86 (0.12,6.23)	HRR F	5e,5e
						Prevalent HPV-58 (vs HPV-negative)		2.58 (1.34,5.00)	HRR F	2.13 (1.09,4.15)	HRR F	5e,5e
						Clearance of all HR-HPV types (vs HPV-negative) (unit=type)		2.02 (1.47,3.98)	HRR F	1.67 (1.03,2.70)	HRR F	--
						Persistent detection of any HR-HPV type (vs HPV-negative) (unit=type)		1.01 (0.72,3.15)	HRR F	0.82 (0.45,1.50)	HRR F	--
						Clearance of all LR-HPV types (vs HPV-negative) (unit=type)		2.42 (1.26,3.25)	HRR F	2.09 (1.27,3.44)	HRR F	--
						Persistent detection of any LR-HPV type (vs HPV-negative) (unit=type)		1.50 (0.56,1.84)	HRR F	1.24 (0.59,2.60)	HRR F	--
						Recent[5] HPV infection (excl. current visit) (vs HPV-negative)		--		1.59 (0.97,2.58)[9]	HRR F	--
						Recent[5] HR-HPV infection (excl. current visit) (vs HPV-negative)		--		1.96 (1.16,3.30)[9]	HRR F	--
						Recent[5] LR-HPV infection (excl. current visit) (vs HPV-negative)		--		1.70 (1.02,2.85)[9]	HRR F	--
						Recent[5] HPV infection (incl. current visit) vs HPV-negative)[2]		--		1.63 (1.00,2.66)[9]	HRR F	--
						Recent[5] HR-HPV infection (incl. current visit) (vs HPV-negative)[2]		--		1.95 (1.19,3.21)[9]	HRR F	--
						Recent[5] LR-HPV infection (incl. current visit) (vs HPV-negative)[2] (i.e. persistence = 2 consecutive tests positive for a specific HPV type, or 2+ non-consecutive positive tests but not >3 consecutive negative tests (out of 2-6), or 3+ non-consecutive positive tests but not >3 consecutive negative tests (out of 7-9), or 4+ non-consecutive positive tests but not >3 consecutive negative tests (out of 10), or 4+ non-consecutive positive tests but not >4 consecutive negative tests (out of 11-12))		--		1.50 (0.92,2.43)[9]	HRR F	--
**Smith-McCune /Houlihan (2010/2012)(48, 53)**	Mean = 27 (range 18-49)	2004-2006	2040	21 mnths (range 12-24)	HPV: 3 mnthly; HIV: 3 mnthly	Prevalent HPV-16 or HPV-18[3] (vs HPV-16 & HPV-18 negative)	Incident HIV	--		0.84 (0.32,2.18)[6,10] 2.00 (1.00,3.99)[6,10]	HRR F	--
						Prevalent HPV-6, HPV-11, HPV-16 or HPV-18[3] (vs HPV-6, HPV-11, HPV-16 & HPV-18 negative)		--		2.57 (1.48,4.46)[6,10]	HRR F	--
						Prevalent HPV-6, HPV-11, HPV-16, HPV-18, HPV-31, HPV-33, HPV-45, HPV-52 or HPV-58[3] (vs HPV-6, HPV-11, HPV-16, HPV-18, HPV-31, HPV-33, HPV-45, HPV-52 & HPV-58 negative)		--		1.71 (1.00,2.92)[6]	HRR F	--
						Prevalent HPV[7] (vs HPV-negative)[2]		--			HRR F	--
**Burkina Faso - FSWs in a cohort**												
**Low/Houlihan (2011/2012)(48, 54)**	Range = 16-≥35	2003-2006	183	1.7 yrs	HPV: B; HIV: B & 4 mnthly with max. 6 visits	Prevalent HPV (vs HPV-negative)	Incident HIV	2.45 (0.26,24.85)[6]	HRR F	2.26 (0.23,22.60)[6]	HRR F	5a,5a
**South Africa - Women enrolled in a cervical cancer screening trial & extended study in Khayelitsha, Cape Town**												

**Wang (2011)(55)**	Mean = 42-44 (range 35-65)	2000-2005	5018	Range = 6-36 mnths	HPV: at 6, 12, 24 & 36 mnths; HIV: at 6, 12, 24 & 36 mnths – high possibility of reverse causality for effect of incident HPV & HPV clearance on HIV	First incident HR-HPV (vs Remaining HR-HPV-negative)[2]<br>Clearance of all HR-HPV types (vs Persistent detection of HR-HPV type)[2]<br>(i.e. clearance = negative HPV test during follow-up in those HPV-positive at B; required timing of negative test unclear)	Incident HIV	4.36 (2.48,7.64)<br>0.28 (0.07,1.15)	HRR F<br>HRR F	4.02 (2.26,7.13)<br>0.25 (0.06,1.03)	HRR F<br>HRR F	--<br>--
**Myer (2007)(56)**	Range = 35-49	2000-2002	4200	24 mnths	HPV: B; HIV: at 6, 12 & 24 mnths	Prevalent HPV[11] (vs HPV-negative)	Incident HIV	1.52 (1.02,2.28)	HRR F	1.31 (0.87,1.98)	HRR F	5a,5a
**Myer/Houlihan (2007/2012)(48, 56)**	Range = 35-49	2000-2002	4200	14.3 mnths	HPV: B; HIV: at 6, 12 & 24 mnths	Prevalent HR-HPV (vs HPV-negative)	Incident HIV	1.72 (1.25,2.35)[6]	HRR F	1.66 (1.21,2.28)[6]	HRR F	5c,5c
**Zimbabwe - Women in a nested case-control study part of a multicentre cohort study HC-HIV on hormonal contraception & risk of HIV**												
**Averbach (2010)(57)**	Mean = 25	1999-2004	591	21.9 mnths	HIV: B & at 3 mnths; HPV: at visit in which HIV srcnvrsn was detected & at visit prior to that when HIV-	Prevalent HPV (vs HPV-negative)<br>Prevalent HR-HPV (vs HPV-negative)<br>Prevalent LR-HPV only (vs HPV-negative)<br>1 prevalent HPV type (vs HPV negative)<br>2 prevalent HPV types (vs HPV negative)<br>3 prevalent HPV types (vs HPV negative)<br>≥4 prevalent HPV types (vs HPV negative)<br>Clearance of any HPV type (vs HPV-negative)<br><br>Persistent detection of all HPV types (vs HPV-negative)<br>(i.e. clearance = a positive 1st test and a negative 2nd test	Incident HIV	--<br>--<br>--<br>--<br>--<br>--<br>--<br>2.58 (1.91,3.48)[1]<br>0.92 (0.58,1.46)[1]	 <br><br><br><br><br><br>CRR F<br><br>CRR F	2.4 (1.5,4.0)<br>2.3 (1.4,3.9)<br>2.8 (1.3,5.9)<br>1.8 (1.0,3.3)<br>2.9 (1.4,5.9)<br>2.5 (1.0,6.0)<br>5.6 (2.5,12.9)<br>5.4 (2.9,9.9)<br><br>0.97 (0.51,1.85)	OR F<br>OR F<br>OR F<br>OR F<br>OR F<br>OR F<br>OR F<br>OR F<br><br>OR F	---<br>--<br>--<br>--<br>--<br>--<br>--<br>5f,5f<br><br>--
**Averbach/Houlihan (2010/2012)(48, 57)**	Mean = 25	1999-2004	591	21.9 mnths	HIV: B & at 3 mnths; HPV: at visit in which HIV srcnvrsn was detected & at visit prior to that when HIV-	Prevalent HPV-16 or HPV-18 (vs HPV-16 & HPV-18 negative)<br>Prevalent HPV-6, HPV-11, HPV-16 or HPV-18 (vs HPV-6, HPV-11, HPV-16 & HPV-18 negative)	Incident HIV	1.65 (0.96,2.72)<br>1.63 (0.98,2.72)	HRR F<br>HRR F	0.94 (0.46,1.92)<br>0.94 (0.47,1.84)	HRR F<br>HRR F	--<br>--

**Nowak (2011)(38)**	Mean = 25	1999-2004	641	21.9 mnths	HIV: B & at 3 mnths; HPV: at visit in which HIV srcnvrsn was detected & at visit prior to that when HIV-	Prevalent HPV (vs HPV-negative)	Incident HIV	1.69 (1.26,2.26)[1]	CRR F	--		5a,5a
						Prevalent HR-HPV (vs HPV-negative)		1.83 (1.36,2.47)[1]	CRR F	--		5c,5c
						Prevalent LR-HPV (vs HPV-negative)		2.02 (1.49,2.73)[1]	CRR F	--		5d,5d
						Prevalent HR-HPV (vs HR-HPV-negative)		1.74 (1.33,2.29)[1]	CRR F	--		--
						Prevalent LR-HPV (vs LR-HPV-negative)		1.94 (1.48,2.54)[1]	CRR F	--		--
						1 prevalent HPV (vs HPV-negative)		0.93 (0.60,1.43)[1]	CRR F	--		--
						≥2 prevalent HPV (vs HPV-negative)		2.29 (1.71,3.08)[1]	CRR F	--		--
						2 prevalent HPV (vs HPV-negative)		1.56 (1.01,2.41)[1]	CRR F	--		--
						3 prevalent HPV (vs HPV-negative)		2.90 (2.01,4.19)[1]	CRR F	--		--
						4-15 prevalent HPV (vs HPV-negative)		2.74 (1.93,3.88)[1]	CRR F	--		--
						New incident HPV (vs Remaining HPV-negative)		1.76 (1.28,2.41)[1]	CRR F	1.7 (1.2,2.3)	CRR F	5b,5b
						1 new incident HPV type (vs Remaining HPV-negative)		1.82 (1.28,2.58)[1]	CRR F	2.3 (1.4,3.9)	OR F	--
						≥2 new incident HPV types (vs Remaining HPV-negative)		1.63 (0.98,2.70)[1]	CRR F	1.4 (0.6,3.2)	OR F	--
**South Africa - FSWs in a trial Col-1492 of a vaginal gel in truck stops between Durban & Johannesburg**												
**Auvert (2011)(58)**	Mean = 24 (range 19-45)	1996-2000	88	2.5 yrs	HPV: 1st available test (not always at B) obtained before HIV srcnvrsn; HIV: B & 3 follow-up visits	≥2 prevalent HR-HPV types (vs ≤1 prevalent HR-HPV types)	Incident HIV	2.10 (1.11,3.95)[1]	CRR F	4.0 (1.2,14.0)	HRR F	--
						≥2 prevalent LR-HPV types (vs ≤1 prevalent LR-HPV types)		1.17 (0.56,2.42)[1]	CRR F	--		--
						Prevalent HR-HPV (vs HR-HPV-negative)		1.68 (0.71,3.99)[1]	CRR F	--		--
						Prevalent LR-HPV (vs LR-HPV-negative)		1.70 (0.79,3.64)[1]	CRR F	--		--
						Increase in HIV risk with number of HPV types		1.2 (0.97,1.4)	HRR F	1.1 (0.84,1.4)	HRR F	--
						Increase in HIV risk with number of LR-HPV types		1.1 (0.12,0.69)[4]	HRR F	0.77 (0.49,1.2)	HRR F	--
						Increase in HIV risk with number of HR-HPV types		1.5 (1.1,2.1)	HRR F	1.7 (1.01,2.7)	HRR F	--

Key: Studies are grouped by geographical region. For each region, studies carried out among both females and males are listed first, followed by those studies among males only, and then those studies among females only. Studies are then listed in order of study year, with the most recent listed first. Unit of infection is individuals, measured over the entire study period, unless otherwise specified as follows (unit=individual may still be specified for clarity where results presented for more than one unit of infection): visit – measurement done for individuals treating each interval between study visits separately; type – incidence/clearance of individual HPV types tracked. Individual as the unit of infection is equivalent to type as the unit of infection for incidence/clearance of individual HPV types. More than one mean/median age or follow-up duration is given where this was only presented for study subgroups. HR-HPV – high-risk HPV; LR-HPV – low-risk HPV; CI – confidence interval; HRR – hazard rate ratio; CRR – cumulative risk ratio; OR – odds ratio; U – unknown; NR – estimate not reported; NS – association reported as being not (statistically) significant; MSM – men who have sex with men; FSWs – female sex workers; FUP – follow-up; B – baseline; srcnvrtr/srcnvrsn – seroconverter/seroconversion. [1]Estimate and/or 95%CIs calculated from data given in the publication; [2]Not included in analysis; [3]Apparent error in published data, best estimate of correct value entered; [4]Apparent error in published data, data not used; [5]Within 6 months preceding HIV acquisition visit; [6]Unpublished; [7]At visit prior to seroconversion; [8]Adjusted for HR-HPV; [9]Adjusted additionally for time-dependent STI, condom use and sexual behaviour; [10]Adjusted additionally for other HPV types; [11]Could be prevalent HR-HPV rather than prevalent HPV; [12]Largest sample size of listed associations given: where this could not be calculated precisely because of partially overlapping subgroups or because these data were not available then the best estimate for the sample size was used.

Table S3. Additional study characteristics used to assess quality of studies from publications included in the review of longitudinal studies of the effect of HIV on HPV acquisition and clearance (review 1)

Source	Main Objective	HPV Assay	Sampling Site	HPV Types Detected in Assay	Sample Collection	Main Adjustments					Interview Method	Comparability in Selection/ Notes	Adjustment/Matching	Potential Publication Bias
						Circumcision	Condom Use	HC	No. of Partners	HSV-2				
**NORTH AMERICA**														
**Mullins, Moscicki (2013, 2004)(3, 4)**	To determine rates of incident anal HPV (overall & HR-HPV) & related sequelae, & factors associated with these outcomes, among HIV+ & higher risk HIV- adolescents; to compare HPV persistence by HIV status & risk factors for persistence	Dot-blot analysis for individual types & probe mixes; PCR	Anus; CLF	Mullins: HR-HPV: 16, 18, 31/33/35, 39, 45, 51, 52, 56, 58, 59/68/70; not HR-HPV: U; Moscicki: HR-HPV: 16, 31, 33, 35, 52, 58, 67, 18, 39, 45, 59, 68, 70, 26, 69, 51, 56, 53, 66; LR-HPV: 6, 11, 42, 44, 54, 40, 13, 32, 62, 72, 2, 57, 55	CN; HP	N	Y[1]	Y[1]	Y[1]	Y[1]	F-t-F INT & ACASI	Mullins: HIV+ individuals had more lifetime prtnrs than those HIV-, & more HIV+ M than HIV- reported sexual contact with another M & a history of receptive anal sex. More HIV+ than HIV- F had cervical HPV at B. Moscicki: HIV+ F had higher sexual risk behaviour at B, incl. younger age at first vaginal intercourse & higher no. of lifetime sexual prtnrs, & were more likely to have multiple HPV infections at B	Mullins: Matched for drug use behaviours; all HIV- adolescents sexually-active. No mention of HIV srcnvrtrs during FUP. 42% (current) & 58% (ever) ART use. Moscicki: F only; SS predictors from univariate analysis retained in multivariate model (univariate analysis done for multiple factors incl. age at first vaginal intercourse, age & HSV-2 (B), & CD4 count, NG, CT, TV, BV, HC, no. of lifetime prtnrs, condom use, viral load & ART (time-varying); stratification done by prevalent versus incident HPV infections). No HIV srcnvrtrs during FUP. 46% ART use	Only results for covariates which remained SS in the multivariate model reported
**Critchlow (1998)(5)**	To identify risk factors for the detection of prevalent anal HPV infection at study entry, detection/acquisition of additional HPV types during FUP & becoming HPV-negative during FUP, by HIV status	PCR & Southern transfer hybridization or HyC	Anus	HR-HPV: 16, 18; IR-HPV: 31, 33, 35; LR-HPV: 6, 11 (Southern transfer hybridization); HR-HPV: 16, 18, 45, 56; IR-HPV: 31, 33, 35, 39; LR-HPV: 6, 11, 42, 43, 44 (HyC)	HP	N?	Y?	NA	Y?	?	Enrolment INT then SAQ	HIV+ M had a higher no. of lifetime M sexual prtnrs & a younger age at first intercourse with a M prtnr, & were more likely to have reported unprotected anal sex in past 6 mnths. HIV+ M had a higher HPV prevalence at B	Multiple factors, time varying, self-reported STI. No mention of HIV srcnvrtrs during FUP. ART use. Very confusing as to how/what estimates were measured/reported	CD4 count reported as being NS associated with first incident HPV & estimates not given

Source	Main Objective	HPV Assay	Sampling Site	HPV Types Detected in Assay	Sample Collection	Main Adjustments					Interview Method	Comparability in Selection/ Notes	Adjustment/Matching	Potential Publication Bias
						Circumcision	Condom Use	HC	No. of Partners	HSV-2				
**Breese (1995)(6)**	To better characterize the type-specific prevalence & clinical spectrum of anal HPV infection & risk factors for its detection & persistence	Dot-filter hybridization assay	Penis	6, 11, 16, 18, 31, 33, 35	HP	-	-	-	-	-	INT	Similar wrt age, race, age at onset of sexual activity, no. of recent prtnrs, smoking history & history of urethral GC & penile warts. HIV+ M reported a higher median no. of lifetime prtnrs & a higher lifetime history of other STIs & were more likely to be HPV-positive at B	No mention of HIV srcnvrtrs during FUP. No ART use	Unlikely
**Dev (2006)(7)**	To determine the incidence of & risk factors associated with HPV & HR-HPV infection by HIV status	PCR & Southern blot hybridization	CLF	HR-HPV: 16, 18, 26, 31, 33, 35, 39, 45, 51, 52, 56, 58, 59, 68, 73, 82; LR-HPV: 6, 11, 40, 42, 53, 54, 55, 66, 68, 83, 84	HP	N?	Y	N?	Y	Y	INT	HIV+ F were more likely to have genital HPV infection at B & to be black	Multiple factors, time varying, self-reported; self-reported history of STIs at B. No HIV srcnvrtrs during FUP. No mention of ART use. In the abstract, they say the INTs, HIV & HPV testing occurred every 6 mnths, but this is not repeated in the methods	Unlikely

Source	Main Objective	HPV Assay	Sampling Site	HPV Types Detected in Assay	Sample Collection	Main Adjustments					Interview Method	Comparability in Selection/ Notes	Adjustment/Matching	Potential Publication Bias
						Circumcision	Condom Use	HC	No. of Partners	HSV-2				
**Blitz, Aho, Gagnon, Fontaine (2013, 2004, 2004, 2008)(8-11)**	To determine the effects of HIV status, ART use, CD4 T cell recovery, & other factors on the acquisition & clearance of HPV & the progression & regression of SIL; to investigate the association between the persistence of HPV infection & HPV-52 polymorphisms in a population either infected or at risk of HIV; to describe polymorphisms in the regulatory & transforming open-reading frames of HPV-33 & HPV-35 in a population either infected or at risk of HIV; to investigate the course of HPV-16 viral load in the natural history of HPV-16 infection in HIV+ F	Blitz: Primers/ radioactive probes & PGMY-line blot assay; Aho, Gagnon, Fontaine: PCR	Vagina & CLF	Blitz: 1994-2001: 14 types incl. 12 HR-HPV types: 16, 18, 31, 33, 35, 39, 45, 51, 52, 53, 56, 58; 2001-2003: 27 types incl. 13 HR-HPV types: 16, 18, 31, 33, 35, 39, 45, 51, 52, 53, 56, 58 & 59; Aho: 52 only; Gagnon: 33 & 35 only; Fontaine: 16 only	HP	N?	N ?	N?	N ?	N	Q	Blitz: HIV+ F were older, more likely to report injection drug use & more likely to have HR-HPV types & multiple HR-HPV types at B. HIV- F were more likely to report sex with another woman, to be currently sexually active, & to have used condoms consistently in past 6 mnths, & reported more lifetime sexual prtnrs. Aho, Gagnon & Fontaine: U	Blitz: 54% ART use. No mention of HIV srcnvrtrs during FUP. Aho: Age, LCR, & E6 variant. 4 F receiving ART. No mention of HIV srcnvrtrs during FUP. Gagnon & Fontaine: No mention of HIV srcnvrtrs during FUP. Only a few F on ART	Blitz: adjustment not done? Aho, Gagnon & Fontaine: some estimates reported as being NS but not presented. Only data for HPV-52, HPV-33, HPV-35 & HPV-16 reported
**Phelan, Ahdieh (2009, 2000)(12, 13)**	To investigate the hypothesis that increased risk for CIN in HIV+ F may be explained by repeated positivity of HPV infection facilitated by HIV infection & related immunosuppression; to explore the potential determinants of incident HPV infection by HIV status	PCR	CLF	26 types incl. 6, 11, 16, 18, 26, 31, 33, 35, 39, 40, 45, 51, 52, 53, 54, 55, 56, 58, 59, 66, 68, 73, 82, 83, 84	HP	N	N	N	Y	N	INT	HIV+ F were more likely to report a greater no. of M sex prtnrs in the last ten yrs. They were also more likely to have HPV at B. NS differences in race, age, recent drug use, recent sexual risk behaviours & ever contraceptive use	Phelan: Age, crack use in last 6 mnths, no. of M prtnrs in 10 yrs (NS covariates not included in multivariate model), time-varying. 44% ART use. HIV srcnvrtrs during FUP accounted for in incidence data as unit of analysis is visit? Ahdieh: 44% ART use. HIV/CD4 B for clearance data. No mention of HIV srcnvrtrs during FUP	Unlikely

Source	Main Objective	HPV Assay	Sampling Site	HPV Types Detected in Assay	Sample Collection	Main Adjustments					Interview Method	Comparability in Selection/ Notes	Adjustment/Matching	Potential Publication Bias
						Circumcision	Condom Use	HC	No. of Partners	HSV-2				
**Strickler, Watts, Viscidi, Silverberg, D'Souza, Strickler (2005, 2005, 2003, 2002, 2007, 2014)(14-19)**	To evaluate the association between current BV or TV infection over time & the natural history of HPV infection by HIV status; to determine the prevalence of serum IgA reactivity to HPV-16 capsids in relation to HIV status, cervicovaginal HPV infection & risk factors for HPV; to seek evidence of HPV reactivation by studying sexually-inactive F; to compare HPV-6 & HPV-11 infections & their relationship to genital warts in HIV+ & HIV- F & the role of HIV-associated immunosuppression; to estimate incidence rates for oral HPV, the factors influencing persistence, & to compare to those for cervical HPV; to assess the relationship between pDC & Treg levels in HIV+ F & oncHPV persistence	PCR	CLF	HR-HPV: 16, 18, 31, 45, IR-HPV: 33, 35, 39, 51, 52, 58, 59, 68, 73; LR-HPV: 6, 11, 40, 42, 53, 54, 61, 72, 81; not classified: 13, 26, 32, 34, 55, 56, 57, 62, 64, 66, 67, 69, 70, 71, 82, 83, 84, 85, 89, AE9, AE10; Strickler: HR-HPV: 16, 18, 31, 33, 35, 39, 45, 51, 52, 56, 58, 59 & 68	HP	N?	N?	N?	Y?[2]	Y[2]	INT	HIV+ F were older, less likely to have a current M sex prtnr & less likely to smoke than higher risk HIV- F. Past injection drug use was also more common & HIV+ F had a higher no. of recent sexual prtnrs. HIV+ F were more likely to be infected with HPV & to have multiple HPV infections at B	Strickler 2005: 35% on ART; 0.3% on ART (correct?). No mention of HIV srcnvrtrs during FUP. Watts: Multiple factors, time-varying incl. STIs (history of genital herpes measured but NS), matched on risk. 64% on ART. No mention of HIV srcnvrtrs during FUP. Viscidi & Silverberg: No mention of HIV srcnvrtrs during FUP. D'Souza: Age, tobacco use & CD4 count. 69% on ART. No mention of HIV srcnvrtrs during FUP. Convenience sample of WIHS cohort. Strickler 2014: Only HIV+ F with >350 CD4 included. 61% on ART. No mention of HIV srcnvrtrs during FUP	Possible selective reporting of SS adjusted estimates

Source	Main Objective	HPV Assay	Sampling Site	HPV Types Detected in Assay	Sample Collection	Main Adjustments					Interview Method	Comparability in Selection/ Notes	Adjustment/Matching	Potential Publication Bias
						Circumcision	Condom Use	HC	No. of Partners	HSV-2				
**Ahdieh, Koshiol, Viscidi (2001, 2006, 2005)(20-22)**	To investigate the effect of HIV infection & immunosuppression on the natural history of HPV; to analyse the time required to clear infections with specific HPV types & subgroups of HR-HPV types, by HIV status; to assess whether anti-VLP antibodies are a marker of immune protection, & their association with incident HPV infection	PCR/dot blot	CLF	Incidence: HR-HPV: 16, 18, 31, 45; IR-HPV: 33, 35, 39, 51, 52, 56, 58, 59, 68; LR-HPV: 6, 11, 26, 40, 42, 53, 54, 55, 66, 73, 82, 83, 84 Clearance: HR-HPV: includes 16, 18, 26, 31, 33, 35, 39, 45, 51, 52, 56, 58, 59, 66, 68, 73, 82; LR-HPV: includes 6, 11, 40, 42, 53,54, 55, 83, 84	HP	-	-	-	-	-	INT	HIV+ F were more likely to be HPV-positive at B	Ahdieh: No mention of ART use or HIV srcnvrtrs during FUP. Koshiol: No mention of ART use. F who srcnvrtd during FUP were excluded. Viscidi: No mention of ART use or HIV srcnvrtrs during FUP	Unlikely
**Sun (1997)(23)**	To determine the gynaecologic characteristics associated with HIV infection	PCR	CLF	25 types? "Intermediate-risk": 51, 53	HP	-	-	-	-	-	INT	Similar in terms of age, race or ethnic group, education, income, no. of lifetime prtnrs, condom use & age at first sexual intercourse. HIV+ F were less likely to be married, & more likely to have a history of prostitution, & have been sexually abstinent in the preceding mnth	No ART use. No mention of HIV srcnvrtrs during FUP	Unlikely
**Minkoff (1998)(24)**	To study the relationship of HIV serostatus & immune status to HPV carriage	PCR	CLF	Multiple incl. 2, 6, 11, 13, 16, 18, 26, 31, 32, 33, 34, 35, 39, 40, 42, 45, 51, 52, 53, 54, 55, 56, 57, 58, 59, 61, 62, 64, 66, 67, 68, 69, 70, 72, 73, AE2, AE5-8, W13B, PAP291, PAP155	HP	-	-	-	-	-	INT	U. HIV- F were followed up for longer. HIV+ F had higher HPV prevalence at B	No ART use? No mention of HIV srcnvrtrs during FUP. HIV+ F rolled over into WIHS at end of study	Estimates for effect on LR-HPV not presented despite LR-HPV types being tested
**LATIN AMERICA & THE CARIBBEAN**														

Source	Main Objective	HPV Assay	Sampling Site	HPV Types Detected in Assay	Sample Collection	Main Adjustments					Interview Method	Comparability in Selection/ Notes	Adjustment/Matching	Potential Publication Bias
						Circumcision	Condom Use	HC	No. of Partners	HSV-2				
**Silva (2011)(25)**	To assess the persistence & clearance of HPV DNA by HIV status	PCR/Roche line blot hybridization test	Penis	37 types	HP	-	-	-	-	-	INT	SS differences across several variables	No mention of ART use. No mention of HIV srcnvrtrs during FUP	Unlikely
**Jalil (2013)(26)**	To evaluate HPV clearance in the postpartum period of HIV+ & -negative F	PCR	CLF	HR-HPV: 16, 18, 31, 33; LR-HPV: 6, 11	HP	NA	N	N	N	N	U	HIV+ F were older, had longer FUP, had more lifetime sexual prtnrs & were more likely to be HPV-positive at B than HIV- F	Age, smoking status, mode of delivery, nature of HPV infection (e.g. clinically-defined) & HPV type HR-HPV vs LR-HPV). No mention of ART use. No mention of HIV srcnvrtrs during FUP. Not adjusted for previous STIs or sexual behaviour	Unlikely
**Ceccato Junior (2016)(27)**	To evaluate and compare CIN & HPV incidence by HIV status	PCR-based	Cervix	Not all HPV types listed. HR-HPV: 16, 18, 31, 33, 35, 39, 45, 51, 52, 53, 55, 56, 58, 59, 66, 67, 68, 69, 70, 73, 82, 83	U	-	-	-	-	-	U	U	63.7% on ART. No mention of HIV srcnvrtrs during FUP	Unlikely
**WESTERN EUROPE**														
**Mooij (2016)(28)**	To compare anal & penile HR-HPV incidence & clearance by HIV status in MSM, & to evaluate the effect of HIV-related immunosuppression on rates	SPF_{10}-PCR DEIA/LiPA25 system (version 1)	Anus & penis	25 types incl HR-HPV: 16, 18, 31, 33, 35, 39, 45, 51, 52, 56, 58, 59	SC	Y	N	NA	Y	N	SAQ	HIV+ MSM were SS older & had SS more lifetime M sex prtnrs at B	Age, circumcision status & no. of lifetime M sex prtnrs (B), smoking, no. of recent anal sex prtnrs, recent cannabis &/or poppers use, anal sex position, having been rimmed, receptive fisting, anal/urethral STIs (CT &/or GC) & HPV type (all time-varying). Not all adjustments implemented for all estimates. 87% ART use. No mention of HIV srcnvrtrs during FUP	Unlikely

Source	Main Objective	HPV Assay	Sampling Site	HPV Types Detected in Assay	Sample Collection	Main Adjustments					Interview Method	Comparability in Selection/ Notes	Adjustment/Matching	Potential Publication Bias
						Circumcision	Condom Use	HC	No. of Partners	HSV-2				
**van der Snoek (2005)(29)**	To study clearance & acquisition of anal HPV infection & to investigate possible differences by HIV status	PCR then PCR-reverse hybridization test (SPF LiPA)	Anus	Initially: HR-HPV: 16, 18, 31, 33; LR-HPV: 6, 11; Subsequently: HR-HPV: 16, 18, 31, 33, 35, 39, 45, 51, 52, 53 (probably), 56, 58, 59, 66 (probably), 68; LR-HPV: 6, 11, 34, 40, 42, 43, 44, 54, 70, 74; "unclassified"	HP	-	-	-	-	-	SAQ	Similar wrt age, ethnicity, education, age at sexual debut & no. of prtnrs in past 6 mnths. HIV+ MSM were more likely to have had genital warts & had a higher B HPV prevalence than HIV- MSM	5/17 HIV+ M (incl. HIV srcnvrtrs during FUP) on ART	Effect on clearance of HPV-31 reported as being SS, statistical significance of effect on other types NR & no estimates presented
**Tornesello (2008)(30)**	To determine the prevalence & persistence of HPV by HIV status & by cytological/histological diagnosis, & the effects of immunosuppression & HIV viral load on HPV	PCR/sequencing	Cervix	Several but U	HP	-	-	-	-	-	INT	Similar in terms of age, race/ethnic group & education HIV+ F were more likely to have an abnormal smear & be HPV-positive at B	56% ART use. No mention of HIV srcnvrtrs during FUP	Unlikely
**Branca (2003)(31)**	To prospectively follow F with & without HIV infection to investigate the clinical course of HPV infections as well as factors predicting the persistence & clearance of HPV & PAP smear abnormality	PCR & restriction-fragment-length-polymorphism; sequencing of some swabs	Cervix	U	HP	-	-	-	-	-	INT	SS differences across several variables incl. greater proportion using condoms among those HIV+, & higher HPV prevalence & likelihood of abnormal smear at B among HIV+ F	All HIV+ treated with ART. No mention of HIV srcnvrtrs during FUP	HIV SS associated with HPV-positivity at end of FUP & NS associated with oncogenic HPV type at end of FUP, however estimates not presented
**SUB-SAHARAN AFRICA**														
**Mbulawa (2012)(32)**	To investigate the rate of new HPV detection & clearance in a cohort of heterosexual couples & the factors associated with new HPV detection & HPV clearance	Roche Linear Array HPV genotyping assay	Cervix/ penis	HR-HPV: 16, 18, 26, 31, 33, 35, 39, 45, 51, 52, 53, 56, 58, 59, 66, 68, 73, 82; LR-HPV: 6, 11, 40, 42, 54, 55, 61, 62, 64, 67, 69, 70, 71, 72, 81, 83, 84, 89, IS39	U	N	N	N	N	N	U	U	Type-specific incidence/ clearance rates. 5.4% of HIV+ F & 7.5% of HIV+ M on ART. 3 HIV srcnvrtrs during FUP (excluded)	Unlikely

Source	Main Objective	HPV Assay	Sampling Site	HPV Types Detected in Assay	Sample Collection	Main Adjustments					Interview Method	Comparability in Selection/ Notes	Adjustment/Matching	Potential Publication Bias
						Circumcision	Condom Use	HC	No. of Partners	HSV-2				
**Tobian, Grabowski (2012, 2014)(33, 34)**	To measure risk factors for HR-HPV incidence & clearance by HIV status; to assess HR-HPV viral load & persistence among HIV- & HIV+ M in a M circumcision trial	Roche HPV linear array	Penis	HR-HPV: 16, 18, 31, 33, 35, 39, 45, 51, 52, 56, 58, 59, 66, 68	HP	Y	N ?	NA	Y	N ?	INT	HIV+ M were older, more likely to report genital ulcer disease & urethral discharge, more likely to be HSV-2 seropositive & more likely to have multiple HR-HPV types detected at B	Tobian: HIV srcnvrtrs during FUP excluded (very few with HPV data). M with CD4<3501 or WHO stage 4 disease excluded. HIV+ M referred for treatment. Grabowski: HPV viral load, circumcision status, sexual prtnrs in last 12 mnths & age. HIV srcnvrtrs during FUP excluded. No ART?	HRR for effect on 2+ HPV types reported as being SS & estimate given; estimate for effect on 1 HPV type NR
**Adler (2015)(35)**	To compare rates of HR-HPV persistence between HIV+ and HIV- young F	Roche linear array HPV test	Vagina	37 HPV types of which HR-HPV: 16, 18, 31, 33, 35, 39, 45, 51, 52, 56, 58, 59, 68	SC	-	-	-	-	-	U	HIV+ youth slightly older, but there was no difference in no. of sexual prtnrs in last 6 mnths	9/33 on ART. No mention of HIV srcnvrtrs during FUP	Unlikely
**Banura (2010)(36)**	To report findings on incidence, clearance & risk factors for HPV infections	HPV DNA enzyme immunoassay	Cervix	HR-HPV: 16, 18, 31, 33, 35, 39, 45, 51, 52, 56, 58, 59, 68/73; LR-HPV: 6, 11, 34, 40, 42, 43, 44, 53, 54, 66, 70, 74; additional HPV types: 26, 30, 55, 61, 62, 64, 67, 69, 71, 82, 83, 84, 85, 87, 89, 90, 91	MW	N	N	N	Y	N	Q	HIV+ F were more likely to be infected with HPV at B	Age & lifetime no. of sexual prtnrs. HIV+ referred for treatment. No mention of HIV srcnvrtrs during FUP	Unlikely
**Banura (2008)(37)**	To evaluate prevalence, clearance & incidence of HPV between the 1st/2nd & 3rd trimesters of pregnancy, & between pregnancy & delivery	PCR & DNA enzyme immunoassay / reverse hybridization assay	Cervix	HR-HPV: 16, 18, 31, 33, 35, 39, 45, 51, 52, 56, 58, 59, 68/73; LR-HPV: 6, 11, 34, 40, 42, 43, 44, 53, 54, 66, 70, 74	MW	N	N	N	N	N	Q	HIV+ F were more likely to be infected with HPV at B	Age. HIV+ referred for treatment. No mention of HIV srcnvrtrs during FUP	Unlikely

Source	Main Objective	HPV Assay	Sampling Site	HPV Types Detected in Assay	Sample Collection	Main Adjustments					Interview Method	Comparability in Selection/ Notes	Adjustment/Matching	Potential Publication Bias
						Circumcision	Condom Use	HC	No. of Partners	HSV-2				
**Nowak (2011)(38)**	To investigate whether newly detected HPV infections would SS increase after HIV infection	Roche Linear Array HPV genotyping assay	Cervix	HR-HPV: 16, 18, 31, 33, 35, 39, 45, 51, 52, 56, 58, 59, 66, 68; LR-HPV: 6, 11, 26, 40, 42, 53, 54, 55, 61, 62, 64, 67, 69, 70, 71, 72, 73, 81, 82, IS39, 83, 84, 89	HP	?	N[3]	N[3]	Y	N[3]	Q	F who acquired HIV (exposure) were less likely to be in a monogamous marriage, living with their primary prtnr & using a form of HC, & more likely to have more than one lifetime prtnr & to have a higher risk primary prtnr. They were also more likely to have NG, BV & HSV-2, & to have HPV & multiple HPV infections at B Lifetime no. of sex prtnrs & primary prtnr risk, time-varying matched on time in study, age & STI variable	Lifetime no. of sex prtnrs & primary prtnr risk, time-varying matched on time in study, age & STI variable	Unlikely
**Safaeian (2008)(39)**	To investigate the incidence & clearance of HPV using SC vaginal swabs	HyC then Roche reverse hybridization assay	Vagina	HR-HPV: 16, 18, 31, 33, 35, 39, 45, 51, 52, 56, 58, 59, 68	SC	N	N	N	Y	N	INT	U	Lifetime no. of prtnrs & age, time-varying. No mention of ART use or HIV srcnvrtrs during FUP	Unlikely
**Rowhani-Rahbar (2007)(40)**	To assess the impact of HIV status & type on HPV clearance, & to explore the role played by CD4 count & HIV load	PCR or PCR-based reverse-line strip assay (Roche)	U	Before 01/04/1998: HR-HPV: 16, 18, 31, 33, 35, 39, 45, 56, 51, 52; LR-HPV: 6, 11; After 01/04/1998: HR-HPV: 16, 18, 26, 31, 33, 35, 39, 45, 51, 52, 55, 56, 58, 59, 68, 73, 82, 83; LR-HPV: 6, 11, 40, 42, 53, 54, 57, 66, 84	HP	N	Y	Y	Y	N	INT	Majority of HIV- F were infected with HR-HPV types due to over-sampling. HIV+ F were less likely to use a condom & more likely to have multiple HPV infections at B Age, parity, smoking, being a SW, new sexual prtnrs in past 4 mnths, HPV type, CD4 cell count;	Age, parity, smoking, being a commercial sex worker, new sexual prtnrs in past 4 mnths, HPV type, CD4 count; time-varying. No ART use. No mention of HIV srcnvrtrs during FUP	Data by CD4 count not presented. Crude estimates not presented for effect on LR-HPV & HR-HPV, while only crude estimates reported for effect on HPV-16 & -18. No estimates for effect on types besides HPV-16 & HPV-18 reported

Source	Main Objective	HPV Assay	Sampling Site	HPV Types Detected in Assay	Sample Collection	Main Adjustments: Circumcision	Condom Use	HC	No. of Partners	HSV-2	Interview Method	Comparability in Selection/ Notes	Adjustment/Matching	Potential Publication Bias
**Miotti (1996)(41)**	To establish the association between SIL, HPV & HIV & if this association is affected by HIV-related immunosuppression, to quantitate HPV DNA & correlate it with immunosuppression & to investigate whether oncogenic HPV types persist longer	PCR & Southern blot	CLF	6, 11, 16, 18, 31, 33 & 35	HP	-	-	-	-	-	SAQ?	Similar in age, oral contraceptive use & STD diagnosis. HIV+ F were more likely to have a history of multiple sex prtnrs, genital warts & cervical ectopy, & more likely to be HPV-positive at B	No ART use? No mention of HIV srcnvrtrs during FUP	Unlikely
**EAST ASIA & PACIFIC**														
**Phanuphak, Phanuphak (2013, 2013)(42, 43)**	To study the prevalence, incidence & persistence of anal HPV in a cohort of MSM with & without HIV infection; to study high-grade AIN prevalence, incidence & associated predictors in a cohort of MSM with & without HIV infection	Linear Array HPV Genotyping Test (Roche) (PCR)	Anus	6, 11, 16, 18, 26, 31, 33, 35, 39, 40, 42, 45, 51, 52, 53, 54, 55, 56, 58, 59, 61, 62, 64, 66, 67, 68, 69, 70, 71, 72, 73 (MM9), 81, 82 (MM4), 83 (MM7), 84 (MM8), IS39, CP6108. HR-HPV included 16, 18, 31, 33, 35, 39, 45, 51, 52, 56, 58, 59, 68	CN	N	Y	NA	Y	N	U	HIV+ M had more lifetime sexual prtnrs & fewer recent sexual prtnrs than HIV- M, & had higher prevalence of HPV & of HR-HPV at B	No. of recent sexual prtnrs, no. of sex acts, condom use, age & age at sexual debut. 13% ART use. No mention of HIV srcnvrtrs during FUP	Selective reporting of some unadjusted & adjusted estimates on basis of statistical significance

Key: Studies are grouped by geographical region. For each region, studies carried out among both females & males are listed first, followed by those studies among males only, and then those studies among females only. Studies are then listed in order of study year, with the most recent listed first. HR-HPV – high-risk HPV; LR-HPV – low-risk HPV; IR-HPV; – intermediate-risk HPV; HC – hormonal contraception; HSV-2 – Herpes Simplex Virus type 2; MSM – men who have sex with men; HyC – Hybrid capture; PCR – polymerase chain reaction; BV – bacterial vaginosis; TV – *Trichomonas vaginalis*; NG – *Neisseria gonorrhoeae*; CT – *Chlamydia trachomatis*; IgA – immunoglobulin A; VLP – virus-like particle; SAQ – self-administered Q; ACASI – audio computer-assisted systematic interview; NA – Not applicable; CLF - Cervicovaginal lavage fluid; HP – health professional; CN – clinician; MW – midwife; SC – self-collected; Q – questionnaire; srcnvrtr/srcnvrsn – seroconverter/seroconversion; INT – interview; U – Unknown; FUP – follow-up; F-t-F – face-to-face; wrt – with respect to; AIN - anal intraepithelial neoplasia; B – baseline; NR – not reported; NS – association reported as being not (statistically) significant; SS – association reported as being statistically significant. [1]Association examined in univariate analysis; only statistically significant predictors retained in multivariate model; [2]For data from Watts (2005)(15); [3]Not included in final model as not informative.

Table S4. Additional study characteristics used to assess quality of studies from publications included in the review of longitudinal studies of the effect of HPV infection and clearance on HIV (review 2)

Source	Main Objective	HPV Assay	Sampling Site	HPV Types Detected in Assay	Sample Collection	Main Adjustments					Interview Method	Comparability in Selection/ Notes	Adjustment/Matching	Potential Publication Bias
						Circumcision	Condom Use	HC	No. of Partners	HSV-2				
**NORTH AMERICA**														
**Chin-Hong (2009)(44)**	To determine the association between anal HPV & HIV acquisition	PCR-based	Anus	HR-HPV: 16, 18, 31, 33, 35, 39, 45, 51, 52, 56, 58, 59, 68, 73; LR-HPV: 6, 11, 53, 54, 66, 83, 84	TS	N	Y	NA	Y	Y[1]	ACASI	U	Adjustment done for multiple factors; time varying & self-reported incl. history of STIs (self-report of HSV included)	Only SS associations reported
**SUB-SAHARAN AFRICA**														
**Auvert (2010)(45)**	To explore the association of HR-HPV & LR-HPV with HIV incidence	PCR, Roche Linear Array HPV genotyping assay	Urethra	HR-HPV: 16, 18, 31, 33, 35, 39, 45, 51, 52, 56, 58, 59, 68; LR-HPV: 6, 11, 26, 40, 42, 53, 54, 55, 61, 62, 64, 66, 67, 69, 70, 71, 72, 73, 81, 82, 83, 84, IS39, CP6108	NU	Y[2]	Y[2]	N[2]	Y[2]	Y[2]	U	Individuals with HPV at B older. B HPV also associated with higher no. of lifetime sexual prtnrs. HR-HPV & LR-HPV correlated at B	Adjustment done for multiple factors; time-varying but bacterial STIs/TV measured at 21 mnths whilst HSV-2 measured at each FUP visit	Crude estimate for effect of LR-HPV & its dose-response NR
**Smith, Smith/Houlihan, Rositch (2010, 2010/2012, 2014)(46, 48, 49)**	To prospectively measure the incidence of HIV in HPV-positive compared to HPV-negative individuals; to determine if young M with current, persistent or recently-cleared HPV are at a higher risk of HIV acquisition, & to measure the potential modifying effect of circumcision	PCR-based	Penis	6, 11, 16, 18, 26, 30, 31, 32, 33, 34, 35, 39, 40, 42, 43, 44, 45, 51, 52, 53, 54, 55, 56, 57, 58, 59, 61, 64, 66, 67, 68, 69, 70, 71, 72, 73, 81, 82, 83, 84, cand85, 86, cand89, JC9710; HR-HPV: 16, 18, 31, 33, 35, 39, 45, 51, 52, 56, 58, 59, 66, 68	HP	Y	Y	NA	?	Y	SAQ	HPV presence at B associated with genital warts, HSV-2 seropositivity, GC infection, CT infection, no condom use & greater no. of lifetime F prtnrs	Smith: Adjusted for variables measured at B: circumcision status (also measured as time-varying covariate), age, employment status & HSV-2 infection, among other factors. Smith/Houlihan: Adjusted for circumcision status, age, employment status, B HSV-2 infection & other HPV types, among other factors. Rositch: Time-varying variables: circumcision status, HSV-2 status, sexual behaviour in past 6 mnths; B measures: age, education & employment status. Circumcision also added as an interaction term (little evidence of effect modification)	Unlikely

**Tobian (2013)(50)**	To assess the associations between HPV acquisition & clearance & HIV srcnvrsn in M, & the cellular immune correlates of HPV acquisition & clearance	HPV Linear Array Genotyping Test (Roche)	Penis	37 types incl. HR-HPV: includes 16, 18, 31, 33, 35, 39, 45, 51, 52, 56, 58, 59, 66, 68	TS	Y	Y	NA	Y	Y	INT	U	Adjusted for age, marital status, circumcision status, no. of sex prtnrs during past interval, non-marital relationships, condom use, HSV-2, symptoms of STIs	Unlikely
**Veldhuijzen (2010)(51)**	To investigate the association between HR-HPV infection & subsequent HIV acquisition	PCR-based	Cervix	HR-HPV: 16, 18, 31, 33, 35, 39, 45, 51, 52, 56, 58, 59, 68, 73, 82, iso39; LR-HPV: remaining types	HP	-	-	-	-	-	U	U	HSV-2 measured at enrolment, mnth 12 & mnth 24, BV measured at enrolment, mnth 12 & mnth 24 but adjustment not done due to too few HIV incident cases	OR reported only for effect of HR-HPV although data on individuals presented for both HR-HPV & LR-HPV
**Gallagher (2016)(52)**	To investigate the association between cervical HPV infection & HIV acquisition	Roche linear array HPV test	Cervix or CVL	HR-HPV: 16, 18, 31, 33, 35, 39, 45, 51, 52, 56, 58, 59, 68; LR-HPV: 6, 11, 26, 40, 42, 53, 54, 55, 61, 62, 64, 66, 67, 69, 70, 71, 72, 73, 81, 82, IS39 (combined with 82), 83, 84, CP6108 (89)	U	NA	N	N	N	Y	INT	HIV srcnvrsn associated with younger age, higher no. of lifetime prtnrs, having paid for sex in past 3 mnths, drinking alcohol more frequently, completed primary education, experienced forced sex in past 3 mnths (NS) CT, NG & HSV-2	Matched on time. Adjusted for age, alcohol consumption at enrolment (B), transactional sex in 3 mnths prior to first detection of HIV (time-varying), CT, NG & HSV-2 (all time-varying)	Unlikely
**Smith-McCune, Smith-McCune/ Houlihan (2010, 2010/ 2012)(48, 53)**	To determine the association between HPV infection & HIV acquisition	PCR-based	Cervix or vagina	6, 11, 16, 18, 26, 31, 32, 33, 35, 39, 40, 45, 51, 52, 53, 54, 55, 56, 58, 59, 61, 66, 68, 69, 70, 73, 83, 84, 82v, 2, 13, 34, 42, 57, 62, 64, 67, 72, 82	CN & SC	In prt nr	Y	N	N	Y	Q	U	Smith-McCune: Adjustment done for multiple B factors, incl. STIs (incl. HSV-2), condom use & sexual behaviour. Prevalent, cumulative or persistent/non-persistent HPV status fixed variable; concurrent or recent HPV infection time-dependent variable. Smith-McCune/Houlihan: Adjustment done for multiple factors, incl. B STI (incl. HSV-2), condom use & sexual behaviour	Dose-response reported as being NS & estimates not presented

**Low/ Houlihan (2011/ 2012)(48, 54)**	To determine the prevalence, incidence & persistence of genital warts, & their association with HIV infection, immunosuppression & other risk factors	U	U	U	U	N?	N?	N?	N?	Y	INT	U	Adjusted for HSV-2 & other HPV types	Unlikely
**Wang (2011)(55)**	To evaluate the risk of HPV & cytologic abnormalities occurring among F with incident HIV infection	HyC & Roche Linear Array HPV genotyping assay	Cervix	HR-HPV: 16, 18, 31, 33, 35, 39, 45, 51, 52, 56, 58, 59, 68	HP	N	N?	N?	Y	N?	U	U	Adjustment done for demography, CT/NG/TV (at B), sexual behaviour during previous mnth. Not adjusted for HSV-2	Unlikely
**Myer, Myer/ Houlihan (2007, 2007/ 2012)(48, 56)**	To investigate the associations between different hormonal contraceptive methods & increased susceptibility to HIV	HyC	Cervix	U	HP	N	Y	Y	Y	N	Q	U	Myer: Adjustment done for demography, CT/NG/TV (at B), sexual behaviour during previous mnth. Not adjusted for HSV-2. Results also reported for 6 mnths FUP. Myer/Houlihan: Adjustment done for demography, CT/NG/TV (at B), sexual behaviour during the previous mnth. Not adjusted for HSV-2	Unlikely
**Averbach, Averbach/ Houlihan, Nowak (2010, 2010/ 2012, 2011)(38, 48, 57)**	To examine whether pre-existing HPV infection is independently associated with HIV acquisition; to investigate whether newly-detected HPV would SS increase immediately after HIV acquisition	HPV Linear Array (Roche)	Cervix	HR-HPV: 16, 18, 31, 33, 35, 39, 45, 51, 52, 56, 58, 59, 66, 68; LR-HPV: 6, 11, 26, 40, 42, 53, 54, 55, 61, 62, 64, 67, 69, 70, 71, 72, 73, 81, 82, IS39, 83, 84, 89	CN	NA	Y	Y	Y	Y	INT or Q	U	Averbach: Matching (2-4 controls per case) to time in study, age group, composite STI/genital tract infection variable; adjustment done for living with a prtnr, behavioural risks & primary prtnr's behavioural risk, HC, condom use, HSV-2, GC, CT, TV, BV, & candidiasis. Analysis for prevalent HPV done for HPV at visit before HIV srcnvrsn was detected; analysis for loss of detection of HPV done for HPV between visit before & visit at detection of HIV srcnvrsn – possibility of reverse causation for loss of detection of HPV. Averbach/Houlihan: As above & additionally adjusted for the presence of other HPV genotypes. Nowak: Matching & adjustment as for Averbach, above. Analysis done for HPV at visit before HIV srcnvrsn was detected	Only covariates which substantially changed any of the effect measures included in final multivariate model
**Auvert (2011)(58)**	To assess HPV as a risk factor for HIV acquisition	Reverse line blot assay (Roche)	CLF	HR-HPV: 16, 18, 31, 33, 35, 39, 45, 51, 52, 56, 58, 59, 68; LR-HPV: 6, 11, 26, 40, 42, 53, 54, 55, 57, 66, 73, 82, 83, 84	HP	N	Y	N	Y	N	U	U	Adjustment done for multiple factors incl. presence of other HPV types, demography, condom use, sexual behaviour, TV/NG/CT/syphilis; all using B data incl. for STIs	Unlikely

Key: Studies are grouped by geographical region. For each region, studies carried out among both females and males are listed first, followed by those studies among males only, and then those studies among females only. Studies are then listed in order of study year, with the most recent listed first. HR-HPV – high-risk HPV; LR-HPV – low-risk HPV; HC – hormonal contraception; HSV-2 – Herpes Simplex Virus type 2; HyC – Hybrid capture; PCR

– polymerase chain reaction; BV – bacterial vaginosis; TV – *Trichomonas vaginalis*; NG – *Neisseria gonorrhoeae*; CT – *Chlamydia trachomatis*; OR – odds ratio; SAQ – self-administered Q; ACASI – audio computer-assisted systematic interview; NA – Not applicable; CLF - Cervicovaginal lavage fluid; HP – health professional; CN – clinician; TS – trained staff; NU – nurse; SC – self-collected; Q – questionnaire; INT – interview; U – Unknown; FUP – follow-up; B – baseline; NR – not reported; NS – association reported as being not (statistically) significant; SS – association reported as being statistically significant; srcnvrtr/srcnvrsn – seroconverter/seroconversion. [1]By self-report; [2]For effect of HR-HPV on HIV.

Table S5. Subgroup analyses of the association between prior exposure to HPV and subsequent HIV infection by participant and study characteristics (review 2). Pooled crude RR and adjusted aRR are presented for the following HPV exposure definitions: a) prevalent HPV; b) incident HPV; c) prevalent HR-HPV; d) incident HR-HPV; e) Clearance of HPV. Estimates in bold are statistically-significantly different to 1 (p value<0.05)

Study and participant characteristics	Crude pooled				Adjusted pooled			
	N	RR (95%CI)	I^2 (%)	p value[a] (X^2)	N	aRR (95%CI)	I^2 (%)	p value[a] (X^2)
**a. Prevalent HPV**								
**Sex**								
Males	2	**3.60 (1.72, 7.55)**	65.8	0.087	2	**2.75 (1.06, 7.13)**	75.6	0.043
Females	6	**1.49 (1.20, 1.86)**	32.4	0.193	5	**1.48 (1.08, 2.02)**	43.3	0.133
**Region**								
Sub-Saharan Africa (Low- and middle-income economies[b])	8	**1.91 (1.38, 2.65)**	70.8	0.001	7	**1.75 (1.23, 2.49)**	62.7	0.013
**Risk population[c]**								
MSM only	-	-	-	-	-	-	-	-
Heterosexual men only	2	**3.60 (1.72, 7.55)**	65.8	0.087	2	**2.75 (1.06, 7.13)**	75.6	0.043
Higher risk populations	3	1.14 (0.88, 1.48)	0.0	0.385	2	1.05 (0.69, 1.61)	-	-
Lower risk populations	5	**2.12 (1.51, 2.99)**	66.4	0.018	5	**1.96 (1.34, 2.85)**	62.1	0.032
**Study year (midpoint)**								
1997-2006	7	**1.89 (1.35, 2.66)**	74.5	0.001	7	**1.75 (1.23, 2.49)**	62.7	0.013
≥2007	1	2.54 (0.67, 9.67)	-	-	-	-	-	-
**Measure of association**								
ALL	8	**1.91 (1.38, 2.65)**	70.8	0.001	7	**1.75 (1.23, 2.49)**	62.7	0.013
HRR	5	**2.34 (1.51, 3.62)**	62.4	0.031	5	**1.87 (1.22, 2.86)**	84.8	0.010
CRR	3	1.42 (0.96, 2.211)	63.8	0.063	-	-	-	-
OR	-	-	-	-	2	1.55 (0.67, 3.59)	-	-
**Unit of analysis**								
Individual-level	8	**1.91 (1.38, 2.65)**	70.8	0.001	7	**1.75 (1.23, 2.49)**	62.7	0.013
**Key variables adjusted for**								
**HSV-2**								
Yes	-	-	-	-	6	**1.89 (1.23, .2.89)**	66.2	0.011
No	-	-	-	-	1	1.31 (0.87, 1.98)	-	-
**Number of sexual partners**								
Yes	-	-	-	-	2	**3.13 (1.67, 5.80)**	52.4	0.147
No	-	-	-	-	5	**1.33 (1.06, 1.68)**	0.0	0.597
**Hormonal contraception**								
Yes	-	-	-	-	1	**2.40 (1.47, 3.92)**	-	-
No	-	-	-	-	6	**1.64 (1.12, 2.42)**	61.8	0.022
**Male circumcision**								
Yes	-	-	-	-	3	**2.20 (1.19, 4.05)**	69.6	0.037
No	-	-	-	-	4	1.47 (0.96, 2.27)	56.8	0.074
**Condom use**								
Yes	-	-	-	-	4	**2.21 (1.45, 3.38)**	57.0	0.073
No	-	-	-	-	3	1.18 (0.88, 1.58)	0.0	0.609
**Comparing subset with crude and adjusted**	6	**1.99 (1.28, 3.12)**	78.6	<0.001	6	**1.64 (1.12, 2.42)**	61.8	0.022
**Risk of publication bias**								
Estimate reported in study	5	**2.34 (1.51, 3.62)**	62.4	0.031	7	**1.75 (1.23, 2.49)**	62.7	0.013
Estimate derived from study information	3	1.42 (0.96, 2.11)	63.8	0.063	-	-	-	-
**b. Incident HPV**								
**Sex**								
Males	1	**2.72 (1.10, 6.75)**	-	-	-	-	-	-
Females	2	**1.64 (1.26, 2.12)**	0.0	0.419	2	**1.64 (1.21, 2.21)**	0.0	0.569
**Incidence definition**								
First incident HPV infection	2	1.73 (0.94, 3.19)	39.4	0.199	-	-	-	-
New incident HPV infection	1	**1.76 (1.28, 2.41)**	-	-	1	**1.70 (1.23, 2.35)**	-	-
**Measure of association**								
ALL	3	**1.70 (1.32, 2.18)**	0.0	0.412	2	**1.64 (1.21, 2.21)**	0.0	0.569
CRR	3	**1.70 (1.32, 2.18)**	0.0	0.412	1	**1.70 (1.23, 2.35)**	-	-
OR					1	1.33 (0.61, 2.21)	-	-
**Key variables adjusted for**								
**HSV-2**								
Yes	-	-	-	-	2	**1.64 (1.21, 2.21)**	0.0	0.569
No	-	-	-	-	-	-	-	-
**Number of sexual partners**								

Yes	-	-	-	-	1	**1.70 (1.23, 2.35)**	-	-
No	-	-	-	-	1	1.33 (0.61, 2.21)	-	-
**Hormonal contraception**								
Yes	-	-	-	-	1	**1.70 (1.23, 2.35)**	-	-
No	-	-	-	-	1	1.33 (0.61, 2.21)		
**Male circumcision**								
Yes	-	-	-	-	-	-	-	-
No	-	-	-	-	2	**1.64 (1.21, 2.21)**	0.0	0.569
**Condom use**								
Yes	-	-	-	-	1	**1.70 (1.23, 2.35)**	-	-
No	-	-	-	-	1	1.33 (0.61, 2.21)		
**Comparing subset with crude and adjusted**	2	**1.64 (1.26, 2.12)**	0.0	0.419	2	**1.64 (1.21, 2.21)**	0.0	0.569
**c. Prevalent HR-HPV**								
**Sex**								
Males	1	2.04 (0.95, 4.39)	-	-	1	1.25 (0.45, 3.45)	-	-
Females	5	**1.60 (1.21, 2.11)**	58.0	0.049	3	**1.75 (1.39, 2.22)**	0.0	0.492
**Region**								
Sub-Saharan Africa (i.e. Low- and middle-income economies[b])	6	**1.63 (1.26, 2.09)**	50.0	0.075	4	**1.72 (1.37, 2.17)**	0.0	0.610
**Risk population[c]**								
MSM only	-	-	-	-	-	-	-	-
Heterosexual men only	1	**2.72 (1.10, 6.75)**	-	-	-	-	-	-
Higher risk populations	2	1.66 (0.51, 5.36)	68.8	0.073	-	-	-	-
Lower risk populations	4	**1.79 (1.48, 2.17)**	0.0	0.978	4	**1.72 (1.37, 2.17)**	0.0	0.610
**Study year (midpoint)**								
1997-2006	5	**1.58 (1.23, 2.03)**	0.0	0.078	4	**1.72 (1.37, 2.17)**	0.0	0.610
≥2007	1	3.69 (0.97, 13.94)	-	-	-	-	-	-
**Measure of association**								
ALL	6	**1.63 (1.26, 2.09)**	50.0	0.075	4	**1.72 (1.37, 2.17)**	0.0	0.610
HRR	3	1.57 (0.93, 2.65)	75.9	0.016	3	**1.60 (1.24, 2.07)**	0.0	0.865
CRR	3	**1.77 (1.38, 2.27)**	0.0	0.921	-	-	-	-
OR	-	-	-	-	1	**2.30 (1.38, 3.84)**	-	-
**Unit of analysis**								
Individual	6	**1.63 (1.26, 2.09)**	50.0	0.075	4	**1.72 (1.37, 2.17)**	0.0	0.610
**Key variables adjusted for**								
**HSV-2**								
Yes	-	-	-	-	3	**1.79 (1.29, 2.50)**	0.0	0.424
No	-	-	-	-	1	**1.66 (1.21, 2.28)**	-	-
**Number of sexual partners**								
Yes	-	-	-	-	2	**1.83 (1.37, 2.46)**	11.2	0.289
No	-	-	-	-	2	1.50 (0.97, 2.32)	0.0	0.699
**Hormonal contraception**								
Yes	-	-	-	-	2	**1.83 (1.37, 2.46)**	11.2	0.289
No	-	-	-	-	2	1.50 (0.97, 2.32)	0.0	0.699
**Male circumcision**								
Yes	-	-	-	-	2	1.50 (0.97, 2.32)	0.0	0.699
No	-	-	-	-	2	**1.83 (1.37, 2.46)**	11.2	0.289
**Condom use**								
Yes	-	-	-	-	4	**1.72 (1.37, 2.17)**	0.0	0.610
No	-	-	-	-	-	-	-	-
**Comparing subset with crude and adjusted**	3	**1.77 (1.38, 2.27)**	0.0	0.921	3	**1.60 (1.23, 2.07)**	0.0	0.865
**Risk of publication bias**								
Estimate reported in study	3	**1.77 (1.38, 2.27)**	0.0	0.921	4	**1.72 (1.37, 2.17)**	0.0	0.610
Estimate derived from study information	3	1.57 (0.93, 2.65)	75.9	0.016	-	-	-	-
**d. Incident HR-HPV**								
First incident HR-HPV	1	2.60 (0.96, 7.07)	-	-	1	1.49 (0.45, 5.01)	-	-
**e. Clearance of HPV**								
**Sex**								
Males	1	**3.47 (1.46, 8.24)**	-	-	-	-	-	-
Females	2	1.76 (0.82, 3.77)	90.5	0.001	2	2.14 (0.35, 13.11)	94.3	0.000
**Clearance definition**								
Loss of detection of all HR-HPV types	-	-	-	-	-	-	-	-
Loss of detection of any HR-HPV type	3	**2.07 (1.10, 3.90)**	83.7	0.020	2	2.14 (0.35, 13.11)	94.3	0.000
**Measure of association**								

ALL	3	**2.07 (1.10, 3.90)**	83.7	0.020	2	2.14 (0.35, 13.11)	94.3	0.000
CRR	3	**2.07 (1.10, 3.90)**	83.7	0.020	-	-	-	-
OR	-	-	-	-	2	2.14 (0.35, 13.11)	94.3	0.000
**Key variables adjusted for**								
**HSV-2**								
Yes	-	-	-	-	2	2.14 (0.35, 13.11)	94.3	<0.001
No	-	-	-	-	-	-	-	-
**Number of sexual partners**								
Yes	-	-	-	-	2	2.14 (0.35, 13.11)	94.3	<0.001
No	-	-	-	-	-	-	-	-
**Hormonal contraception**								
Yes	-	-	-	-	1	**5.40 (2.92, 9.98)**	-	-
No	-	-	-	-	1	0.85 (0.46, 1.56)	-	-
**Male circumcision**								
Yes	-	-	-	-	-	-	-	-
No	-	-	-	-	2	2.14 (0.35, 13.11)	94.3	<0.001
**Condom use**								
Yes	-	-	-	-	1	**5.40 (2.92, 9.98)**	-	-
No	-	-	-	-	1	0.85 (0.46, 1.56)	-	-
**Comparing subset with crude and adjusted**	2	1.76 (0.82, 3.77)	90.5	0.001	2	2.14 (0.35, 13.11)	94.3	<0.001

Key: [a]Relates to I^2; [b]World Bank definition; [c]MSM only: studies which only included men who have sex with men; Higher risk populations: studies which included female sex workers (FSWs), MSM, injecting drug users (IDUs) or STI clinic attendees, or studies consisting of participants reporting higher risk sex practices; Lower risk populations are participants from couples studies, antenatal care (ANC) clinics, or other general population samples.

APPENDIX REFERENCES

1. Sterne JAC, Harbord RM. Funnel plots in meta-analysis. Stata J. 2004;4(2):127-41.
2. Egger M, Davey Smith G, Schneider M. Bias in meta-analysis detected by a simple, graphical test. BMJ. 1997;315(7109):629-34.
3. Mullins TL, Wilson CM, Rudy BJ, Sucharew H, Kahn JA. Incident anal human papillomavirus and human papillomavirus-related sequelae in HIV-infected versus HIV-uninfected adolescents in the United States. Sexually transmitted diseases. 2013;40(9):715-20.
4. Moscicki AB, Ellenberg JH, Farhat S, Xu J. Persistence of human papillomavirus infection in HIV-infected and -uninfected adolescent girls: risk factors and differences, by phylogenetic type. The Journal of infectious diseases. 2004;190(1):37-45.
5. Critchlow CW, Hawes SE, Kuypers JM, Goldbaum GM, Holmes KK, Surawicz CM, et al. Effect of HIV infection on the natural history of anal human papillomavirus infection. AIDS. 1998;12(10):1177-84.
6. Breese PL, Judson FN, Penley KA, Douglas JM, Jr. Anal human papillomavirus infection among homosexual and bisexual men: prevalence of type-specific infection and association with human immunodeficiency virus. Sexually transmitted diseases. 1995;22(1):7-14.
7. Dev D, Lo Y, Ho GY, Burk RD, Klein RS. Incidence of and risk factors for genital human papillomavirus infection in women drug users. J Acquir Immune Defic Syndr. 2006;41(4):527-9.
8. Blitz S, Baxter J, Raboud J, Walmsley S, Rachlis A, Smaill F, et al. Evaluation of HIV and highly active antiretroviral therapy on the natural history of human papillomavirus infection and cervical cytopathologic findings in HIV-positive and high-risk HIV-negative women. The Journal of infectious diseases. 2013;208(3):454-62.
9. Aho J, Hankins C, Tremblay C, Forest P, Pourreaux K, Rouah F, et al. Genomic polymorphism of human papillomavirus type 52 predisposes toward persistent infection in sexually active women. The Journal of infectious diseases. 2004;190(1):46-52.
10. Gagnon S, Hankins C, Tremblay C, Forest P, Pourreaux K, Coutlee F. Viral polymorphism in human papillomavirus types 33 and 35 and persistent and transient infection in the genital tract of women. The Journal of infectious diseases. 2004;190(9):1575-85.
11. Fontaine J, Hankins C, Money D, Rachlis A, Pourreaux K, Ferenczy A, et al. Human papillomavirus type 16 (HPV-16) viral load and persistence of HPV-16 infection in women infected or at risk for HIV. Journal of clinical virology : the official publication of the Pan American Society for Clinical Virology. 2008;43(3):307-12.
12. Phelan DF, Gange SJ, Ahdieh-Grant L, Mehta SH, Kirk GD, Shah K, et al. Determinants of newly detected human papillomavirus infection in HIV-infected and HIV-uninfected injection drug using women. Sexually transmitted diseases. 2009;36(3):149-56.
13. Ahdieh L, Munoz A, Vlahov D, Trimble CL, Timpson LA, Shah K. Cervical neoplasia and repeated positivity of human papillomavirus infection in human immunodeficiency virus-seropositive and -seronegative women. American journal of epidemiology. 2000;151(12):1148-57.
14. Strickler HD, Burk RD, Fazzari M, Anastos K, Minkoff H, Massad LS, et al. Natural history and possible reactivation of human papillomavirus in human immunodeficiency virus-positive women. Journal of the National Cancer Institute. 2005;97(8):577-86.
15. Watts DH, Fazzari M, Minkoff H, Hillier SL, Sha B, Glesby M, et al. Effects of bacterial vaginosis and other genital infections on the natural history of human papillomavirus infection in HIV-1-infected and high-risk HIV-1-uninfected women. The Journal of infectious diseases. 2005;191(7):1129-39.
16. Viscidi RP, Ahdieh-Grant L, Schneider MF, Clayman B, Massad LS, Anastos KM, et al. Serum immunoglobulin A response to human papillomavirus type 16 virus-like particles in human immunodeficiency virus (HIV)-positive and high-risk HIV-negative women. The Journal of infectious diseases. 2003;188(12):1834-44.

17. Silverberg MJ, Ahdieh L, Munoz A, Anastos K, Burk RD, Cu-Uvin S, et al. The impact of HIV infection and immunodeficiency on human papillomavirus type 6 or 11 infection and on genital warts. Sexually transmitted diseases. 2002;29(8):427-35.
18. D'Souza G, Fakhry C, Sugar EA, Seaberg EC, Weber K, Minkoff HL, et al. Six-month natural history of oral versus cervical human papillomavirus infection. International journal of cancer Journal international du cancer. 2007;121(1):143-50.
19. Strickler HD, Martinson J, Desai S, Xie X, Burk RD, Anastos K, et al. The relation of plasmacytoid dendritic cells (pDCs) and regulatory T-cells (Tregs) with HPV persistence in HIV-infected and HIV-uninfected women. Viral immunology. 2014;27(1):20-5.
20. Ahdieh L, Klein RS, Burk R, Cu-Uvin S, Schuman P, Duerr A, et al. Prevalence, incidence, and type-specific persistence of human papillomavirus in human immunodeficiency virus (HIV)-positive and HIV-negative women. The Journal of infectious diseases. 2001;184(6):682-90.
21. Koshiol JE, Schroeder JC, Jamieson DJ, Marshall SW, Duerr A, Heilig CM, et al. Time to clearance of human papillomavirus infection by type and human immunodeficiency virus serostatus. International journal of cancer Journal international du cancer. 2006;119(7):1623-9.
22. Viscidi RP, Snyder B, Cu-Uvin S, Hogan JW, Clayman B, Klein RS, et al. Human papillomavirus capsid antibody response to natural infection and risk of subsequent HPV infection in HIV-positive and HIV-negative women. Cancer epidemiology, biomarkers & prevention : a publication of the American Association for Cancer Research, cosponsored by the American Society of Preventive Oncology. 2005;14(1):283-8.
23. Sun XW, Kuhn L, Ellerbrock TV, Chiasson MA, Bush TJ, Wright TC, Jr. Human papillomavirus infection in women infected with the human immunodeficiency virus. The New England journal of medicine. 1997;337(19):1343-9.
24. Minkoff H, Feldman J, DeHovitz J, Landesman S, Burk R. A longitudinal study of human papillomavirus carriage in human immunodeficiency virus-infected and human immunodeficiency virus-uninfected women. American journal of obstetrics and gynecology. 1998;178(5):982-6.
25. Silva RJ, Casseb J, Andreoli MA, Villa LL. Persistence and clearance of HPV from the penis of men infected and non-infected with HIV. Journal of medical virology. 2011;83(1):127-31.
26. Jalil EM, Bastos FI, Melli PP, Duarte G, Simoes RT, Yamamoto AY, et al. HPV clearance in postpartum period of HIV-positive and negative women: a prospective follow-up study. BMC infectious diseases. 2013;13:564.
27. Ceccato Junior BP, Guimaraes MD, Lopes AP, Nascimento LF, Novaes LM, Del Castillo DM, et al. Incidence of Cervical Human Papillomavirus and Cervical Intraepithelial Neoplasia in Women with Positive and Negative HIV Status. Revista brasileira de ginecologia e obstetricia : revista da Federacao Brasileira das Sociedades de Ginecologia e Obstetricia. 2016;38(5):231-8.
28. Mooij SH, van Santen DK, Geskus RB, van der Sande MA, Coutinho RA, Stolte IG, et al. The effect of HIV infection on anal and penile human papillomavirus incidence and clearance: a cohort study among MSM. Aids. 2016;30(1):121-32.
29. van der Snoek EM, Niesters HG, van Doornum GJ, Mulder PG, Osterhaus AD, van der Meijden WI. Acquisition and clearance of perianal human papillomavirus infection in relation to HIV-positivity in men who have sex with men in the Netherlands. Acta dermato-venereologica. 2005;85(5):437-43.
30. Tornesello ML, Duraturo ML, Giorgi-Rossi P, Sansone M, Piccoli R, Buonaguro L, et al. Human papillomavirus (HPV) genotypes and HPV16 variants in human immunodeficiency virus-positive Italian women. The Journal of general virology. 2008;89(Pt 6):1380-9.
31. Branca M, Garbuglia AR, Benedetto A, Cappiello T, Leoncini L, Migliore G, et al. Factors predicting the persistence of genital human papillomavirus infections and PAP smear abnormality in HIV-positive and HIV-negative women during prospective follow-up. International journal of STD & AIDS. 2003;14(6):417-25.

32. Mbulawa ZZ, Marais DJ, Johnson LF, Coetzee D, Williamson AL. Impact of human immunodeficiency virus on the natural history of human papillomavirus genital infection in South african men and women. The Journal of infectious diseases. 2012;206(1):15-27.
33. Tobian AA, Kigozi G, Gravitt PE, Xiao C, Serwadda D, Eaton KP, et al. Human papillomavirus incidence and clearance among HIV-positive and HIV-negative men in sub-Saharan Africa. AIDS. 2012;26(12):1555-65.
34. Grabowski MK, Gray RH, Serwadda D, Kigozi G, Gravitt PE, Nalugoda F, et al. High-risk human papillomavirus viral load and persistence among heterosexual HIV-negative and HIV-positive men. Sexually transmitted infections. 2014;90(4):337-43.
35. Adler D, Wallace M, Bennie T, Abar B, Sadeghi R, Meiring T, et al. High risk human papillomavirus persistence among HIV-infected young women in South Africa. International journal of infectious diseases : IJID : official publication of the International Society for Infectious Diseases. 2015;33:219-21.
36. Banura C, Sandin S, Van Doorn LJ, Quint W, Kleter B, Wabwire-Mangen F, et al. Type-specific incidence, clearance and predictors of cervical human papillomavirus infections (HPV) among young women: A prospective study in Uganda. Infectious agents and cancer. 2010;5(1).
37. Banura C, Franceschi S, van Doorn LJ, Arslan A, Kleter B, Wabwire-Mangen F, et al. Prevalence, incidence and clearance of human papillomavirus infection among young primiparous pregnant women in Kampala, Uganda. International journal of cancer Journal international du cancer. 2008;123(9):2180-7.
38. Nowak RG, Gravitt PE, Morrison CS, Gange SJ, Kwok C, Oliver AE, et al. Increases in human papillomavirus detection during early HIV infection among women in Zimbabwe. The Journal of infectious diseases. 2011;203(8):1182-91.
39. Safaeian M, Kiddugavu M, Gravitt PE, Gange SJ, Ssekasanvu J, Murokora D, et al. Determinants of incidence and clearance of high-risk human papillomavirus infections in rural Rakai, Uganda. Cancer epidemiology, biomarkers & prevention : a publication of the American Association for Cancer Research, cosponsored by the American Society of Preventive Oncology. 2008;17(6):1300-7.
40. Rowhani-Rahbar A, Hawes SE, Sow PS, Toure P, Feng Q, Dem A, et al. The impact of HIV status and type on the clearance of human papillomavirus infection among Senegalese women. The Journal of infectious diseases. 2007;196(6):887-94.
41. Miotti PG, Dallabetta GA, Daniel RW, Canner JK, Chiphangwi JD, Liomba GN, et al. Cervical abnormalities, human papillomavirus, and human immunodeficiency virus infections in women in Malawi. The Journal of infectious diseases. 1996;173(3):714-7.
42. Phanuphak N, Teeratakulpisarn N, Pankam T, Kerr SJ, Barisri J, Deesua A, et al. Anal human papillomavirus infection among Thai men who have sex with men with and without HIV infection: prevalence, incidence, and persistence. J Acquir Immune Defic Syndr. 2013;63(4):472-9.
43. Phanuphak N, Teeratakulpisarn N, Triratanachat S, Keelawat S, Pankam T, Kerr SJ, et al. High prevalence and incidence of high-grade anal intraepithelial neoplasia among young Thai men who have sex with men with and without HIV. AIDS. 2013;27(11):1753-62.
44. Chin-Hong PV, Husnik M, Cranston RD, Colfax G, Buchbinder S, Da Costa M, et al. Anal human papillomavirus infection is associated with HIV acquisition in men who have sex with men. AIDS. 2009;23(9):1135-42.
45. Auvert B, Lissouba P, Cutler E, Zarca K, Puren A, Taljaard D. Association of oncogenic and nononcogenic human papillomavirus with HIV incidence. J Acquir Immune Defic Syndr. 2010;53(1):111-6.
46. Smith JS, Moses S, Hudgens MG, Parker CB, Agot K, Maclean I, et al. Increased risk of HIV acquisition among Kenyan men with human papillomavirus infection. The Journal of infectious diseases. 2010;201(11):1677-85.
47. Bailey RC, Moses S, Parker CB, Agot K, Maclean I, Krieger JN, et al. Male circumcision for HIV prevention in young men in Kisumu, Kenya: a randomised controlled trial. Lancet. 2007;369(9562):643-56.

48. Houlihan CF, Larke NL, Watson-Jones D, Smith-McCune KK, Shiboski S, Gravitt PE, et al. Human papillomavirus infection and increased risk of HIV acquisition. A systematic review and meta-analysis. AIDS. 2012;26(17):2211-22.
49. Rositch AF, Mao L, Hudgens MG, Moses S, Agot K, Backes DM, et al. Risk of HIV acquisition among circumcised and uncircumcised young men with penile HPV infection. AIDS. 2013.
50. Tobian AA, Grabowski MK, Kigozi G, Redd AD, Eaton KP, Serwadda D, et al. Human papillomavirus clearance among males is associated with HIV acquisition and increased dendritic cell density in the foreskin. The Journal of infectious diseases. 2013;207(11):1713-22.
51. Veldhuijzen NJ, Vyankandondera J, van de Wijgert JH. HIV acquisition is associated with prior high-risk human papillomavirus infection among high-risk women in Rwanda. AIDS. 2010;24(14):2289-92.
52. Gallagher KE, Baisley K, Grosskurth H, Vallely A, Kapiga S, Vandepitte J, et al. The Association Between Cervical Human Papillomavirus Infection and Subsequent HIV Acquisition in Tanzanian and Ugandan Women: A Nested Case-Control Study. The Journal of infectious diseases. 2016;214(1):87-95.
53. Smith-McCune KK, Shiboski S, Chirenje MZ, Magure T, Tuveson J, Ma Y, et al. Type-specific cervico-vaginal human papillomavirus infection increases risk of HIV acquisition independent of other sexually transmitted infections. PloS one. 2010;5(4):e10094.
54. Low AJ, Clayton T, Konate I, Nagot N, Ouedraogo A, Huet C, et al. Genital warts and infection with human immunodeficiency virus in high-risk women in Burkina Faso: a longitudinal study. BMC infectious diseases. 2011;11:20.
55. Wang C, Wright TC, Denny L, Kuhn L. Rapid rise in detection of human papillomavirus (HPV) infection soon after incident HIV infection among South African women. The Journal of infectious diseases. 2011;203(4):479-86.
56. Myer L, Denny L, Wright TC, Kuhn L. Prospective study of hormonal contraception and women's risk of HIV infection in South Africa. Int J Epidemiol. 2007;36(1):166-74.
57. Averbach SH, Gravitt PE, Nowak RG, Celentano DD, Dunbar MS, Morrison CS, et al. The association between cervical human papillomavirus infection and HIV acquisition among women in Zimbabwe. AIDS. 2010;24(7):1035-42.
58. Auvert B, Marais D, Lissouba P, Zarca K, Ramjee G, Williamson AL. High-risk human papillomavirus is associated with HIV acquisition among South African female sex workers. Infectious diseases in obstetrics and gynecology. 2011;2011:692012.
